# Early mortality in atezolizumab/bevacizumab for HCC is associated with impaired liver function and alterations of systemic immunity

**DOI:** 10.1016/j.jhepr.2025.101513

**Published:** 2025-07-05

**Authors:** Ignazio Piseddu, Leonie S. Jochheim, Katrin Boettcher, Bernhard Scheiner, Friedrich Sinner, Simon Johannes Gairing, Matthias Thaler, Stefan Enssle, Monika Karin, Valentina Zarka, Alexander Philipp, Andreas Thalmeier, Jan Gaertig, Lorenz Balcar, Julia Martina Schütte, Julia S. Schneider, Katarina Ondrejkova, Monika Rau, Alexander Weich, David Anz, Karin Berger, Christian Schulz, Christian M. Lange, Osman Öcal, Marianna Alunni-Fabbroni, Jens Ricke, Ursula Ehmer, Marino Venerito, Friedrich Foerster, Matthias Pinter, Andreas Geier, Julia Mayerle, Enrico N. De Toni, Florian P. Reiter, Najib Ben Khaled

**Affiliations:** 1 Department of Medicine II, University Hospital, LMU Munich, Munich, Germany; 2 Division of Clinical Pharmacology, Department of Medicine II, University Hospital, LMU Munich, Munich, Germany; 3 Partner site – German Alliance for Liver Cancer (GALC), Heidelberg, Germany; 4 Department of Gastroenterology and Hepatology, University Hospital Essen, University of Duisburg-Essen, Essen, Germany; 5 TUM School of Medicine and Health, Department of Clinical Medicine – Clinical Department for Internal Medicine II, University Medical Center, Technical University of Munich, Germany; 6 Division of Gastroenterology and Hepatology, Department of Medicine III, Medical University of Vienna, Vienna, Austria; 7 Department of Gastroenterology, Hepatology and Infectious Diseases, Otto-von-Guericke University Hospital Magdeburg, Magdeburg, Germany; 8 Department of Medicine I, University Medical Center of the Johannes-Gutenberg University Mainz, Mainz, Germany; 9 Department of Medicine III, University Hospital, LMU Munich, Munich, Germany; 10 Pharmacy, University Hospital, LMU Munich, Munich, Germany; 11 Division of Hepatology, Department of Medicine II, University Hospital Würzburg, Würzburg, Germany; 12 Department of Radiology, University Hospital, LMU Munich, Munich, Germany; 13 Bavarian Cancer Research Center (BZKF), Erlangen, Germany

**Keywords:** Hepatocellular carcinoma, Immunotherapy, Atezolizumab, Bevacizumab, Early mortality

## Abstract

**Background & Aims:**

Atezolizumab/bevacizumab (atezo/bev) has revolutionized the standard of care for patients with unresectable hepatocellular carcinoma (HCC). However, only a subgroup of patients responds to atezo/bev and derives durable clinical benefit. This study aims to analyze the frequency and risk factors of early mortality (EM) in patients with HCC treated with atezo/bev.

**Methods:**

This study uses data from a large, European real-world cohort and flow cytometry-based immunophenotyping of patient’s baseline PBMC. EM was defined as death from any cause within 90 days of treatment initiation. Logistic regression analysis was used to identify parameters associated with EM.

**Results:**

A total of 317 patients with unresectable HCC treated with first-line atezo/bev were included. EM rate in the cohort was 15.8%, with a median survival of 12.6 months. The proportion of patients with preserved liver function and BCLC stage B was significantly lower in the EM cohort. The strongest predictor of EM was advanced liver disease in univariate analysis, as reflected by surrogates of impaired liver function such as Child–Pugh score (CPS) B (*p* <0.0001), albumin–bilirubin grade 2/3 (*p* = 0.026, *p* <0.0001) or high model for end-stage liver disease score (*p* <0.0001). CPS B remained a significant risk factor after adjusting for other variables. Biomarker analysis and immunophenotyping revealed high C-reactive protein, reduced lymphocyte frequencies, increased CD44 expression on regulatory T cells and elevated PD-L1 levels on CD8^+^ T cells to be associated with EM.

**Conclusions:**

EM rate was 15.8% in patients with HCC treated with atezo/bev. Significant risk factors for early death involved impaired liver function, elevated biomarkers of inflammation and alterations of systemic immunity.

**Impact and implications:**

Although rare in clinical trial populations, early mortality (EM) is a significant concern following the initiation of immune checkpoint inhibitors in patients with hepatocellular carcinoma (HCC) in real-world cohorts, highlighting the need for adequate risk stratification and patient selection. Using data of a large, European real-world cohort as well as FACS-based immunophenotyping of baseline peripheral blood mononuclear cells, we comprehensively characterized the frequency, risk factors, and biomarkers associated with EM in atezo/bev-treated patients with HCC. We identified EM to represent a common event in patients with HCC after treatment initiation and demonstrated impaired liver function, elevated biomarkers of inflammation, and reduced lymphocyte frequencies and increased regulatory T cell activity to be associated with increased EM risk. These findings can help clinicians identify patients with HCC at high risk of EM, enabling adequate and informed decision-making regarding end-of-life care and palliative treatment.

## Highlights

• Early mortality occurs in 15.8% of patients with HCC treated with atezo/bev.
• Impaired liver function is a key predictor of early mortality.
• High CRP, reduced lymphocyte frequencies and increased Treg activity are associated with increased mortality risk.
• Findings might help clinicians to identify patients at high risk of early death.

## Graphical abstract

**Figure:**
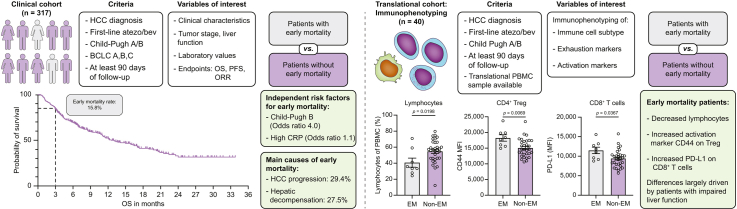

## Introduction

Hepatocellular carcinoma (HCC) ranks among the most lethal cancer entities worldwide, with approximately 800,000 deaths in 2020.^1^^,^^2^ The approval of the immune checkpoint inhibitor (ICI) atezolizumab in combination with the vascular endothelial growth factor (VEGF) inhibitor bevacizumab (atezo/bev) has revolutionized the treatment landscape for unresectable HCC, significantly improving patient outcomes as compared with the previous first-line standard sorafenib as demonstrated in the pivotal IMbrave150 trial.^3^^,^^4^ However, real-world cohorts tend to be sicker than study populations, especially in HCC. Most patients have chronic liver disease and individuals treated in clinical practice frequently have more severe liver dysfunction as compared with trial participants.^5^

One significant concern in this regard is early mortality (EM) following the initiation of immunotherapy.^6^ EM is rare in clinical trial populations. In the IMbrave150 study, around 95% of patients were still alive 3 months after therapy initiation.^4^ However, because of strict eligibility criteria, these patients represent a highly selected cohort. Real-world data from other cancer entities suggests a higher incidence of EM upon ICI administration. In this context, a Canadian study reported 60-day and 90-day mortality rates as high as 15% and 22% in various cancers, excluding HCC.^7^ A similar dimension of early death was seen in two retrospective analyses of lung cancer patients, with rates reported to be 15%^8^ and 13%.^9^ These findings suggest that patients in real-world settings have a higher risk of EM compared with those in clinical trials. To date, the frequency and risk factors of early death in patients with HCC receiving atezo/bev have not been thoroughly investigated. Understanding these factors could improve patient selection and monitoring, enhancing treatment safety and ensuring that atezo/bev is allocated to those most likely to benefit.

In this multicenter study, we aimed to assess the characteristics of patients with HCC experiencing EM after initiation of atezo/bev and identify clinical characteristics, biomarkers, and immunophenotypes associated with early death in a large European real-world cohort.

## Materials and methods

### Patient population

This study was initiated by the IMMUreal study group. The objective of the IMMUreal study group is to investigate the efficacy of immunotherapeutic agents for the treatment of liver tumors. Patient data were collected from six centers in Germany (University Hospital LMU Munich, University Hospital Würzburg, University Hospital Essen, Klinikum rechts der Isar TU Munich, University Hospital Magdeburg, and University Medical Center Mainz) and one center in Austria (Medical University of Vienna). All patients included in this study had a confirmed HCC diagnosis based on histopathological findings or characteristic diagnostic imaging according to the European Association for the Study of the Liver (EASL) criteria.^10^ Patients were divided into an EM cohort and a Non-EM cohort. The main analysis was conducted using a cut-off of 90 days for EM *vs.* Non-EM. Patients with insufficient follow-up <90 days were excluded to prevent underestimation of EM rates. We chose the 90-day cut-off because it represents a well-established threshold for mortality risk in hepatology and was used, for example, in the development of the model for end-stage liver disease (MELD) score.^11^ A confirmatory subgroup analysis divided patients into EM and Non-EM based on a 60-day cut-off, with patients with insufficient follow-up <60 days being excluded. The cut-off of 60-day mortality was chosen since it is an accepted parameter for evaluating the safety of systemic therapies for gastrointestinal cancers.^12^ As patients with Child–Pugh score (CPS) C and Barcelona Clinic liver cancer (BCLC) D are excluded from current recommendations and skewed the analysis because of the high risk of early death, these patients were excluded. To analyze the impact of clinical trial eligibility criteria, patients were grouped into those meeting IMbrave150 inclusion criteria (IMbrave150-IN) *vs.* patients not meeting the IMbrave150 criteria (IMbrave150-OUT). Patients were classified as IMbrave150-IN, if they met the following criteria: CPS A, Eastern Cooperative Oncology Group (ECOG) 0–1, no moderate or severe ascites, no history of hepatic encephalopathy (HE), no current or recent of full-dose anticoagulants for therapeutic purpose, no current or recent use of high-dose aspirin or other antiplatelets such as clopidogrel, prior variceal bleeding event within 6 months before start of atezo/bev, laboratory markers (albumin, bilirubin, creatinine, platelets, neutrophils, hemoglobin, international normalized ratio [INR]) within IMbrave150 eligibility criteria.^4^ The study was approved by local authorities and conducted in accordance with the Declaration of Helsinki. The Strengthening the Reporting of Observational Studies in Epidemiology (STROBE) checklist was used during manuscript preparation.^13^ Some patient data from some centers have been used in other projects.[14], [15], [16], [17], [18], [19]

### Ethics approval and consent

The study protocol was approved by the institutional review board (Ethics committees of the Medical Faculty of the Ludwig-Maximilians-Universität Munich [18-604, 20-439, 25-0223], University Medical Center of Mainz [837.199.10], Julius-Maximilians-Universität Würzburg [156/21-me], University Hospital Essen [21-71009-BO], Medical University of Vienna [2033/2017 and 1759/2015], Otto-von-Guericke University Magdeburg [70/22], and Klinikum rechts der Isar of the Technical University Munich [2022-605-S-KH]). Patient informed consent was obtained to perform biomarker analyses (20-439).

### Treatments and assessments

Patients received the following treatment regimen: atezo/bev, with atezolizumab being administered intravenously at a dose of 1,200 mg and bevacizumab at 15 mg per kg of body weight every 3 weeks. Monitoring of patients included clinical, laboratory, and imaging evaluations, aligning with the established standard of care per the German HCC guidelines.^20^ The assessment of tumor response was systematically conducted every 8–12 weeks through computed tomography and/or magnetic resonance imaging. Hepatic decompensation as death cause was defined following the statement by D’Amico *et al*.^1^ as new onset or worsening of ascites, variceal bleeding, and new onset or worsening of HE. Patients with pre-existing ascites were considered to have hepatic decompensation if ascites worsened during the observation period, necessitating paracentesis. Patients with pre-existing HE were considered to have hepatic decompensation if HE worsened as per Westhaven Criteria. Treatment-related adverse events were identified and treated following the guidance provided in the Summary of Product Characteristics and in the current guidelines, without application of predefined time cut-offs.^21^^,^^22^

### Endpoints

The primary endpoint of this study was to investigate the occurrence and risk factors of EM, defined as death of any cause within 3 months of therapy initiation. Secondary efficacy endpoints included overall survival, progression-free survival, and response rates. Overall survival (OS) was defined as the time from commencement of treatment until death from any cause. Progression-free survival (PFS) referred to the period from treatment initiation to the occurrence of disease progression on radiological assessment or death from any cause. Patients without recorded OS or PFS events or patients who were lost to follow-up were censored at the date of their most recent contact. Radiological response was characterized as complete or partial response (CR/PR), stable or progressive disease (SD/PD) by the local investigator. Overall response rate (ORR) was the proportion of patients experiencing CR and PR, while disease control rate was defined as CR plus PR plus SD.

### PBMC isolation and FACS analysis

Peripheral blood mononuclear cells (PBMCs) obtained before therapy initiation in one study center (EM: n = 8, Non-EM: n = 32) were isolated from patients’ EDTA-preserved whole blood by conventional Ficoll-Paque density gradient (Cytiva, Uppsala, Sweden) according to the manufacturer’s instruction and preserved in fetal calf serum (FCS)/10% DMSO at -150 °C until analyzed. After isolation, cells were resuspended in cryopreservation medium consisting of 90% FCS and 10% DMSO, cooled down to -80 °C in isopropanol-containing freezing containers for 1–3 days and then transferred to liquid nitrogen. For FACS analysis, PBMCs were thawed and rested in RPMI1640 medium containing 10% FCS, 1% penicillin/streptomycin and 1% l-glutamine for 1 h. Before antibody staining, cells were treated with Human TruStain FcX (BioLegend, San Diego, USA). Extracellular staining was performed with 1:200 dilution of antibodies and eBioscience™ Fixable Viability Dye eFluor™ 780 (Invitrogen, Waltham, USA). Upon permeabilization and fixation of cells with eBioscience™ Foxp3/Transcription Factor Staining Buffer Set (Invitrogen), cells were intracellularly stained with 1:100 diluted antibodies. Samples were measured on a BD LSRFortessa Cell Analyzer (BD Biosciences, Franklin Lakes, USA) and data were analyzed using FlowJo 10.8.1 (BD Biosciences). Staining and isotype antibodies (all purchased from Biolegend) with respective antigens, fluorophores and clones were used as shown in Box 1.

Box 1 **Antibodies for flow cytometry.**

**Table:** 

Antigen	Fluorophore	Clone
CD80	BV421	2D10
CD8	BV510	SK1
PD-L1	BV605	29E2A3
HLA-DR	BV650	L243
CD19	BV711	HIB19
CD11c	FITC	Bu15
CD4	PerCP-Cy5.5	SK3
CD86	PE	BU63
CD14	PE/Dazzle 594	HCD14
CD45RO	PE-Cy7	UCHL1
CCR7	APC	G043H7
CD3	AF700	OKT3
TIGIT	BV421	A15153G
ICOS	BV605	C398.4A
TIM-3	BV650	F38-2E2
CTLA4	BV711	BNI3
CD44	FITC	C44Mab-5
FOXP3	PE	259D
LAG-3	PE/Dazzle 594	11C3C65
Granzyme B	PE-Cy7	QA16A02
CD25	APC	M-T271
Mouse IgG1	BV421	MOPC-21
Mouse IgG1	BV510	MOPC-21
Mouse IgG2b	BV605	MOPC-11
Mouse IgG1	BV650	MOPC-21
Mouse IgG1	BV711	MOPC-21
Mouse IgG1	FITC	MOPC-21
Mouse IgG1	PerCP-Cy5.5	MOPC-21
Mouse IgG1	PE	MOPC-21
Mouse IgG1	PE/Dazzle 94	MOPC-21
Mouse IgG2a	PE-Cy7	MOPC-173
Mouse IgG2a	APC	MOPC-173
Mouse IgG1	APC	MOPC-21
Mouse IgG2a	AF700	MOPC-173
Mouse IgG2a	BV421	MOPC-173
Armenian Hamster IgG	BV605	HTK888
Mouse IgG2a	BV711	MOPC-173
Mouse IgG1	PE-Cy7	MOPC-21

Alt-text: Box 1

### Statistical analysis

Statistical evaluation was conducted utilizing GraphPad Prism 9 (GraphPad Software, San Diego, CA, USA). Patients characteristics were summarized using descriptive statistical methods. The conformity of variables to a normal distribution was examined using the Shapiro–Wilk test alongside qq-plot inspection. Continuous data were expressed as the mean with standard deviation and analyzed via the *t* test or Mann–Whitney *U* test based on their distribution characteristics. Categorical data were shown as frequencies and proportions. The comparison of categorical variables employed Fishers exact test. The Kaplan–Meier method was applied to estimate the median OS (mOS), with the log-rank test used for comparison. Odds ratios (ORs) for events were determined using both univariate and multivariate logistic regression analyses. To address collinearity between some continuous variables (hemoglobin, platelets) and the intercept, we centered the variables by subtracting the mean from each value. For multiple logistic regression, we selected the five variables with the highest ORs from the significant univariate results, reflecting the strongest associations with mortality. FACS data were analyzed using the Mann–Whitney *U* test. Values of *p* <0.05 were denoted as statistically significant.

## Results

### Baseline characteristics

**Fig. 1:**
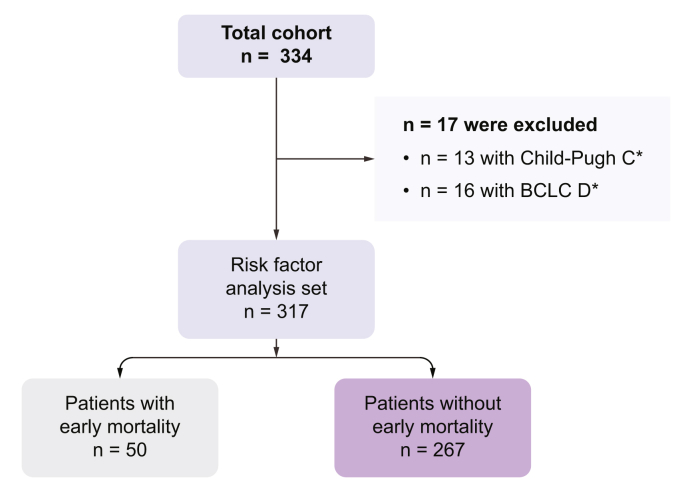
Study flow chart. ∗Cases can overlap. BCLC, Barcelona Clinic Liver Cancer Staging System.

A total of 317 patients with HCC treated with atezo/bev were included in the analysis set (Fig. 1). Demographics and HCC etiology were similar between the EM and Non-EM groups (Table 1). Underlying liver cirrhosis was present in the majority of cases (EM: n = 35/50 [70.0%] *vs.* Non-EM: n = 189/267 [70.8%] [*p* >0.99]), but liver function was notably poorer in patients with EM. This was evidenced by a significantly higher frequency of patients with CPS B (EM: n = 25/50 [50.0%] *vs.* Non-EM: n = 46/267 [17.2%] [*p* <0.001]). CPS A was more common in patients who were Non-EM (EM: n = 25/50 [50.0%] *vs.* Non-EM: n = 212/267 [79.1%] [*p* <0.001]). Accordingly, ALBI grade 1 (EM: n = 3/50 (6.0%) *vs.* Non-EM: n = 78/267 [29.2%] [*p* <0.001]) was more frequent in patients who were Non-EM, whereas ALBI grade 3 was more common in the EM cohort (EM: n = 22/50 [44.0%] *vs.* Non-EM: n = 22/267 [8.2%] [*p* <0.001]). ECOG performance status was worse in the EM group, with an ECOG ≥2 attributed to 16.3% of patients with EM *vs.* 7.1% of Non-EM (*p* = 0.048). In this cohort, 112 patients (35.3%) met IMbrave150 eligibility criteria. IMbrave150-IN status was significantly more common among patients who were Non-EM (n = 103/267 [38.6%]) as compared with patients with EM (n = 9/50 [18.0%], *p* = 0.006).

**Table 1:** Baseline characteristics.

	EM cohort n = 50	Non-EM cohort n = 267	*p* value
Variable			
Mean age ± standard deviation (years)	66.9 ± 11.5	67.82 ± 10.6	0.52
Female (%)	8 (16.0)	62 (23.2)	0.35
Etiology∗			
HBV	6 (12.0)	23 (8.6)	0.43
HCV	10 (20.0)	51 (19.1)	0.85
Alcohol-related	13 (26.0)	64 (24.0)	0.72
MASLD/MASH	5 (10.0)	56 (21.0)	0.08
Other	6 (12.0)	42 (15.7)	0.66
Unknown	13 (26.0)	48 (18.0)	0.24
Cirrhosis (%)	35 (70.0)	189 (70.8)	>0.99
Child–Pugh category (%)			
A	25 (50.0)	212 (79.4)	**<0.001**
B	25 (50.0)	46 (17.2)	**<0.001**
Missing	0 (0)	9 (3.4)	0.36
Child–Pugh score (%)			
A5	8 (16.0)	144 (53.9)	**<0.001**
A6	16 (32.0)	68 (25.5)	0.38
B7	11 (22.0)	28 (10.5)	**0.03**
B8	6 (12.0)	13 (4.9)	0.09
B9	8 (16.0)	5 (1.9)	**<0.001**
Missing	1 (2.0)	9 (3.4)	>0.99
Ascites (%)			
None	33 (66.0)	208 (77.9)	0.074
Moderate	12 (24.0)	53 (19.9)	0.56
Severe	4 (8.0)	6 (2.25)	0.056
Missing	1 (2.0)	0 (0)	0.16
Hepatic encephalopathy			
None	46 (92.0)	263 (98.5)	**0.024**
Mild	3 (6.0)	4 (1.5)	0.081
Severe	0 (0)	0 (0)	>0.99
Missing	1 (2.0)	0 (0)	0.16
ALBI grade			
1	3 (6.0)	78 (29.2)	**<0.001**
2	23 (46.0)	147 (55.1)	0.28
3	22 (44.0)	22 (8.2)	**<0.001**
Missing	2 (4.0)	20 (7.5)	0.55
ECOG PS ≥2 (%)†	8 (16.3)	19 (7.1)	**0.048**
BCLC stage (%)			
B	7 (14.0)	63 (23.6)	0.19
C	43 (86.0)	204 (76.4)	
Extrahepatic spread (%)^#^	22 (44.9)	116 (43.4)	0.88
Macrovascular invasion (%)^#^	27 (55.1)	106 (39.7)	0.06
Baseline gastroscopy (%)	46 (92.0)	244 (91.4)	>0.99
Gastroesophageal varices (%)	23 (46.0)	104 (39.0)	0.35
Esophageal varices grade (%)			
None	23 (46.0)	140 (52.4)	0.44
I	16 (32.0)	61 (22.8)	0.21
II	6 (12.0)	36 (13.5)	>0.99
III	1 (2.0)	6 (2.2)	>0.99
Missing	4 (8.0)	24 (9.0)	>0.99
Prior variceal bleeding (%)	5 (10.0)	10 (3.7)	0.07
NSBB (%)	15 (30.0)	72 (27.0)	0.73
Anticoagulation (%)	19 (38.0)	78 (29.2)	0.24
Antiplatelet drugs (%)	10 (20.0)	72 (27.0)	0.38
IMbrave150-IN (%)	9 (18.0)	103 (38.6)	**0.006**

Continuous variables were reported as mean plus standard deviation and compared via independent samples *t* test or Mann–Whitney *U* test, depending on the presence of a normal distribution. Categorical variables were reported as numbers and percentages. Comparisons of categorical variables were conducted by Fisher's exact test. A value of *p* <0.05 was considered statistically significant.

Bold font indicate significant *p* values.

∗ Several etiologies in one patient possible.

† In one patient, no data was available. BCLC, Barcelona Clinic Liver Cancer Staging System; ECOG, Eastern Cooperative Oncology Group performance status; EM, early mortality; HCC, hepatocellular carcinoma; IMbrave150-IN, patient meeting inclusion criteria for the IMbrave150 trial; MASH, metabolic dysfunction-associated steatohepatitis; MASLD, metabolic dysfunction-associated steatotic liver disease; NSBB, non-selective beta blockers.

### Risk factors for EM and causes of death

**Fig. 2:**
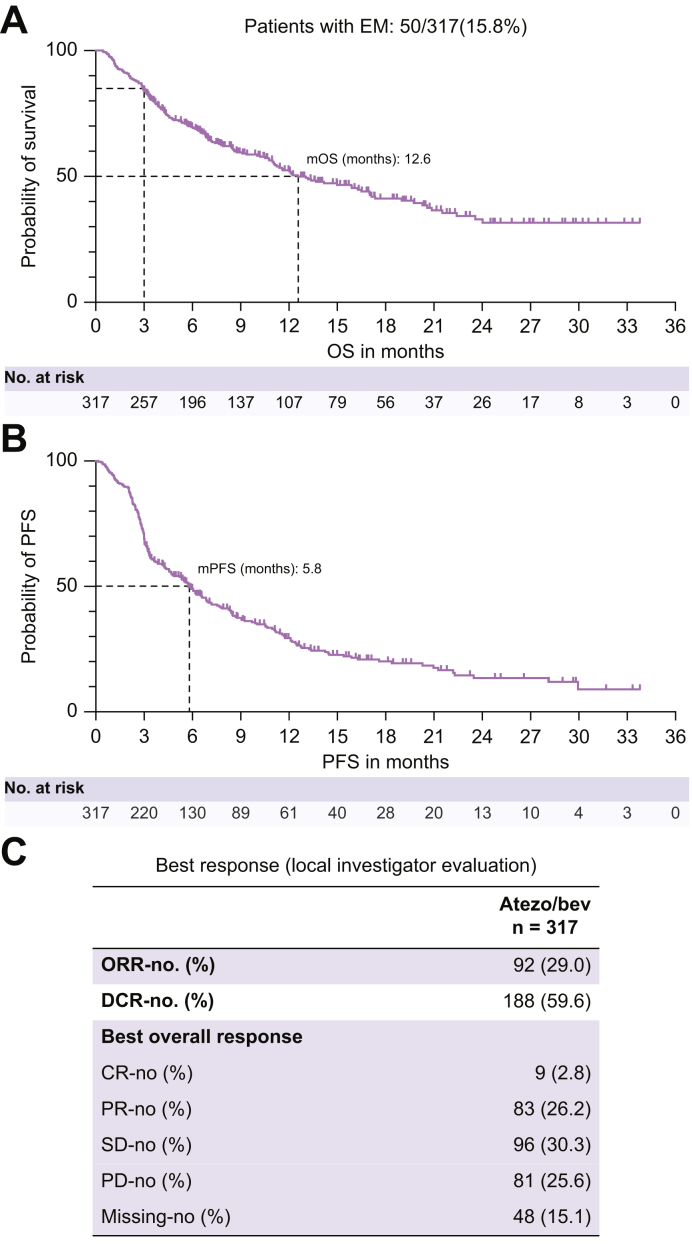
Efficacy analysis of atezolizumab/bevacizumab in all patients. (A) Median overall survival (mOS) with proportion of patients experiencing early mortality, (B) median progression-free survival, and (C) best response. The Kaplan–Meier method was applied to estimate the median survival. CR, complete response; DCR, disease control rate; EM, early mortality; mOS, median overall survival; ORR, objective response rate; mPFS, median progression-free survival; PD, progressive disease; PR, partial response.

EM, defined as death within the first 90 days of treatment, was observed in 15.8% of patients (n = 50/317). At a median follow-up of 15.5 months, the mOS and median PFS (mPFS) for the entire cohort were 12.6 months and 5.8 months, respectively (Fig. 2A and B). ORR was 29%, consisting of complete responses in nine patients (2.7%) and partial responses in 83 (26.2%) (Fig. 2C). SD was observed in 97 patients (30.6%) and PD in 82 (25.9%). Disease control could be achieved in 59.6%. mOS in patients who were CPS A and CPS B was 17.1 months and 4.2 months and mPFS 6.8 months and 3.2 months, respectively, which is similar to patient outcomes reported in the Imbrave150 trial^4^ and other real-world cohorts.^16^^,^^23^ Patients with EM had an mOS of 1.7 months (Fig. S1) with an mPFS of 1.2 months (Fig. S2) and an ORR of 2% (Table S5).

The primary objective was to assess the risk factors associated with EM in patients treated with atezo/bev (Table 2). Univariate regression analysis revealed that a poor performance status with an ECOG ≥2 significantly increased the risk of death within the first 90 days (OR 2.55, 95% CI 0.99–6.03, *p* = 0.039). Advanced cirrhosis, as evidenced by CPS B (OR 4.61, 95% CI 2.43–8.78, *p* <0.0001), was strongly associated with an increased risk of EM. High risk for early death in patients with reduced liver function was mirrored by significantly increased risk in patients with severe ascites (OR 4.2, 95% CI 1.03–15.51, *p* = 0.033). Whereas extrahepatic spread (OR 1.06, 95% CI 0.57–1.96, *p* = 0.851) did not affect the risk of early death, macrovascular invasion was significantly associated with EM (OR 1.86, 95% CI 1.01–3.47, *p* = 0.047). We further investigated laboratory values associated with EM in patients with HCC who were treated with atezo/bev. Low albumin (OR 0.14, 95% CI 0.07–0.25, *p* <0.0001) and high bilirubin levels (OR 2.06, 95% CI 1.5–2.94, *p* <0.0001) were significantly associated with increased risk of death within 90 days upon atezo/bev initiation. Additionally, C-reactive protein (CRP) levels were found to be significantly higher in patients experiencing EM (OR 1.13, 95% CI 1.06–1.21, *p* = 0.0006). Regarding blood count markers, increased numbers of white blood cells (OR 1.13, 95% CI 1.03–1.25, *p* = 0.012), lower hemoglobin (OR 0.81, 95% CI 0.7–0.94, *p* = 0.006) and higher platelets (OR 1.0, 95% CI 1.0–1.0, *p* = 0.006) were associated with EM. Alpha-fetoprotein (AFP) levels as surrogates of tumor aggressiveness were not linked to increased EM risk (OR 1.0, 95% CI 1.0–1.0, *p* = 0.133). Scores reflecting liver function were significantly associated with an increased risk of early death. Albumin–bilirubin (ALBI) score grade 2 (OR 4.07, 95% CI 1.36–17.52, *p* = 0.026) and ALBI grade 3 (OR 26.0, 95% CI 8.1–117.3, *p* <0.0001) strongly increased EM risk in our patient cohort. CPS A6 (OR 4.24, 95% CI 1.77–10.9, *p* = 0.0016), CPS B7 (OR 7.07, 95% CI 2.63–19.82, *p* = 0.0001), CPS B8 (OR 8.31, 95% CI 2.42–27.81, *p* = 0.0006) and CPS B9 (OR 28.8, 95% CI 7.94–116.9, *p* <0.001), were substantially associated with early death compared to CPS A5. Higher MELD scores observed in patients with EM were also linked to increased EM risk (OR 1.14, 95% CI 1.07–1.22, *p* <0.0001). Patients who would have been excluded from the IMbrave150 trial (IMbrave150-OUT), had a significantly higher risk of early death (OR 2.86, 95% CI 1.39–6.50, *p* = 0.0069).

**Table 2:** Univariable analysis of risk factors for early mortality in patients treated with atezo/bev.

Variable	Univariable odds ratio	95% CI	*p* value
Age	0.99	0.97–1.02	0.578
Sex (male)	1.59	0.74–3.81	0.262
ECOG PS ≥2	2.55	0.99–6.03	**0.039**
Liver cirrhosis	0.96	0.51–1.91	0.911
Non-viral etiology	0.72	0.38–1.43	0.341
CPS B (CPS A as reference)	4.61	2.43–8.78	**<0.0001**
CPS A6 (A5 as reference)	4.24	1.77–10.9	**0.0016**
CPS B7 (A5 as reference)	7.07	2.63–19.82	**0.0001**
CPS B8 (A5 as reference)	8.31	2.42–27.81	**0.0006**
CPS B9 (A5 as reference)	28.8	7.94–116.9	**<0.0001**
Ascites, moderate (no ascites as reference)	1.43	0.67–2.89	0.337
Ascites, severe (no ascites as reference)	4.2	1.03–15.51	**0.033**
Hepatic encephalopathy	4.29	0.82–20.06	0.062
BCLC C (A-B as reference)	1.9	0.86–4.8	0.139
Extrahepatic spread	1.06	0.57–1.96	0.851
Macrovascular invasion	1.86	1.01–3.47	**0.047**
Presence of GE varices	1.35	0.71–2.54	0.356
Esophageal varices grade I	1.6	0.78–3.22	0.194
Esophageal varices grade II	1.01	0.35–2.54	0.977
Esophageal varices grade III	1.01	0.05–6.32	0.99
History of variceal bleeding	2.86	0.86–8.44	0.066
Spleen size	1.09	0.98–1.22	0.108
NSBB	1.16	0.58–2.22	0.659
Anticoagulation	1.48	0.78–2.77	0.218
Antiplatelets	0.68	0.31–1.38	0.304
Albumin (g/dl)	0.14	0.07–0.25	**<0.0001**
Bilirubin (mg/dl)	2.06	1.5–2.94	**<0.0001**
ALBI Grade 2 (Grade 1 as reference)	4.07	1.36–17.52	**0.026**
ALBI Grade 3 (Grade 1 as reference)	26.0	8.1–117.3	**<0.0001**
INR	1.95	0.55–6.12	0.258
Creatinine (mg/dl)	1.26	0.82–1.98	0.243
Presence of proteinuria	0.97	0.38–2.25	0.941
CRP (mg/dl)	1.13	1.06–1.21	**0.0006**
AFP (ng/ml)	1.0	1.0–1.0	0.133
Leucocytes (10^9^/L)	1.13	1.03–1.25	0.012
Neutrophils (10^9^/L)	1.0	0.94–1.04	0.927
Platelets (10^9^/L)	1.0	1.0–1.0	**0.006**
Hemoglobin (g/L)	0.81	0.7–0.94	**0.006**
MELD score	1.14	1.07–1.22	**<0.0001**
Neutrophils to platelets ratio	1.0	0.99–1.0	0.869
IMbrave150-OUT (IMbrave150-IN as reference)	2.86	1.39–6.50	**0.0069**

Risk factors for early mortality in patients treated with atezo/bev. Odd’s ratio for early mortality risk was assessed by univariable logistic regression. *P* <0.05 was considered statistically significant. ALBI, Albumin–bilirubin; BCLC, Barcelona Clinic Liver Cancer; CI, confidence interval; CPS, Child–Pugh score; ECOG, Eastern Cooperative Oncology Group Performance Status; GE varices, gastoesophageal varices; IMbrave150-OUT, patient not meeting inclusion criteria for the IMbrave150 trial; MELD, model for end-stage liver disease; NSBB, non-selective beta blockers. Bold font indicate significant *p* values.

In multivariate regression analysis, reduced liver function parameters (CPS B: OR 3.96, 95% CI 1.95–8.01, *p* = 0.0001) and higher serum CRP (OR 1.09, 95% CI 1.01–1.18, *p* = 0.026) persisted as independent risk factors for EM (Table 3). As a 60-day cut-off for EM was also proposed for other systemic therapies,^12^ we additionally evaluated factors associated with increased risk of death within 60 days of atezo/bev initiation (Tables S1 and S2). Poor liver function and higher serum CRP values continued to represent an independent risk factor for early death in both univariate and multivariate regression models. We performed additional analyses for further risk stratification in patients with impaired and preserved liver function. Patients with poor performance status of ECOG ≥2 were enriched among patients with CPS B experiencing EM *vs.* patients with CPS A (Table S5). Among patients with CPS B, multivariate regression analysis revealed ECOG ≥2, high creatinine levels, and CPS B9 as independent predictors of early death (Tables S8 and S9). Among patients with preserved liver function with CPS A, ALBI grade provided a granular stratification of EM risk (Tables S6 and S7). ALBI grade 3 was strongly associated with EM in multivariate analysis (OR 12.54, *p* = 0.0123), while ALBI 2 was directionally consistent (OR 4.382) and borderline not significant (*p* = 0.056). Hemoglobin also emerged as an independent predictor of EM in this model (OR 0.7957, *p* = 0.0488).

**Table 3:** Multivariable analysis of risk factors for early mortality in patients treated with atezo/bev.

Variable	Multivariable odds ratio	95% CI	*p* value
CPS B (CPS A as reference)	3.956	1.952–8.068	**0.0001**
Macrovascular invasion	1.411	0.7025–2.844	0.3314
ECOG PS ≥2	1.452	0.4751–4.039	0.4904
Platelets (10^9^/L)	1.001	0.9983–1.004	0.4334
CRP (mg/dl)	1.090	1.010–1.181	**0.0258**

Risk factors for early mortality in patients treated with atezo/bev. Odd’s ratio for early mortality risk was assessed by multivariable logistic regression. *p* <0.05 was considered statistically significant. CPS, Child–Pugh score; ECOG PS, Eastern Cooperative Oncology Group Performance Status. Bold font indicate significant *p* values.

The main causes of early death among the 50 patients experiencing EM were HCC progression (n = 15, 29.4%), and hepatic decompensation (n = 14, 27.5%) (Fig. S3). Two patients (3.9%) died from bleeding, four (7.8%) from infection, three from cardiac events (5.9%), and two from other causes (n = 1 dermatologic event, n = 1 duodenal ulcer perforation). In one patient, two death causes were recorded: infection and HCC progression. Death cause remained unclear for 11 patients (21.6%). Two deaths were potentially related to atezolizumab (n = 1 dermatologic event, n = 1 myocarditis), five to bevacizumab (n = 1 variceal bleeding, n = 2 other bleeding, n = 1 cardiac event, n = 1 duodenal ulcer perforation), and one to both drugs (hepatic decompensation). Patients dying from hepatic decompensation exhibited higher frequencies of markers associated with impaired liver function at baseline, including elevated CPS, ALBI grades, a higher prevalence of HE, lower albumin and higher bilirubin levels (Table S3). In contrast, patients who died from tumor progression showed higher AFP values and a greater prevalence of extrahepatic spread. Both patients dying from hepatic decompensation and patients dying as a result of HCC progression showed a worsening of liver function parameters such as bilirubin and albumin. However, this was more pronounced for patients with hepatic decompensation as compared with those with HCC progression (Fig. S8). For other death causes, dynamic deterioration of liver function could not be observed.

### PBMC immunophenotyping

**Fig. 3:**
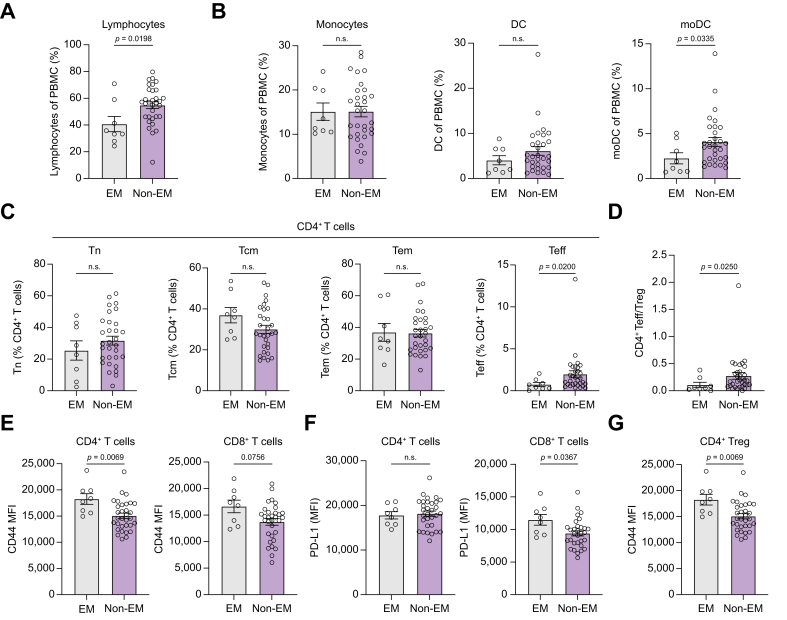
Immunophenotyping. PBMC of Atezo/bev-treated patients with HCC (EM: n = 8; Non-EM: n = 32) were analyzed via flow cytometry. (A, B) Frequencies of lymphocytes (A) and monocytes, DC and moDC (B) were determined. (C) Distribution of T cell phenotypes (Tn, naïve T cells; Tcm, central-memory T cells; Tem, effector-memory T cells; Teff, effector T cells) in CD4^+^ T cells was determined. (D) Ratio of CD4^+^ Teff to Treg was analyzed. (E, F) Expression of CD44 (E) and PD-L1 (F) on CD4^+^ and CD8^+^ T cells is displayed as mean fluorescent intensity (MFI). (G) CD44 MFI on CD4^+^ Treg is shown. Statistical analysis was performed using the Mann–Whitney *U* test. EM, early mortality; DC, dendritic cells; moDC, monocyte-derived DC; Treg, regulatory T cell; MFI, mean fluorescent intensity.

Since alterations in serum CRP levels in multivariate and the numbers of leukocytes in univariate analysis were significantly associated with EM, we aimed to comprehensively characterize the frequencies and functional status of immune cells before atezo/bev initiation to identify potential immunological biomarkers of EM risk. We performed in-depth immune phenotyping using FACS-based analysis of baseline PBMC in eight patients with EM and 32 who were Non-EM. Representative gating strategies are provided in Fig. S4. Baseline characteristics of our translational cohort were balanced regarding demographics, HCC etiology, and tumor-associated factors, whereas liver function was poorer in patients with EM compared with those who were Non-EM (Table S4). We observed significantly decreased frequencies of lymphocytes among PBMC in patients with EM compared with those who were Non-EM (Fig. 3A). Within the lymphocytic compartment, there was neither a significant difference in the abundance of T cells or B cells, nor a significant alteration in the ratio of cluster of differentiation (CD)4 and CD8 T cells, respectively, between EM and Non-EM groups (Fig. S5A). Regarding cells assigned to innate immunity, there was no significant difference in the frequencies of monocytes and pan dendritic cells (DCs), whereas the frequency of CD11c^+^CD14^+^ monocyte-derived DC (moDC) was significantly increased in the Non-EM group compared with the EM group (Fig. 3B). By analyzing the differentiation status of T cells in peripheral blood, we could demonstrate that CD4^+^ effector T cells (Teff) were significantly more prominent in patients who were Non-EM compared with those with EM (Fig. 3C). Additionally, the ratio of CD4^+^ Teff and regulatory T cells (Tregs) was significantly increased in the Non-EM compared with the EM cohort (Fig. 3D). There was no significant difference in the abundance of naïve T cells (Tn), central-memory T cells (Tcm) or effector-memory T cells (Tem) in CD4^+^ T cells as well as no difference in T cell phenotypes of CD8^+^ T cells between EM and Non-EM groups (Fig. 3C and Fig. S5B).

Besides determining the frequencies of diverse cell subpopulations, we also aimed to analyze the functional status of these cells in a comprehensive manner. Therefore, we stained T cells for the major activation markers CD25, CD44, Granzyme B (GrzB) and inducible T cell co-stimulator (ICOS) as well as for the inhibitory markers Cytotoxic T lymphocyte associated protein 4 (CTLA-4), Lymphocyte-activation gene 3 (LAG-3), T cell immunoreceptor with Ig and ITIM domains (TIGIT), T cell immunoglobulin and mucin domain-containing-3 (TIM-3) and Programmed death-ligand 1 (PD-L1). Overall, T cell functionality appeared similar in EM and Non-EM groups, as no significant differences could be detected in the expression of CD25, GrzB, ICOS, CTLA-4, LAG-3, TIGIT and TIM-3, neither in CD4^+^ nor in CD8^+^ T cells (Fig. S5C and D). However, we observed significantly increased expression levels of the activation marker CD44 on CD4^+^ T cells, and the immunosuppressive molecule PD-L1 on CD8^+^ T cells of patients with EM compared with those with Non-EM (Fig. 3E and F). In contrast, CD44 expression on CD8^+^ T cells and PD-L1 expression on CD4^+^ T cells was comparable in EM and Non-EM groups (Fig. 3E and F). Additionally, we could detect increased expression of CD44 on CD4^+^ Treg in patients with EM compared with patients who were Non-EM (Fig. 3G). Regarding the functionality of antigen-presenting cells, we analyzed B cells, DC, moDCs, and monocytes regarding their expression of the co-stimulatory markers CD80, CD86 and Human Leukocyte Antigen – DR isotype (HLA-DR) as well as the co-inhibitory molecule PD-L1. No significant alterations could be detected in the expression of functional markers in B cells (Fig. S5E), DCs (Fig. S5F), moDCs (Fig. S5G) and monocytes (Fig. S5H). Heat maps providing an overview of the immunophenotypes observed within our analysis are displayed in Fig. S6. To address the contribution of HCC etiology to the immunophenotypical changes associated with EM in our cohort, we re-analyzed all significant changes between EM and Non-EM in patients with non-viral HCC (Fig. S7). Seven patients with EM and 23 with Non-EM remained to be analyzed in this subgroup. Here, lymphocyte frequency was lower for EM (Fig. S7A). Additionally, the frequency of moDC was also increased for patients with non-viral HCC who were Non-EM (Fig. S7B). The expression of CD44 on CD4^+^ T cells and CD4^+^ Treg persisted to be significantly higher in patients with EM compared with Non-EM in this subgroup (Fig. S7E and G). PD-L1 expression levels, however, on CD4^+^ and CD8^+^ T cells were not associated with EM in patients with non-viral HCC (Fig. S7F).

**Fig. 4:**
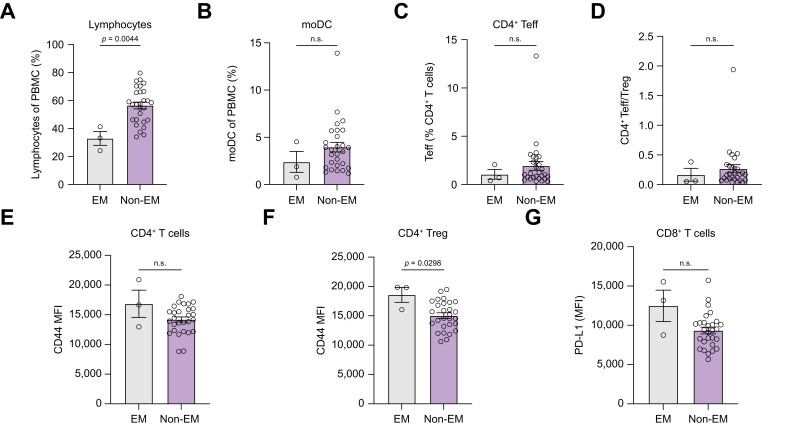
Immunophenotyping. Subgroup analysis PBMC of Atezo/bev-treated patients with HCC with preserved liver function according to CPS A (EM: n = 3; Non-EM: n = 28) were analyzed via flow cytometry. (A, B) Frequencies of lymphocytes (A) moDC (B) were determined. (C) Frequency of CD4^+^ effector T cells (Teff) among CD4^+^ T cells was determined. (D) Ratio of CD4^+^ Teff to Treg was analyzed. (E, F) Expression of CD44 on CD4^+^ T cells (E) and Treg (F) is displayed as mean fluorescent intensity (MFI). (G) PD-L1 MFI on CD8^+^ T cells is shown. Statistical analysis was performed using the Mann–Whitney *U* test. EM, early mortality; MFI, mean fluorescent intensity; moDC, monocyte-derived DC; Treg, regulatory T cell.

Furthermore, to exclude that alterations in immune cell composition and functional status were attributed to differences in liver function between patients with EM and Non-EM, we re-assessed all significant changes in patients with EM and Non-EM with preserved liver function, as evidenced by CPS A. From the whole biomarker analysis cohort, three patients with EM and 28 patients with Non-EM remained to be analyzed in this subgroup analysis. Here, lymphocyte frequency persisted to be significantly decreased in patients with EM compared with Non-EM (Fig. 4A). Frequencies of moDC and CD4^+^ Teff, and the CD4^+^ Teff/Treg ratio differed not statistically significantly between patients with EM and Non-EM when we adjusted for liver function (Fig. 4B–D). Additionally, differences in CD44 expression on CD4^+^ T cells between patients with EM and Non-EM also lost statistical significance in this subgroup analysis (Fig. 4E). However, increased CD44 expression on Treg remained a significant biomarker of EM, also when adjusted to liver function (Fig. 4F). In contrast, increased PD-L1 expression on CD8^+^ T cells of patients with EM was not statistically significant in CPS A patients (Fig. 4G).

## Discussion

This large, multicenter cohort study evidences an important risk for EM in patients with unresectable HCC treated with atezo/bev in a real-world setting. Significant risk factors for EM involved impaired liver function and alterations in systemic immunity, but not reduced performance status, age, or characteristics of advanced cancer such as extrahepatic spread, macrovascular invasion, and elevated AFP.

This work is the first to explicitly report EM rates for patients with HCC treated with atezo/bev. The 90-day mortality rate was 15.8% (50/317), considerably higher than the ∼5% rate seen in the Imbrave150 trial.^4^^,^^24^ Hepatic decompensation and HCC progression represented the main death causes in our cohort. In contrast, treatment-related toxicity was not identified as major cause of EM. The discrepancy of EM rates reported in our real-world study and the IMbrave150 trial probably reflects the stringent trial eligibility criteria, which do not fully represent the typical patient population in routine clinical practice. In this context, a large proportion of patients in this large, European cohort (64.7%) did not meet the eligibility criteria for the IMbrave150 trial, emphasizing the need for high-quality real-world data to bridge the gap between clinical trials and clinical practice to inform clinical decision-making.

The strongest predictor of 90-day mortality was advanced liver disease in univariate analysis, as reflected by surrogates of impaired liver function such as CPS B (*p* <0.0001), ALBI grade 2/3 (*p* = 0.026, *p* <0.0001), high MELD score (*p* <0.0001), high bilirubin (*p* <0.0001), and low albumin (*p* = 0.003). CPS B remained significant risk factors after adjusting for other variables (*p* = 0.0001). These factors were also significantly associated with the risk of death within the first 60 days of atezo/bev therapy (CPS B: *p* <0.001). Although ECOG was associated with EM in univariate analysis, it did not retain significance in the multivariate model. This suggests that the influence of performance status on EM is mediated by other factors included in the multivariate analysis, especially liver function and CRP and does not independently distinguish between EM and Non-EM.

The treatment of patients with HCC and reduced liver function is a daily challenge to hepato-oncologists. About one-third of patients with HCC patients have moderate-to-severe liver disease.[25], [26], [27], [28] Impaired liver function often limits the use of local or locoregional therapies,[29], [30], [31] and advanced tumor characteristics alongside organ shortage preclude many from liver transplantation, which represents a cure for both cancer and cirrhosis. All currently approved systemic therapies for HCC were exclusively studied in patients with CPS A in the pivotal trials.^3^ Faced with this therapeutic dilemma, palliative care is frequently the only option for those with advanced cancer and impaired liver function, yielding a median survival of 4 months.^32^^,^^33^ The remarkable oncological efficacy of ICI-based therapies such as atezo/bev in HCC has raised hopes as to whether the benefit derived from ICI could extend to patients with impaired liver function. Our study clearly shows that impaired liver function significantly increases the risk of dying within the first 3 months of atezo/bev treatment, with an OR of 3.96 for CPS B (*p* = 0.0001) in multivariable analysis. Among CPS B patients, those with CPS B9, a poor performance status of ECOG ≥2 and high creatinine levels had a particularly high risk of early death. These variables were not significant predictors of EM in individuals with CPS A, where ALBI grade 3 emerged as a strong predictor of early death. These findings emphasize the need for refined risk stratification approaches within patients with impaired liver function.

Limited oncological outcomes in patients with CPS B treated with atezo/bev were shown in previous retrospective studies^14^^,^^16^^,^^17^^,^^23^^,^[34], [35], [36] and summarized in a recent meta-analysis.^5^ mOS was 6.8 months, which is considerably lower compared with 19.4 months reported in the Imbrave150 trial.^4^^,^^24^ Patients with CPS B suffered from more treatment-related toxicity with a higher rate of grade ≥3 adverse events of 26.8% *vs.* 11.8% in CPS A. Our findings underscore the necessity of maintaining and stabilizing liver function in patients with HCC to reduce EM risk upon atezo/bev treatment. Early transition from locoregional to systemic treatment could mitigate the collateral damage from repeated transarterial chemoembolization or radioembolization.^10^^,^^37^ Successful management of underlying etiology in liver cirrhosis through sustained abstinence in alcohol-related liver diseases and cure or long-term suppression in chronic hepatitis B/C infection can lead to the resolution of hepatic decompensation.[38], [39], [40], [41] Atezo/bev therapy can be safely administered to patients with active viral hepatitis, and viral clearance is not necessary before initiating ICI therapy in the advanced setting.^42^^,^^43^ However, studies have shown that effective antiviral therapy is associated with a reduced risk of decompensation in patients with HCC treated with atezo/bev, suggesting a protective effect.^44^

Inflammatory conditions and immune dysbalance are frequent in cancer and can promote tumor progression, invasion, metastasis, and angiogenesis.^45^ In the context of HCC, the complex composition of the tumor immune microenvironment as well as the abundance of certain immune cell subsets in blood and ascites have been linked to patient prognosis and response to immunotherapy.[46], [47], [48], [49] Patients experiencing EM had a significantly higher CRP in multivariate analysis (*p* = 0.026). Inflammatory cytokines such as CRP can suppress antitumor immunity^50^ and high CRP is a predictor for worse oncological outcomes in patients with HCC treated with immunotherapy.^19^ To address immune alterations mediated by inflammatory conditions, we performed immunophenotyping of baseline patients’ PBMCs, which revealed decreased frequencies of lymphocytes and CD4^+^ Teff, reduced ratio of CD4^+^ Teff to Treg, and increased expression of the activation marker CD44 on particularly Treg to be significantly associated with EM. Subgroup analysis in patients with preserved liver function revealed that these differences were largely driven by poor liver function. Interestingly, these differences are also associated with decreased antitumor response of ICI therapy. In this context, lower numbers of peripheral blood lymphocytes have been associated with poor outcomes in cancer patients treated with immune checkpoint inhibitors.[51], [52], [53] Additionally, regarding Treg, increased CD44 expression has been attributed to enhanced suppressive functionality and persistence.^54^^,^^55^ Although the frequency of intratumoral and peripheral Treg has already been linked to patient prognosis, the impact of Treg functionality on the prognosis of patients during checkpoint-inhibitor therapy is not well understood to date. However, high CD44 Treg expression representing a highly immunosuppressive Treg phenotype might also represent a detrimental factor for checkpoint-inhibitor response. Additionally, we observed enhanced expression of the immunosuppressive molecule PD-L1 on CD8^+^ T cells of patients with EM, which has also been linked suppressive function of CD8^+^ T cells, and to resistance to checkpoint inhibitors.^56^^,^^57^ Altogether, we conclude that outcomes for patients with HCC with impaired liver function may be constrained both by (1) their increased independent risk of early death due to hepatic decompensation, and (2) an immune profile less capable of mounting a robust anticancer response upon ICI therapy.

This study has limitations. Because of its retrospective nature, some clinical and laboratory parameters were missing in small subsets of patients (Table 1). Second, patients were recruited from tertiary hospitals with high expertise in the management of HCC, which might underestimate the rate of EM. Nevertheless, the extensive sample size of this study allowed for a thorough multivariable regression analysis to identify clinical and biochemical EM risk factors. Third, the subgroup analysis of immunophenotyping in patients with preserved liver function had a limited sample size with three patients experiencing EM. Larger studies are needed to investigate the influence of circulating PBMCs on mortality in patients with preserved liver function. Fourth, classification of death causes was done without autopsy based on the available clinical, laboratory, and imaging data, which might have led to misclassifications.

In summary, this large multicenter study is the first to draw attention to early death as a significant and frequent outcome for patients treated with atezo/bev in the real world. The identified risk factors can help clinicians identify patients at high risk of EM, enabling timely and informed decision-making regarding end-of-life care and palliative treatment. Addressing these issues proactively is a fundamental responsibility of the treating physician.

### Conclusions

EM is frequent in patients with HCC treated with atezo/bev. Significant risk factors for dying early involved impaired liver function, elevated biomarkers of inflammation, and alterations of systemic immunity, but not reduced performance status, age, or characteristics of advanced cancer such as extrahepatic spread, macrovascular invasion, or elevated AFP.

## Abbreviations

AFP, alpha-fetoprotein; ALBI, albumin–bilirubin; Atezo/bev, atezolizumab plus bevacizumab; BCLC, Barcelona Clinic liver cancer; CD, cluster of differentiation; CPS, Child–Pugh score; CR, complete response; CRP, C-reactive protein; CTLA-4, Cytotoxic T lymphocyte associated protein 4; DC, dendritic cell; EASL, European Association for the Study of the Liver; ECOG, Eastern Cooperative Oncology Group; EM, early mortality; FCS, fetal calf serum; GrzB, granzyme B; HCC, hepatocellular carcinoma; HE, hepatic encephalopathy; HLA-DR, human leukocyte antigen – DR isotype; ICI, immune checkpoint inhibitor; ICOS, inducible T cell co-stimulator; INR, international normalized ratio; LAG-3, Lymphocyte-activation gene 3; MELD, model for end-stage liver disease; moDC, monocyte-derived dendritic cells; mOS, median overall survival; mPFS, median progression-free survival; NSBB, non-selective beta-blocker; OR, odds ratio; ORR, overall response rate; OS, overall survival; PBMC, peripheral blood mononuclear cell; PD, progressive disease; PD-L1, Programmed death-ligand 1; PFS, progression-free survival; PR, partial response; SD, stable disease; Tcm, central-memory T cell; Teff, effector T cell; Tem, effector-memory T cell; TIGIT, T cell immunoreceptor with Ig and ITIM domains; TIM-3, T cell immunoglobulin and mucin domain-containing-3; Tn, naïve T cell; Treg, regulatory T cell; VEGF, vascular endothelial growth factor.

## Financial support

This study was initiated by the IMMUreal study group. The protocol was endorsed and supported by the German 10.13039/100027925Alliance for Liver Cancer (GALC). NBK was supported by the Bavarian Cancer Research Center, FöFoLe of 10.13039/501100005722LMU Munich funding program (1122), the ESMO Research Fellowship, and the 10.13039/501100012353German Cancer Consortium. IP was supported by the Else Kröner-Fresenius-Stiftung (2025_EKEA.16 and IOLIN), the Bavarian Cancer Research Center, the Medical Faculty of the 10.13039/501100005722LMU Munich (intramural funding) and the 10.13039/100008273Novartis Foundation (InCa prize).The funding bodies had no role in the design of the study; the collection, analysis, or interpretation of data; or the writing of the manuscript.

## Authors’ contributions

Conceptualization: IP, ENDT, SJG, AG, FPR, NBK. Data curation: all authors. Methodology: NBK, FPR, IP, OÖ, SJG, MA-F. Project administration: NBK, FPR, IP. Resources: NBK, FPR, IP, AP, DA, MA-F, JR, JM, AG, ENDT. Software: NBK, FPR, IP. Supervision: DA, KBe, CS, CML, JR, JM, AG, ENDT. Writing – original draft: IP, NBK, FPR, ENDT, SE. Writing – review and editing: all authors.

## Data availability

All data being analyzed as part of this study are included in this manuscript and the supplementary materials. Further inquiries can be sent to the corresponding author.

## Conflicts of interest

IP received reimbursement of travel expenses from Roche. NBK has received reimbursement of meeting attendance fees and travel expenses from Eisai and lecture honorarium from Falk and Astra Zeneca. FPR has received honoraria for lectures and travel support from the Falk Foundation, Gilead, Ipsen and Novartis. AP has received honoraria for lectures from the Falk Foundation, and travel support from Roche. UE has received honoraria for lectures from AstraZeneca, the Falk Foundation, IPSEN, Novartis and Roche, and travel support from AstraZeneca and Biotest. She has served as advisory board or steering committee member to AstraZeneca, Bayer, Eisai, and MSD. KBo has received honoraria for lectures from IPSEN. KBe received a research grant from Roche Pharma AG. MP served as a speaker and/or consultant and/or advisory board member for Astra Zeneca, Bayer, Bristol-Myers Squibb, EISAI, Ipsen, Lilly, MSD, and Roche, and received travel support from Bayer, Bristol-Myers Squibb, Ipsen, and Roche. BS received grant support from AstraZeneca and EISAI, speaker honoraria from EISAI as well as travel support from AbbVie, AstraZeneca, Ipsen and Gilead. MR has received honoraria for lectures and travel support from Gilead. OÖ received honorarium from Bayer. MV has received honoraria for speaker, consultancy, and advisory role from Amgen, AstraZeneca, Bayer, BMS, Eisai, Ipsen, Lilly, Merck Serono, MSD, Nordic Pharma, Roche, Servier and Sirtex. SJG has received travel expenses from Gilead and Ipsen. LSJ has received honoraria for lectures from AstraZeneca, the Falk Foundation, AbbVie and Boston scientific and travel support from Biotest and AbbVie. She has served as advisory board member to AstraZeneca. FF has received honoraria for lectures from AstraZeneca, MSD, Pfizer, Roche and reimbursement of meeting attendance fees and travel expenses from Merck KGaA and Servier. He has served as advisory board or steering committee member to AstraZeneca, BMS, EISAI and Roche. AG is advisory board or steering committee member to AbbVie, Advanz, Albireo, Alexion, AstraZeneca, Bayer, Boehringer, BMS, EISAI, Falk, Gilead, Heel, Intercept, Ipsen, Merz, MSD, Novartis, Pfizer, Roche, Sanofi-Aventis and speaker for AbbVie, Adavanz, Alexion, BMS, Burgerstein, CSL Behring, Falk, Gilead, Intercept, Merz, MSD, Novartis, Novo Nordisk, Orphalan, Roche; he received research support from Intercept and Falk (NAFLD CSG), Novartis. ENDT has served as a paid consultant for AstraZeneca, Bayer, BMS, Eisai, Eli Lilly & Co, Pfizer, IPSEN, and Roche. He has received reimbursement of meeting attendance fees and travel expenses from Arqule, AstraZeneca, BMS, Bayer, Celsion, and Roche, and lecture honoraria from BMS and Falk. In addition, he has received third-party funding for scientific research from Arqule, AstraZeneca, BMS, Bayer, Eli Lilly, and Roche. AT served as consultant for Astellas Gene Therapies Inc. and Roche Pharma AG. JR received grants and personal fees from Sirtex and Bayer. All the other authors have no conflicts of interest to declare.

Please refer to the accompanying ICMJE disclosure forms for further details.
