# Supplementary material for: Early mortality in atezolizumab/bevacizumab for HCC is associated with impaired liver function and alterations of systemic immunity
Source: JHEP Rep. 2025 Jul 5;7(11):101513. doi: 10.1016/j.jhepr.2025.101513 (PMC12861967; doi:10.1016/j.jhepr.2025.101513)
Supplement: Multimedia component 1 [file mmc1.pdf]

Early mortality in atezolizumab/bevacizumab for HCC is associated with impaired liver function and alterations of systemic immunity

Ignazio Piseddu, Leonie S. Jochheim, Katrin Boettcher, Bernhard Scheiner, Friedrich Sinner, Simon Johannes Gairing, Matthias Thaler, Stefan Enssle, Monika Karin, Valentina Zarka, Alexander Philipp, Andreas Thalmeier, Jan Gaertig, Lorenz Balcar, Julia Martina Schütte, Julia S. Schneider, Katarina Ondrejkova, Monika Rau, Alexander Weich, David Anz, Karin Berger, Christian Schulz, Christian M. Lange, Osman Öcal, Marianna Alunni-Fabbroni, Jens Ricke, Ursula Ehmer, Marino Venerito, Friedrich Foerster, Matthias Pinter, Andreas Geier, Julia Mayerle, Enrico N. De Toni, Florian P. Reiter, Najib Ben Khaled

Table of contents

Supplementary figures……………………………………………………………….2

Supplementary tables……………………………………………………………….10

Supplementary figures

Fig. S1. Median overall survival of the early mortality cohort.

Fig. S2. Median progression-free survival of the early mortality cohort.

Fig. S3. Causes of death in the early mortality cohort. Diseases were categorized as liver decompensation, HCC progression, bleeding, infection, cardiac event, thromboembolic event, respiratory event, neurologic event, endocrine toxicity, renal toxicity, other toxicity, or unknown cause. Reasonable causal relationship to atezolizumab, bevacizumab, or both drugs was assessed by the local investigator. In one patient, two death causes were recorded: infection and HCC progression.

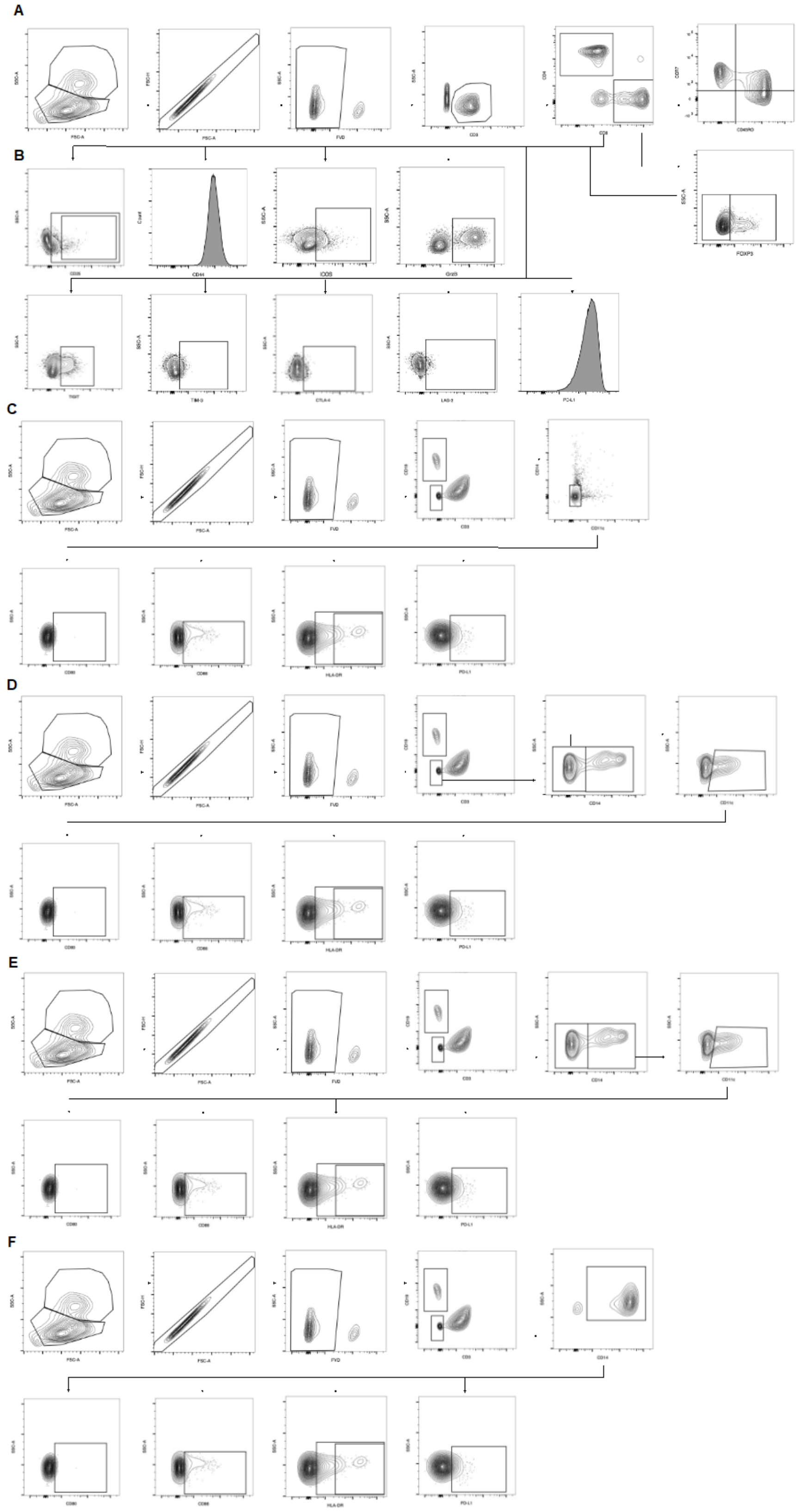

Fig. S4. Gating strategies. Representative gating strategies for (A) T cell phenotype, (B) T cell activation and exhaustion, (C) B cell activity, (D) DC activity, (E) moDC activity and (F) monocyte activity are shown.

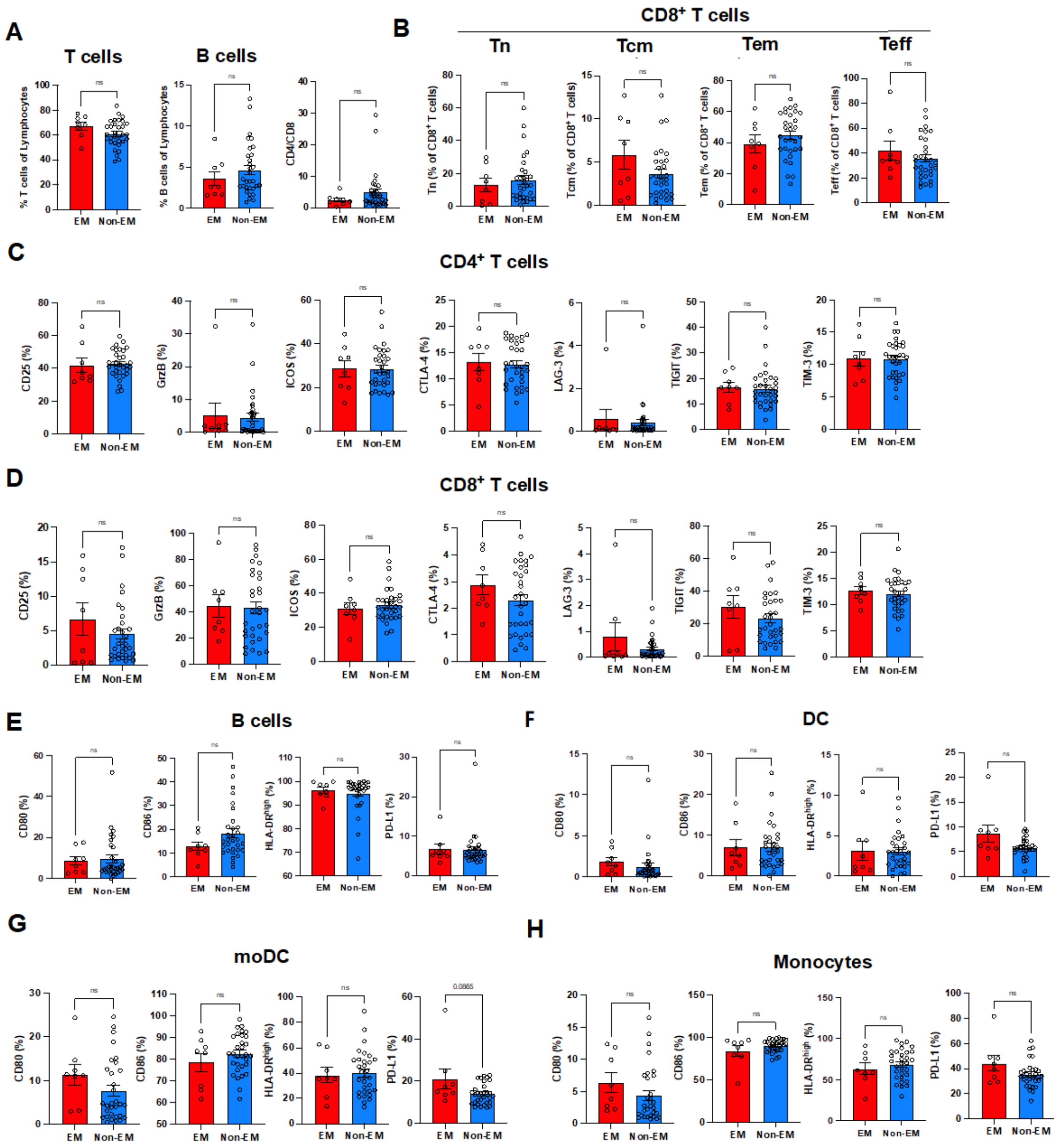

Fig. S5. Immunophenotyping. PBMC of Atezo/bev-treated HCC patients (EM: n = 8; Non-EM: n = 32) were analysed via flow cytometry. (A) Frequencies of CD4+ and CD8+ T cells within T cells as well as CD4 to CD8 ratio are shown. (B) Distribution of T cell phenotypes (Tn = naive T cells, Tcm = central memory T cells, Tem= effector memory T cells, Teff= effector T cells) in CD8+ T cells was determined. (C + D) Expression of the activation markers CD25, Granzyme B (GrzB) and ICOS as well as the exhaustion markers CTLA-4, LAG-3, TIGIT and TIM-3 are displayed in CD4+ (C) and CD8+ T cells (D). (E-H) Expression of the activation markers CD80, CD86 and HLA-DR as well as the inhibitory molecule PD-L1 were determined on (E) B cells, (F) DC, (G) moDC and (H) monocytes. Abbreviations: EM, early mortality; GrzB, Granzyme B; DC, dendritic cells; moDC, monocyte-derived DC.

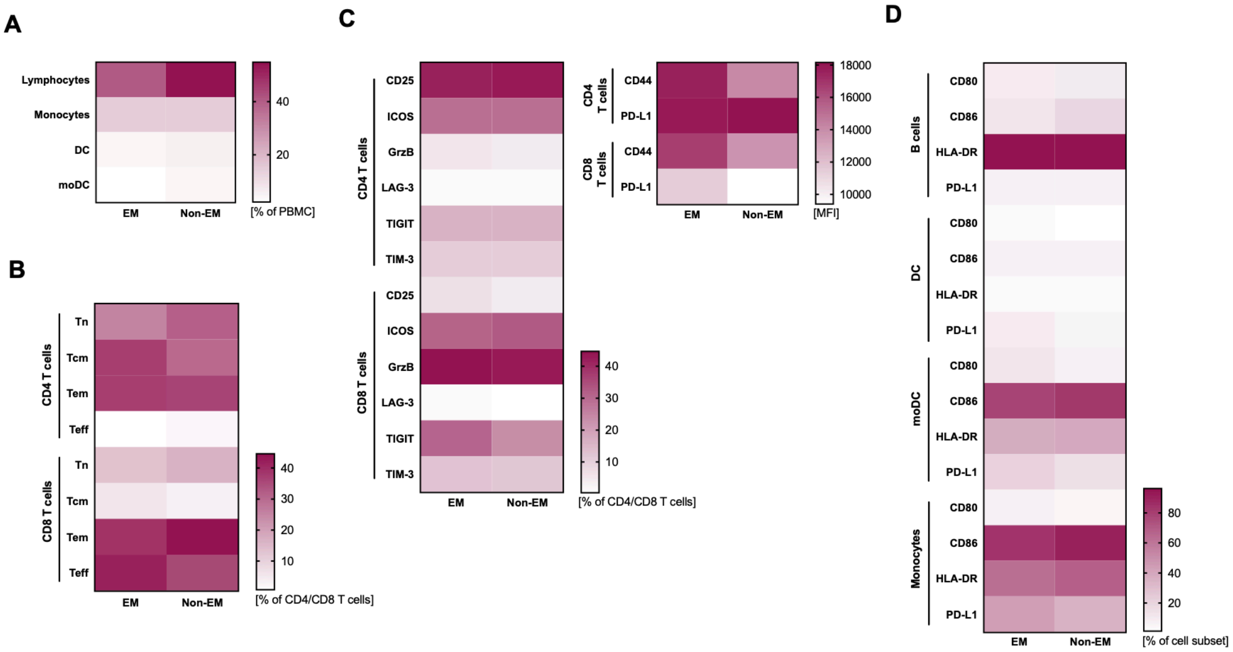

Fig. S6. Overview heat maps of PBMC immunophenotyping. PBMC of Atezo/bev-treated HCC patients (EM: n = 8; Non-EM: n = 32) were analysed via flow cytometry. (A) Frequencies of indicated immune cell subsets among whole PBMC are shown. (B) Distribution of T cell phenotypes (Tn = naive T cells, Tcm = central memory T cells, Tem= effector memory T cells, Teff= effector T cells) in CD4+ and CD8+ T cells is displayed. (C) Expression of the activation markers CD25, Granzyme B (GrzB) and ICOS as well as the exhaustion markers CTLA-4, LAG-3, TIGIT and TIM-3 are displayed in CD4+ and CD8+ T cells (left). Mean fluorescence intensity (MFI) of CD44 and PD-L1 on CD4+ and CD8+ T cells is shown (right). (D) Expression of the activation markers CD80, CD86 and HLA-DR as well as the inhibitory molecule PD-L1 were determined on B cells, DC, moDC and monocytes. Abbreviations: EM, early mortality; GrzB, Granzyme B; DC, dendritic cells; moDC, monocyte-derived DC.

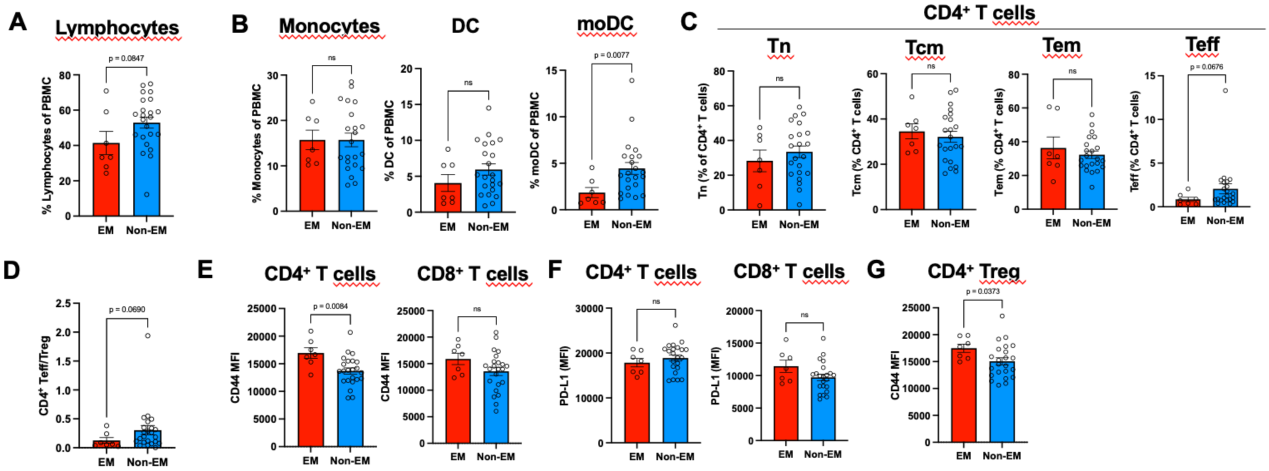

Fig. S7. PBMC of Atezo/bev-treated patients with non-viral HCC (EM: n = 7; Non-EM: n = 23) were analyzed via flow cytometry. (A + B) Frequencies of lymphocytes (A) and monocytes, DC and moDC (B) were determined. (C) Distribution of T cell phenotypes (Tn = naive T cells, Tcm = central memory T cells, Tem= effector memory T cells, Teff= effector T cells) in CD4+ T cells was determined. (D) Ratio of CD4+ Teff to Treg was analyzed. (E + F) Expression of CD44 (E) and PD-L1 (F) on CD4+ and CD8+ T cells is displayed as mean fluorescent intensity (MFI). (G) CD44 MFI on CD4+ Treg is shown. Abbreviations: EM, early mortality; DC, dendritic cells; moDC, monocyte-derived DC; Treg, regulatory T cell; MFI, mean fluorescent intensity

Fig. S8. Changes in liver function from baseline to follow-up visit in patients with HCC treated with atezo/bev and experiencing early mortality due to (A) progressive cancer, (B) hepatic decompensation, or (C) other causes. Baseline consists in the first cycle of atezo/bev. For follow-up values, values from week 6 after treatment start were used, where available. If no week 6 values were available, we included the closest value between week 3 and week 12. Scatter plots depict individual values and mean. Paired t-test was used to compare baseline and follow-up. Abbreviations: ALBI, Albumin-Bilirubin.

Supplementary tables

Table S1. Univariate analysis of risk factors for 60 days early mortality in patients treated with atezo/bev

**Variable**	**Univariate Odd's ratio**	**95% (CI)**	**p-value**
Age	1.01	0.97 to 1.05	0.603
Sex (male)	2.63	0.89 to 11.28	0.121
ECOG PS ≥ 2	3.24	1.1 to 8.4	**0.021**
Liver cirrhosis	0.76	0.35 to 1.78	0.516
Nonviral etiology	0.62	0.27 to 1.48	0.264
CPS B (CPS A as reference)	6.09	2.77 to 13.79	**<0.001**
CPS A6 (A5 as reference)	9.18	2.3 to 61.17	**0.005**
CPS B7 (A5 as reference)	16.95	3.89 to 117.4	**0.001**
CPS B8 (A5 as reference)	27.68	5.44 to 206.1	**0.002**
CPS B9 (A5 as reference)	48.44	9.01 to 378.3	**<0.001**
Ascites, moderate (no ascites as reference)	1.48	0.56 to 3.58	0.399
Ascites, severe (no ascites as reference)	5.45	1.1 to 21.53	**0.02**
Hepatic encephalopathy	1.79	0.09 to 11.02	0.596
BCLC C (A-B as reference)	4.1	1.19 to 25.83	0.058
Extrahepatic spread	1.51	0.7 to 3.34	0.295
Macrovascular invasion	2.3	1.05 to 5.22	**0.04**
Presence of GE varices	1.31	0.6 to 2.87	0.5
Esophageal varices grade II-III (0-I as reference)	0.81	0.23 to 2.21	0.705
History of variceal bleeding	2.73	0.6 to 9.28	0.138
Spleen size	1.05	0.91 to 1.21	0.486
NSBB	0.82	0.31 to 1.9	0.654
Anticoagulation	1.24	0.54 to 2.73	0.595
Antiplatelets	1.75	0.7 to 5.33	0.273
Albumin g/dl	0.1	0.04 to 0.22	**<0.001**
Bilirubin md/gl	2.26	1.58 to 3.36	**<0.001**
ALBI Grade 2 (Grade 1 as reference)	5.12	0.96 to 94.76	0.122
ALBI Grade 3 (Grade 1 as reference)	48	9.16 to 885.6	**<0.001**
INR	2.31	0.5 to 8.05	0.2127
Creatinine mg/dl	1.11	0.54 to 1.73	0.673
Presence of proteinuria	0.76	0.21 to 2.27	0.65
CRP mg/dl	1.16	1.07 to 1.25	**0.002**
AFP ng/ml	1	1.0 to 1.0	0.952

Leucocytes 10^9/L	1.16	1.03 to 1.3	**0.01**
Neutrophils 10^9/L	1.01	0.95 to 1.06	0.533
Platelets 10^9/L	1	1.0 to 1.01	**0.002**
Hemoglobin g/dl	0.83	0.69 to 1.0	**0.046**
MELD score	1.13	1.05 to 1.22	**0.001**
Neutrophils to platelets ratio	1	0.99 to 1.01	0.944

Risk factors for 60 days early mortality in patients treated with atezo/bev. Odd's ratio for early mortality risk was assessed by univariate logistic regression. Abbreviations: ALBI, Albumin-Bilirubin; BCLC, Barcelona Clinic Liver Cancer; CI, confidence interval; CPS, Child-Pugh score; ECOG, Eastern Cooperative Oncology Group Performance Status; GE varices, gastoesophageal varices; MELD, Model of Endstage Liver Disease; NSBB, non-selective beta-blockers.

Table S2. Multivariate analysis of risk factors for 60 days early mortality in patients treated with atezo/bev

**Variable**	**Univariate Odd's ratio**	**95% (CI)**	**p-value**
CPS B (CPS A as reference)	6.794	2.811 to 17.46	**<0.0001**
CRP mg/dl	1.121	1.031 to 1.225	**0.0065**
Platelets 10^9/L	1.001	0.9981 to 1.004	0.4033

Risk factors for 60 days early mortality in patients treated with atezo/bev. Odd's ratio for early mortality risk was assessed by multivariate logistic regression. Abbreviations: CI, confidence interval; CPS, Child-Pugh score.

Table S3. Baseline characteristics of patients experiencing EM due to progressive disease or hepatic decompensation

	Progressive disease n=15	Hepatic decompensation n=14
**Variable**		
**Mean age ± SD (years)**	65.4±13.7	60.4±9.7
**Female (%)**	3 (20.0)	3 (21.4)
**Etiology***		
**HBV**	2 (13.3)	2 (14.3)
**HCV**	5 (33.3)	3 (21.4)
**Alcohol-related**	1 (6.7)	5 (35.1)
**MASLD/MASH**	2 (13.3)	1 (7.1)
**Other**	3 (20.0)	2 (14.3)
**Unknown**	2 (13.3)	2 (14.3)
**Child-Pugh category (%)**		
**A**	8 (53.3)	5 (35.7)
**B**	7 (46.7)	9 (64.3)
**Ascites (%)**		
**None**	10 (66.7)	6 (42.9)
**Present**	5 (33.3)	8 (57.1)
**Hepatic encephalopathy**		
**None**	15 (100)	12 (85.7)
**Present**	0 (0)	2 (14.3)
**ECOG PS ≥ 2 (%)**	2 (13.3)	2 (14.3)
**BCLC stage (%)**		
**B**	3 (20.0)	2 (14.3)
**C**	12 (80.0)	12 (85.7)
**Extrahepatic spread (%)**	10 (66.7)	5 (35.1)
**Macrovascular invasion (%)**	5 (33.3)	8 (57.1)
**ALBI grade**		
**1**	1 (6.7)	2 (14.3)
**2**	7 (46.7)	4 (28.6)
**3**	7 (46.7)	8 (57.1)
**Albumin (mean, mg/dl)**	3.2	3.1
**Bilirubin (mean, mg/dl)**	1.2	2.6
**AFP (mean, ng/ml)**	156271	36001

* = Several etiologies in one patient possible. ALBI, Albumin-Bilirubin; BCLC, Barcelona Clinic Liver Cancer Staging System; ECOG, Eastern Cooperative Oncology Group performance status; SD, standard deviation.

Table S4. Baseline characteristics translational cohort

Variable	EM cohort<br>n=8	Non-EM cohort<br>n=32	P-value
Mean age ± SD (years)	66.8±12.5	66.2±8.0	0.95
Female (%)	2 (25.0)	3 (9.4)	0.26
Etiology (%)			
Viral	1 (12.5)	9 (28.1)	0.65
Non-Viral	7 (87.5)	23 (71.9)	
Cirrhosis (%)	5 (62.5)	23 (71.9)	0.53
Child-Pugh (%)*			
A	3 (37.5)	28 (87.5)	**0.008**
B	5 (62.5)	4 (12.5)	**0.008**
C	0 (0.0)	0 (0.0)	>0.99
Ascites (%)			
yes	3 (37.5)	8 (25.0)	0.66
no	5 (62.5)	24 (75.0)	
Hepatic encephalopathy (%)			
yes	2 (25.0)	1 (3.1)	0.10
no	6 (75.0)	31 (96.9)	
ECOG PS ≥ 2 (%)	0 (0.0)	1 (3.1)	>0.99

BCLC stage (%)			
A	0 (0)	0 (0.0)	>0.99
B	2 (25.0)	7 (21.9)	>0.99
C	6 (75.0)	25 (78.1)	>0.99
D	0 (0.0)	0 (0.0)	>0.99
Extrahepatic spread (%)	3 (37.5)	18 (56.3)	0.44
Macrovascular invasion (%)	3 (37.5)	13 (40.6)	>0.99
Gastroesophageal varices (%)	4 (50.0)	9 (28.1)	0.40
Esophageal varices grade (%)			
none	4 (50.0)	23 (71.9)	0.40
I	4 (50.0)	8 (25.0)	0.21
II	0 (0.0)	0 (0.0)	>0.99
III	0 (0.0)	1 (3.1)	>0.99
Prior variceal bleeding (%)	1 (12.5)	0 (0.0)	0.20
NSBB (%)	2 (25.0)	3 (9.4)	0.26
Antiplatelet drugs (%)	1 (12.5)	8 (25.0)	0.66
Anticoagulation (%)	4 (50.0)	7 (21.9)	0.18

Baseline characteristics for translational cohort: early mortality versus non-early mortality. Continuous variables were reported as mean plus standard deviation and compared via independent samples t-test or Mann-Whitney U test, depending on the presence of a normal distribution. Categorical variables were reported as numbers and percentages. Comparisons of categorical variables were conducted by Fisher's exact test. P <0.05 was considered statistically significant. BCLC, Barcelona Clinic Liver Cancer Staging System; ECOG, Eastern Cooperative Oncology Group performance status; EM, early mortality; HCC, hepatocellular carcinoma; NSBB, non-selective beta blockers, SD, standard deviation.

Table S5. Best response in the early mortality cohort and non-early mortality cohort

**Variable**	**Early mortality cohort**<br>**n=50**	**Non-early mortality cohort**<br>**n=267**
**Best overall response n (%)**	1 (2)	91 (34.1)
**Complete response n (%)**	0 (0)	9 (3.4)
**Partial response n (%)**	1 (2)	82 (30.7)
**Stable disease n (%)**	10 (20)	86 (32.2)
**Disease control rate n (%)**	11 (22)	177 (66.3)
**Progressive disease n (%)**	7 (14)	74 (27.7)
**Missing n (%)**	32 (64)	16 (6)

Table S5. Baseline characteristics of patients experiencing EM with Child Pugh A or Child Pugh B

	Child-Pugh A n=25	Child-Pugh B n=25
**Variable**		
**Mean age ± SD (years)**	66.96±12.57	66.84±10.61
**Female (%)**	4 (16.0)	4 (16.0)
**Etiology***		
HBV	3 (12.0)	3 (12.0)
HCV	7 (28.0)	3 (12.0)
Alcohol-related	5 (20.0)	8 (32.0)
MASLD/MASH	5 (20.0)	0 (0)
Other	2 (8.0)	1 (4.0)
Unknown	3 (12.0)	11 (44.0)
**Ascites (%)**		
None	22 (88.0)	11 (44.0)
Present	2 (8.0)	14 (56.0)
Missing	1 (4.0)	0 (0)
**Hepatic encephalopathy**		
None	24 (96.0)	22 (88.0)
Present	0 (0)	3 (12.0)
Missing	1 (4.0)	0 (0)
**ECOG PS ≥ 2 (%)#**	1 (4.0)	7 (28.0)
**BCLC stage (%)**		
B	3 (12.0)	4 (16.0)
C	22 (88.0)	21 (84.0)
**Extrahepatic spread (%)#**	9 (36.0)	13 (52.0)
**Macrovascular invasion (%)#**	13 (52.0)	14 (56.0)
**ALBI grade**		
1	2 (8.0)	1 (4.0)
2	18 (72.0)	5 (20.0)
3	3 (12.0)	19 (76.0)
Missing	2 (8.0)	0 (0)
**Albumin (mean, mg/dl)**	3.5	2.9
**Bilirubin (mean, mg/dl)**	1.1	2.5
**AFP (mean, ng/ml)**	88948	24535

Continuous variables were reported as mean plus standard deviation and compared via independent samples t-test or Mann-Whitney U test, depending on the presence of a normal distribution. Categorical variables were reported as numbers and percentages. Comparisons of categorical variables were conducted by Fisher's exact test. P <0.05 was considered statistically significant. * = Several etiologies in one patient possible. # = In one patient, no data was available. BCLC, Barcelona Clinic Liver Cancer Staging System; ECOG, Eastern Cooperative Oncology Group performance status; EM, early mortality; HCC, hepatocellular carcinoma; NSBB, non selective beta blockers, SD, standard deviation.

Table S6. Univariate analysis of risk factors for 90 days early mortality in atezo/bev-treated patients with Child-Pugh A

Variable	Univariate Odd's ratio	95% (CI)	p-value
Age	0.9901	0.9548 to 1.029	0.6007
Sex (male)	1.537	0.5522 to 5.457	0.4508
ECOG PS ≥ 2	0.5472	0.02963 to 2.893	0.5677
Nonviral etiology	1.678	0.6937 to 3.914	0.2359
CPS A6 (A5 as reference)	4.235	1.773 to 10.90	**0.0016**
Ascites (no ascites as reference)	0.5515	0.08564 to 2.008	0.4363
BCLC C (B as reference)	2.204	0.7243 to 9.585	0.2144
Extrahepatic spread	0.7677	0.3101 to 1.804	0.5515
Macrovascular invasion	1.990	0.8499 to 4.738	0.1126
Presence of GE varices	1.643	0.6869 to 3.969	0.2616
History of variceal bleeding	2.217	0.3220 to 9.520	0.3318
Spleen size	1.143	0.9873 to 1.323	0.0698
NSBB	1.565	0.6072 to 3.749	0.3285
Anticoagulation	1.330	0.5378 to 3.115	0.5193
Albumin g/dl	0.1741	0.0626 to 0.442	**0.0004**
Bilirubin md/gl	2.205	1.017 to 4.635	**0.0382**
ALBI Grade 2 (Grade 1 as reference)	5.763	1.599 to 36.92	**0.0212**
ALBI Grade 3 (Grade 1 as reference)	15.64	2.251 to 135.5	**0.0057**
INR	4.347	0.2238 to 67.70	0.3081
Creatinine mg/dl	0.9497	0.2561 to 2.638	0.9301
Presence of proteinuria	1.064	0.3208 to 3.081	0.9132
CRP mg/dl	1.072	0.9768 to 1.169	0.1064
AFP ng/ml	1.000	1.000 to 1.000	0.1117
Leucocytes 10^9/L	0.9664	0.8096 to 1.135	0.6909
Neutrophils 10^9/L	0.9636	0.7886 to 1.033	0.5743
Platelets 10^9/L	1.002	0.9981 to 1.005	0.3251
Hemoglobin g/dl	0.7473	0.5993 to 0.9252	**0.0081**
MELD score	1.074	0.9672 to 1.193	0.1800
Neutrophils to platelets ratio	0.9938	0.9567 to 1.004	0.6211

Odd's ratio for early mortality risk was assessed by univariate logistic regression. Abbreviations: ALBI, Albumin-Bilirubin; BCLC, Barcelona Clinic Liver Cancer; CI, confidence interval; CPS, Child-Pugh score; ECOG, Eastern Cooperative Oncology Group Performance Status; GE varices, gastoesophageal varices; MELD, Model of Endstage Liver Disease; NSBB, non-selective beta-blockers.

Table S7. Multivariate analysis of risk factors for 90 days early mortality in atezo/bev-treated patients with Child-Pugh A

**Variable**	**Univariate Odd's ratio**	**95% (CI)**	**p-value**
ALBI Grade 2 (Grade 1 as reference)	4.382	1.167 to 28.58	0.0566
ALBI Grade 3 (Grade 1 as reference)	12.54	1.746 to 111.0	**0.0123**
Hemoglobin g/dl	0.7957	0.6308 to 0.9973	**0.0488**

Odd's ratio for early mortality risk was assessed by multivariate logistic regression. Abbreviations: ALBI, Albumin-Bilirubin; CI, confidence interval.

Table S8. Univariate analysis of risk factors for 90 days early mortality in atezo/bev-treated patients with Child-Pugh B

Variable	Univariate Odd's ratio	95% (CI)	p-value
Age	1.006	0.9621 to 1.055	0.7859
Sex (male)	0.5397	0.1368 to 1.783	0.3356
ECOG PS ≥ 2	4.324	1.156 to 18.36	**0.0337**
Nonviral etiology	0.8088	0.2559 to 2.718	0.7213
CPS B8 (B7 as reference)	1.175	0.3415 to 3.826	0.7912
CPS B9 (B7 as reference)	4.073	1.120 to 16.23	**0.0366**
Ascites. moderate (no ascites as reference)	0.6719	0.2290 to 1.944	0.4628
Ascites. severe (no ascites as reference)	1.030	0.2204 to 4.476	0.9684
Hepatic encephalopathy	1.432	0.2628 to 7.057	0.6568
BCLC C (B as reference)	1.853	0.5609 to 7.309	0.3356
Extrahepatic spread	1.685	0.6312 to 4.564	0.2981
Macrovascular invasion	1.069	0.4014 to 2.886	0.8937
Presence of GE varices	0.6111	0.2189 to 1.682	0.3404
History of variceal bleeding	3.000	0.4652 to 24.06	0.2472
Spleen size	0.9005	0.7380 to 1.084	0.2792
NSBB	0.4242	0.1416 to 1.175	0.1086
Anticoagulation	1.692	0.6010 to 4.749	0.3148
Albumin g/dl	0.1787	0.0497 to 0.528	**0.0040**
Bilirubin md/gl	1.406	1.011 to 2.138	0.0714
ALBI Grade 2 (Grade 1 as reference)	0.1667	0.00589 to 4.664	0.2305
ALBI Grade 3 (Grade 1 as reference)	1.267	0.04746 to 33.82	0.8710
INR	0.2607	0.01774 to 1.462	0.2203
Creatinine mg/dl	6.739	1.839 to 36.66	**0.0120**
Presence of proteinuria	1.037	0.1984 to 4.415	0.9623
CRP mg/dl	1.209	1.066 to 1.424	**0.0092**
AFP ng/ml	1.000	1.000 to 1.000	0.0522
Leucocytes 10^9/L	1.275	1.090 to 1.545	**0.0061**
Neutrophils 10^9/L	1.323	1.080 to 1.674	**0.0116**
Platelets 10^9/L	1.005	1.002 to 1.010	**0.0054**
Hemoglobin g/dl	0.9939	0.7999 to 1.234	0.9551
MELD score	1.120	1.010 to 1.257	**0.0403**
Neutrophils to platelets ratio	0.9933	0.9695 to 1.016	0.5334

Odd's ratio for early mortality risk was assessed by univariate logistic regression. Abbreviations: ALBI, Albumin-Bilirubin; BCLC, Barcelona Clinic Liver Cancer; CI, confidence interval; CPS, Child-Pugh score; ECOG, Eastern Cooperative Oncology

Group Performance Status; GE varices, gastoesophageal varices; MELD, Model of Endstage Liver Disease; NSBB, non-selective beta-blockers.

Table S9. Multivariate analysis of risk factors for 90 days early mortality in atezo/bev-treated patients with Child-Pugh B

Variable	Univariate Odd's ratio	95% (CI)	p-value
CPS B9	5.556	1.342 to 25.15	0.0196
ECOG PS ≥ 2	9.382	1.966 to 53.73	0.0070
Creatinine mg/dl	9.301	1.670 to 85.24	0.0292

Odd’s ratio for early mortality risk was assessed by multivariate logistic regression. Abbreviations: CI, confidence interval; CPS, Child-Pugh score; ECOG, Eastern Cooperative Oncology Group Performance Status.
