# Supplementary material for: Early mortality in atezolizumab/bevacizumab for HCC is associated with impaired liver function and alterations of systemic immunity
Source: JHEP Rep. 2025 Jul 5;7(11):101513. doi: 10.1016/j.jhepr.2025.101513 (PMC12861967; doi:10.1016/j.jhepr.2025.101513)
Supplement: Multimedia component 2 [file mmc2.docx]

**Journal of Hepatology**

**CTAT methods**

Tables for a “Complete, Transparent, Accurate and Timely account” (CTAT) are now mandatory for all revised submissions. The aim is to enhance the reproducibility of methods.

- Only include the parts relevant to your study
- Refer to the CTAT in the main text as ‘Supplementary CTAT Table’
- Do not add subheadings
- Add as many rows as needed to include all information
- Only include one item per row

**If the CTAT form is not relevant to your study, please outline the reasons why:**

|  |
| --- |

- 1. **Antibodies**

| **Name** | **Citation** | **Supplier** | **Cat no.** | **Clone no.** |
| --- | --- | --- | --- | --- |
| CD80 | www.biolegend.com | Biolegend | 305222 | 2D10 |
| CD8 | www.biolegend.com | Biolegend | 344731 | SK1 |
| PD-L1 | www.biolegend.com | Biolegend | 329723 | 29E2A3 |
| HLA-DR | www.biolegend.com | Biolegend | 307649 | L243 |
| CD19 | www.biolegend.com | Biolegend | 302245 | HIB19 |
| CD11c | www.biolegend.com | Biolegend | 337213 | Bu15 |
| CD4 | www.biolegend.com | Biolegend | 344607 | SK3 |
| CD86 | www.biolegend.com | Biolegend | 374205 | BU63 |
| CD14 | www.biolegend.com | Biolegend | 325633 | HCD14 |
| CD45RO | www.biolegend.com | Biolegend | 304229 | UCHL1 |
| CCR7 | www.biolegend.com | Biolegend | 353213 | G043H7 |
| CD3 | www.biolegend.com | Biolegend | 317339 | OKT3 |
| TIGIT | www.biolegend.com | Biolegend | 372709 | A15153G |
| ICOS | www.biolegend.com | Biolegend | 313537 | C398.4A |
| TIM-3 | www.biolegend.com | Biolegend | 345027 | F38-2E2 |
| CTLA4 | www.biolegend.com | Biolegend | 369631 | BNI3 |
| CD44 | www.biolegend.com | Biolegend | 369631 | C44Mab-5 |
| FOXP3 | www.biolegend.com | Biolegend | 320207 | 259D |
| LAG-3 | www.biolegend.com | Biolegend | 369331 | 11C3C65 |
| Granzyme B | www.biolegend.com | Biolegend | 372213 | QA16A02 |
| CD25 | www.biolegend.com | Biolegend | 356409 | M-T271 |
| mouse IgG1 | www.biolegend.com | Biolegend | 400157 | MOPC-21 |
| mouse IgG1 | www.biolegend.com | Biolegend | 400171 | MOPC-21 |
| mouse IgG2b | www.biolegend.com | Biolegend | 400349 | MOPC-11 |
| mouse IgG1 | www.biolegend.com | Biolegend | 400163 | MOPC-21 |
| mouse IgG1 | www.biolegend.com | Biolegend | 400167 | MOPC-21 |
| mouse IgG1 | www.biolegend.com | Biolegend | 981802 | MOPC-21 |
| mouse IgG1 | www.biolegend.com | Biolegend | 400149 | MOPC-21 |
| mouse IgG1 | www.biolegend.com | Biolegend | 319801 | MOPC-21 |
| mouse IgG1 | www.biolegend.com | Biolegend | 400177 | MOPC-21 |
| mouse IgG2a | www.biolegend.com | Biolegend | 400231 | MOPC-173 |
| mouse IgG2a | www.biolegend.com | Biolegend | 400219 | MOPC-173 |
| mouse IgG1 | www.biolegend.com | Biolegend | 400119 | MOPC-21 |
| mouse IgG2a | www.biolegend.com | Biolegend | 400247 | MOPC-173 |
| mouse IgG2a | www.biolegend.com | Biolegend | 400259 | MOPC-173 |
| Armenian Hamster IgG | www.biolegend.com | Biolegend | 400943 | HTK888 |
| mouse IgG2a | www.biolegend.com | Biolegend | 400271 | MOPC-173 |
| mouse IgG1 | www.biolegend.com | Biolegend | 400125 | MOPC-21 |

- 1. **Cell lines**

| **Name** | **Citation** | **Supplier** | **Cat no.** | **Passage no.** | **Authentication test method** |
| --- | --- | --- | --- | --- | --- |
|  |  |  |  |  |  |

- 1. **Organisms**

| **Name** | **Citation** | **Supplier** | **Strain** | **Sex** | **Age** | **Overall n number** |
| --- | --- | --- | --- | --- | --- | --- |
|  |  |  |  |  |  |  |

- 1. **Sequence based reagents**

| **Name** | **Sequence** | **Supplier** |
| --- | --- | --- |
|  |  |  |

- 1. **Biological samples**

| **Description** | **Source** | **Identifier** |
| --- | --- | --- |
|  |  |  |

- 1. **Deposited data**

| **Name of repository** | **Identifier** | **Link** |
| --- | --- | --- |
|  |  |  |

- 1. **Software**

| **Software name** | **Manufacturer** | **Version** |
| --- | --- | --- |
| GraphPad Prism | GraphPad Software, San Diego, CA, USA | 9 |
| FlowJo software | BD Biosciences | 10 |
| Affinity designer | Serif Ltd | 2 |

- 1. **Other (e.g. drugs, proteins, vectors etc.)**

|  |  |  |
| --- | --- | --- |
|  |  |  |

- 1. **Please provide the details of the corresponding methods author for the manuscript:**

| Ignazio Piseddu & Najib Ben Khaled  LMU University Hospital  Department of Medicine II  Marchioninistrasse 15  81377 Munich, Germany  [Ignazio.piseddu@med.uni-muenchen.de](mailto:Ignazio.piseddu@med.uni-muenchen.de)  Najib.benkhaled@med.uni-muenchen.de  phone: 089 4400 78160 |
| --- |

**2.0 Please confirm for randomised controlled trials all versions of the clinical protocol are included in the submission. These will be published online as supplementary information.**

|  |
| --- |
