# Supplementary material for: Early mortality in atezolizumab/bevacizumab for HCC is associated with impaired liver function and alterations of systemic immunity
Source: JHEP Rep. 2025 Jul 5;7(11):101513. doi: 10.1016/j.jhepr.2025.101513 (PMC12861967; doi:10.1016/j.jhepr.2025.101513)
Supplement: Multimedia component 4 [file mmc4.pdf]

JHEP Reports

Early mortality in atezolizumab/bevacizumab for HCC is associated with impaired liver function and alterations of systemic immunity

Authors

Ignazio Piseddu, Leonie S. Jochheim, Katrin Boettcher, …, Enrico N. De Toni, Florian P. Reiter, Najib Ben Khaled

Correspondence

Najib.benkhaled@med.uni-muenchen.de (N. Ben Khaled).

Graphical abstract

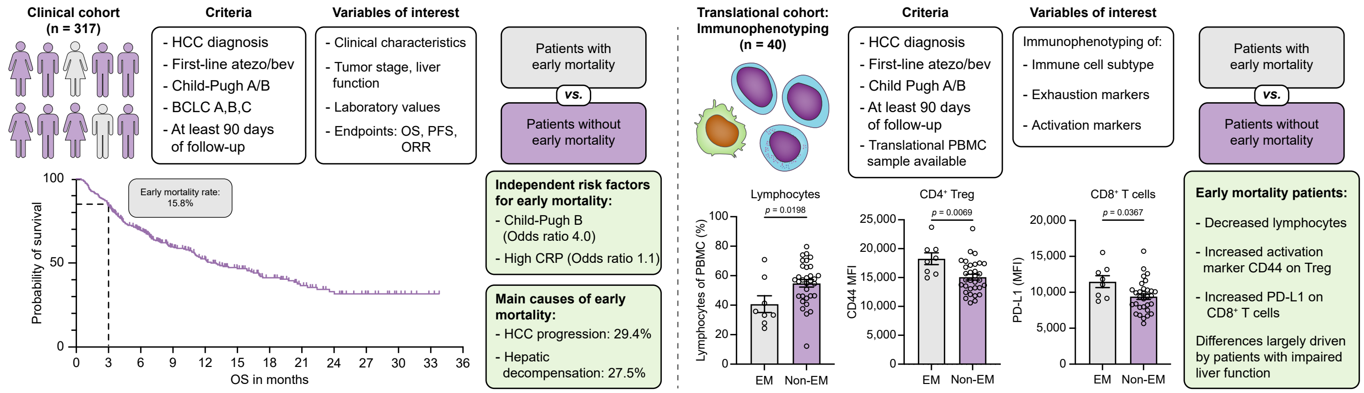

Highlights:

- Early mortality occurs in 15.8% of patients with HCC treated with atezo/bev.
- Impaired liver function is a key predictor of early mortality.
- High CRP, reduced lymphocyte frequencies and increased Treg activity are associated with increased mortality risk.
- Findings might help clinicians to identify patients at high risk of early death.

Impact and implications:

Although rare in clinical trial populations, early mortality (EM) is a significant concern following the initiation of immune checkpoint inhibitors in patients with hepatocellular carcinoma (HCC) in real-world cohorts, highlighting the need for adequate risk stratification and patient selection. Using data of a large, European real-world cohort as well as FACS-based immunophenotyping of baseline peripheral blood mononuclear cells, we comprehensively characterized the frequency, risk factors, and biomarkers associated with EM in atezo/bev-treated patients with HCC. We identified EM to represent a common event in patients with HCC after treatment initiation and demonstrated impaired liver function, elevated biomarkers of inflammation, and reduced lymphocyte frequencies and increased regulatory T cell activity to be associated with increased EM risk. These findings can help clinicians identify patients with HCC at high risk of EM, enabling adequate and informed decision-making regarding end-of-life care and palliative treatment.

https://doi.org/10.1016/j.jhepr.2025.101513

© 2025 The Authors. Published by Elsevier B.V. on behalf of European Association for the Study of the Liver (EASL). This is an open access article under the CC BY license (http://creativecommons.org/licenses/by/4.0/).

Early mortality in atezolizumab/bevacizumab for HCC is associated with impaired liver function and alterations of systemic immunity

Ignazio Piseddu[1,2,3], Leonie S. Jochheim[4], Katrin Boettcher[3,5], Bernhard Scheiner[6], Friedrich Sinner[7], Simon Johannes Gairing[8], Matthias Thaler[2], Stefan Enssle[1,3], Monika Karin[1,3], Valentina Zarka[3,9], Alexander Philipp[1,3], Andreas Thalmeier[10], Jan Gaertig[2], Lorenz Balcar[6], Julia Martina Schütte[4], Julia S. Schneider[1,3], Katarina Ondrejkova[1], Monika Rau[9], Alexander Weich[11], David Anz[1,2], Karin Berger[9], Christian Schulz[1], Christian M. Lange[1,4], Osman Öcal[12], Marianna Alunni-Fabbroni[12], Jens Ricke[12], Ursula Ehmer[5], Marino Venerito[7], Friedrich Foerster[8], Matthias Pinter[6], Andreas Geier[3,11], Julia Mayerle[1], Enrico N. De Toni[1,3], Florian P. Reiter[3,11], Najib Ben Khaled[1,3,13,*]

JHEP Reports **2025**. vol. ■ | 1–12

Background & Aims: Atezolizumab/bevacizumab (atezo/bev) has revolutionized the standard of care for patients with unresectable hepatocellular carcinoma (HCC). However, only a subgroup of patients responds to atezo/bev and derives durable clinical benefit. This study aims to analyze the frequency and risk factors of early mortality (EM) in patients with HCC treated with atezo/bev.

Methods: This study uses data from a large, European real-world cohort and flow cytometry-based immunophenotyping of patient's baseline PBMC. EM was defined as death from any cause within 90 days of treatment initiation. Logistic regression analysis was used to identify parameters associated with EM.

Results: A total of 317 patients with unresectable HCC treated with first-line atezo/bev were included. EM rate in the cohort was 15.8%, with a median survival of 12.6 months. The proportion of patients with preserved liver function and BCLC stage B was significantly lower in the EM cohort. The strongest predictor of EM was advanced liver disease in univariate analysis, as reflected by surrogates of impaired liver function such as Child–Pugh score (CPS) B (p <0.0001), albumin–bilirubin grade 2/3 (p = 0.026, p <0.0001) or high model for end-stage liver disease score (p <0.0001). CPS B remained a significant risk factor after adjusting for other variables. Biomarker analysis and immunophenotyping revealed high C-reactive protein, reduced lymphocyte frequencies, increased CD44 expression on regulatory T cells and elevated PD-L1 levels on $CD8^+$ T cells to be associated with EM.

Conclusions: EM rate was 15.8% in patients with HCC treated with atezo/bev. Significant risk factors for early death involved impaired liver function, elevated biomarkers of inflammation and alterations of systemic immunity.

© 2025 The Authors. Published by Elsevier B.V. on behalf of European Association for the Study of the Liver (EASL). This is an open access article under the CC BY license (http://creativecommons.org/licenses/by/4.0/).

Introduction

Hepatocellular carcinoma (HCC) ranks among the most lethal cancer entities worldwide, with approximately 800,000 deaths in 2020.[1,2] The approval of the immune checkpoint inhibitor (ICI) atezolizumab in combination with the vascular endothelial growth factor (VEGF) inhibitor bevacizumab (atezo/bev) has revolutionized the treatment landscape for unresectable HCC, significantly improving patient outcomes as compared with the previous first-line standard sorafenib as demonstrated in the pivotal IMbrave150 trial.[3,4] However, real-world cohorts tend to be sicker than study populations, especially in HCC. Most patients have chronic liver disease and individuals treated in clinical practice frequently have more severe liver dysfunction as compared with trial participants.[5]

One significant concern in this regard is early mortality (EM) following the initiation of immunotherapy.[6] EM is rare in clinical trial populations. In the IMbrave150 study, around 95% of patients were still alive 3 months after therapy initiation.[4] However, because of strict eligibility criteria, these patients represent a highly selected cohort. Real-world data from other cancer entities suggests a higher incidence of EM upon ICI administration. In this context, a Canadian study reported 60-day and 90-day mortality rates as high as 15% and 22% in various cancers, excluding HCC.[7] A similar dimension of early death was seen in two retrospective analyses of lung cancer patients, with rates reported to be 15%[8] and 13%.[9] These findings suggest that patients in real-world settings have a higher risk of EM compared with those in clinical trials. To date,

* Corresponding author. Address: Department of Medicine II, University Hospital, LMU Munich, Marchioninistrasse 15, Munich, Germany. Tel.: +49 89 4400 78160.
E-mail address: Najib.benkhaled@med.uni-muenchen.de (N. Ben Khaled).
https://doi.org/10.1016/j.jhepr.2025.101513

the frequency and risk factors of early death in patients with HCC receiving atezo/bev have not been thoroughly investigated. Understanding these factors could improve patient selection and monitoring, enhancing treatment safety and ensuring that atezo/bev is allocated to those most likely to benefit.

In this multicenter study, we aimed to assess the characteristics of patients with HCC experiencing EM after initiation of atezo/bev and identify clinical characteristics, biomarkers, and immunophenotypes associated with early death in a large European real-world cohort.

Materials and methods

Patient population

This study was initiated by the IMMUreal study group. The objective of the IMMUreal study group is to investigate the efficacy of immunotherapeutic agents for the treatment of liver tumors. Patient data were collected from six centers in Germany (University Hospital LMU Munich, University Hospital Würzburg, University Hospital Essen, Klinikum rechts der Isar TU Munich, University Hospital Magdeburg, and University Medical Center Mainz) and one center in Austria (Medical University of Vienna). All patients included in this study had a confirmed HCC diagnosis based on histopathological findings or characteristic diagnostic imaging according to the European Association for the Study of the Liver (EASL) criteria.[10] Patients were divided into an EM cohort and a Non-EM cohort. The main analysis was conducted using a cut-off of 90 days for EM *vs.* Non-EM. Patients with insufficient follow-up <90 days were excluded to prevent underestimation of EM rates. We chose the 90-day cut-off because it represents a well-established threshold for mortality risk in hepatology and was used, for example, in the development of the model for end-stage liver disease (MELD) score.[11] A confirmatory subgroup analysis divided patients into EM and Non-EM based on a 60-day cut-off, with patients with insufficient follow-up <60 days being excluded. The cut-off of 60-day mortality was chosen since it is an accepted parameter for evaluating the safety of systemic therapies for gastrointestinal cancers.[12] As patients with Child–Pugh score (CPS) C and Barcelona Clinic liver cancer (BCLC) D are excluded from current recommendations and skewed the analysis because of the high risk of early death, these patients were excluded. To analyze the impact of clinical trial eligibility criteria, patients were grouped into those meeting IMbrave150 inclusion criteria (IMbrave150-IN) *vs.* patients not meeting the IMbrave150 criteria (IMbrave150-OUT). Patients were classified as IMbrave150-IN, if they met the following criteria: CPS A, Eastern Cooperative Oncology Group (ECOG) 0–1, no moderate or severe ascites, no history of hepatic encephalopathy (HE), no current or recent of full-dose anticoagulants for therapeutic purpose, no current or recent use of high-dose aspirin or other antiplatelets such as clopidogrel, prior variceal bleeding event within 6 months before start of atezo/bev, laboratory markers (albumin, bilirubin, creatinine, platelets, neutrophils, hemoglobin, international normalized ratio [INR]) within IMbrave150 eligibility criteria.[4] The study was approved by local authorities and conducted in accordance with the Declaration of Helsinki. The Strengthening the Reporting of Observational Studies in Epidemiology (STROBE) checklist was used during manuscript preparation.[13] Some patient data from some centers have been used in other projects.[14–19]

Ethics approval and consent

The study protocol was approved by the institutional review board (Ethics committees of the Medical Faculty of the Ludwig-Maximilians-Universität Munich [18-604, 20-439, 25-0223], University Medical Center of Mainz [837.199.10], Julius-Maximilians-Universität Würzburg [156/21-me], University Hospital Essen [21-71009-BO], Medical University of Vienna [2033/2017 and 1759/2015], Otto-von-Guericke University Magdeburg [70/22], and Klinikum rechts der Isar of the Technical University Munich [2022-605-S-KH]). Patient informed consent was obtained to perform biomarker analyses (20-439).

Treatments and assessments

Patients received the following treatment regimen: atezo/bev, with atezolizumab being administered intravenously at a dose of 1,200 mg and bevacizumab at 15 mg per kg of body weight every 3 weeks. Monitoring of patients included clinical, laboratory, and imaging evaluations, aligning with the established standard of care per the German HCC guidelines.[20] The assessment of tumor response was systematically conducted every 8–12 weeks through computed tomography and/or magnetic resonance imaging. Hepatic decompensation as death cause was defined following the statement by D'Amico *et al*.[1] as new onset or worsening of ascites, variceal bleeding, and new onset or worsening of HE. Patients with pre-existing ascites were considered to have hepatic decompensation if ascites worsened during the observation period, necessitating paracentesis. Patients with pre-existing HE were considered to have hepatic decompensation if HE worsened as per Westhaven Criteria. Treatment-related adverse events were identified and treated following the guidance provided in the Summary of Product Characteristics and in the current guidelines, without application of predefined time cut-offs.[21,22]

Endpoints

The primary endpoint of this study was to investigate the occurrence and risk factors of EM, defined as death of any cause within 3 months of therapy initiation. Secondary efficacy endpoints included overall survival, progression-free survival, and response rates. Overall survival (OS) was defined as the time from commencement of treatment until death from any cause. Progression-free survival (PFS) referred to the period from treatment initiation to the occurrence of disease progression on radiological assessment or death from any cause. Patients without recorded OS or PFS events or patients who were lost to follow-up were censored at the date of their most recent contact. Radiological response was characterized as complete or partial response (CR/PR), stable or progressive disease (SD/PD) by the local investigator. Overall response rate (ORR) was the proportion of patients experiencing CR and PR, while disease control rate was defined as CR plus PR plus SD.

PBMC isolation and FACS analysis

Peripheral blood mononuclear cells (PBMCs) obtained before therapy initiation in one study center (EM: n = 8, Non-EM: n = 32) were isolated from patients' EDTA-preserved whole blood by conventional Ficoll-Paque density gradient (Cytiva, Uppsala, Sweden) according to the manufacturer's instruction and preserved in fetal calf serum (FCS)/10% DMSO at -150 °C until analyzed. After isolation, cells were resuspended in cryopreservation medium consisting of 90% FCS and 10% DMSO, cooled down to -80 °C in isopropanol-containing freezing containers for 1–3 days and then transferred to liquid nitrogen. For FACS analysis, PBMCs were thawed and rested in RPMI1640 medium containing 10% FCS, 1% penicillin/streptomycin and 1% L-glutamine for 1 h. Before antibody staining, cells were treated with Human TruStain FcX (BioLegend, San Diego, USA). Extracellular staining was performed with 1:200 dilution of antibodies and eBioscience™ Fixable Viability Dye eFluor™ 780 (Invitrogen, Waltham, USA). Upon permeabilization and fixation of cells with eBioscience™ Foxp3/Transcription Factor Staining Buffer Set (Invitrogen), cells were intracellularly stained with 1:100 diluted antibodies. Samples were measured on a BD LSRFortessa Cell Analyzer (BD Biosciences, Franklin Lakes, USA) and data were analyzed using FlowJo 10.8.1 (BD Biosciences). Staining and isotype antibodies (all purchased from Biolegend) with respective antigens, fluorophores and clones were used as shown in Box 1.

Box 1. Antibodies for flow cytometry.

Antigen	Fluorophore	Clone
CD80	BV421	2D10
CD8	BV510	SK1
PD-L1	BV605	29E2A3
HLA-DR	BV650	L243
CD19	BV711	HIB19
CD11c	FITC	Bu15
CD4	PerCP-Cy5.5	SK3
CD86	PE	BU63
CD14	PE/Dazzle 594	HCD14
CD45RO	PE-Cy7	UCHL1
CCR7	APC	G043H7
CD3	AF700	OKT3
TIGIT	BV421	A15153G
ICOS	BV605	C398.4A
TIM-3	BV650	F38-2E2
CTLA4	BV711	BNI3
CD44	FITC	C44Mab-5
FOXP3	PE	259D
LAG-3	PE/Dazzle 594	11C3C65
Granzyme B	PE-Cy7	QA16A02
CD25	APC	M-T271
Mouse IgG1	BV421	MOPC-21
Mouse IgG1	BV510	MOPC-21
Mouse IgG2b	BV605	MOPC-11
Mouse IgG1	BV650	MOPC-21
Mouse IgG1	BV711	MOPC-21
Mouse IgG1	FITC	MOPC-21
Mouse IgG1	PerCP-Cy5.5	MOPC-21
Mouse IgG1	PE	MOPC-21
Mouse IgG1	PE/Dazzle 94	MOPC-21
Mouse IgG2a	PE-Cy7	MOPC-173
Mouse IgG2a	APC	MOPC-173
Mouse IgG1	APC	MOPC-21
Mouse IgG2a	AF700	MOPC-173
Mouse IgG2a	BV421	MOPC-173
Armenian Hamster IgG	BV605	HTK888
Mouse IgG2a	BV711	MOPC-173
Mouse IgG1	PE-Cy7	MOPC-21

Statistical analysis

Statistical evaluation was conducted utilizing GraphPad Prism 9 (GraphPad Software, San Diego, CA, USA). Patients characteristics were summarized using descriptive statistical methods. The conformity of variables to a normal distribution was examined using the Shapiro–Wilk test alongside qq-plot inspection. Continuous data were expressed as the mean with standard deviation and analyzed via the *t* test or Mann–Whitney *U* test based on their distribution characteristics. Categorical data were shown as frequencies and proportions. The comparison of categorical variables employed Fishers exact test. The Kaplan–Meier method was applied to estimate the median OS (mOS), with the log-rank test used for comparison. Odds ratios (ORs) for events were determined using both univariate and multivariate logistic regression analyses. To address collinearity between some continuous variables (hemoglobin, platelets) and the intercept, we centered the variables by subtracting the mean from each value. For multiple logistic regression, we selected the five variables with the highest ORs from the significant univariate results, reflecting the strongest associations with mortality. FACS data were analyzed using the Mann–Whitney *U* test. Values of $p < 0.05$ were denoted as statistically significant.

Results

Baseline characteristics

A total of 317 patients with HCC treated with atezo/bev were included in the analysis set (Fig. 1). Demographics and HCC

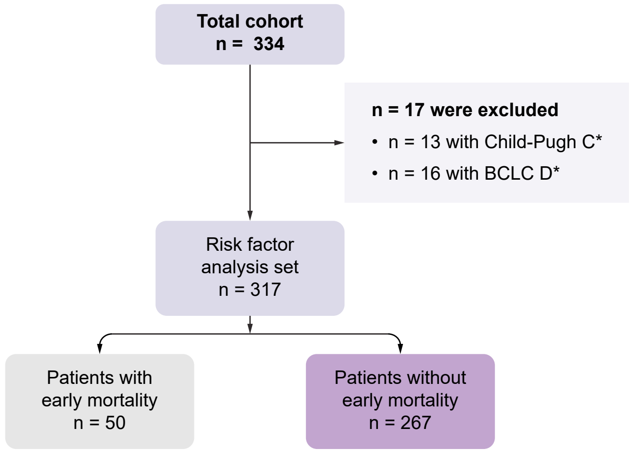

Fig. 1. Study flow chart. *Cases can overlap. BCLC, Barcelona Clinic Liver Cancer Staging System.

Table 1. Baseline characteristics.

	EM cohort n = 50	Non-EM cohort n = 267	*p* value
Variable			
Mean age ± standard deviation (years)	66.9 ± 11.5	67.82 ± 10.6	0.52
Female (%)	8 (16.0)	62 (23.2)	0.35
Etiology*			
HBV	6 (12.0)	23 (8.6)	0.43
HCV	10 (20.0)	51 (19.1)	0.85
Alcohol-related	13 (26.0)	64 (24.0)	0.72
MASLD/MASH	5 (10.0)	56 (21.0)	0.08
Other	6 (12.0)	42 (15.7)	0.66
Unknown	13 (26.0)	48 (18.0)	0.24
Cirrhosis (%)	35 (70.0)	189 (70.8)	>0.99
Child–Pugh category (%)			
A	25 (50.0)	212 (79.4)	**<0.001**
B	25 (50.0)	46 (17.2)	**<0.001**
Missing	0 (0)	9 (3.4)	0.36
Child–Pugh score (%)			
A5	8 (16.0)	144 (53.9)	**<0.001**
A6	16 (32.0)	68 (25.5)	0.38
B7	11 (22.0)	28 (10.5)	**0.03**
B8	6 (12.0)	13 (4.9)	0.09
B9	8 (16.0)	5 (1.9)	**<0.001**
Missing	1 (2.0)	9 (3.4)	>0.99
Ascites (%)			
None	33 (66.0)	208 (77.9)	0.074
Moderate	12 (24.0)	53 (19.9)	0.56
Severe	4 (8.0)	6 (2.25)	0.056
Missing	1 (2.0)	0 (0)	0.16
Hepatic encephalopathy			
None	46 (92.0)	263 (98.5)	**0.024**
Mild	3 (6.0)	4 (1.5)	0.081
Severe	0 (0)	0 (0)	>0.99
Missing	1 (2.0)	0 (0)	0.16
ALBI grade			
1	3 (6.0)	78 (29.2)	**<0.001**
2	23 (46.0)	147 (55.1)	0.28
3	22 (44.0)	22 (8.2)	**<0.001**
Missing	2 (4.0)	20 (7.5)	0.55
ECOG PS ≥2 (%)†	8 (16.3)	19 (7.1)	**0.048**
BCLC stage (%)			
B	7 (14.0)	63 (23.6)	0.19
C	43 (86.0)	204 (76.4)	
Extrahepatic spread (%)#	22 (44.9)	116 (43.4)	0.88
Macrovascular invasion (%)#	27 (55.1)	106 (39.7)	0.06
Baseline gastroscopy (%)	46 (92.0)	244 (91.4)	>0.99
Gastroesophageal varices (%)	23 (46.0)	104 (39.0)	0.35
Esophageal varices grade (%)			
None	23 (46.0)	140 (52.4)	0.44
I	16 (32.0)	61 (22.8)	0.21
II	6 (12.0)	36 (13.5)	>0.99
III	1 (2.0)	6 (2.2)	>0.99
Missing	4 (8.0)	24 (9.0)	>0.99
Prior variceal bleeding (%)	5 (10.0)	10 (3.7)	0.07
NSBB (%)	15 (30.0)	72 (27.0)	0.73
Anticoagulation (%)	19 (38.0)	78 (29.2)	0.24
Antiplatelet drugs (%)	10 (20.0)	72 (27.0)	0.38
IMbrave150-IN (%)	9 (18.0)	103 (38.6)	**0.006**

Continuous variables were reported as mean plus standard deviation and compared via independent samples *t* test or Mann–Whitney *U* test, depending on the presence of a normal distribution. Categorical variables were reported as numbers and percentages. Comparisons of categorical variables were conducted by Fisher's exact test. A value of *p* <0.05 was considered statistically significant.
Bold font indicate significant *p* values.
*Several etiologies in one patient possible.
†In one patient, no data was available. BCLC, Barcelona Clinic Liver Cancer Staging System; ECOG, Eastern Cooperative Oncology Group performance status; EM, early mortality; HCC, hepatocellular carcinoma; IMbrave150-IN, patient meeting inclusion criteria for the IMbrave150 trial; MASH, metabolic dysfunction-associated steatohepatitis; MASLD, metabolic dysfunction-associated steatotic liver disease; NSBB, non-selective beta blockers.

etiology were similar between the EM and Non-EM groups (Table 1). Underlying liver cirrhosis was present in the majority of cases (EM: n = 35/50 [70.0%] *vs.* Non-EM: n = 189/267 [70.8%] [*p* >0.99]), but liver function was notably poorer in patients with EM. This was evidenced by a significantly higher frequency of patients with CPS B (EM: n = 25/50 [50.0%] *vs.*

Non-EM: n = 46/267 [17.2%] [p <0.001]). CPS A was more common in patients who were Non-EM (EM: n = 25/50 [50.0%] *vs.* Non-EM: n = 212/267 [79.1%] [p <0.001]). Accordingly, ALBI grade 1 (EM: n = 3/50 (6.0%) *vs.* Non-EM: n = 78/267 [29.2%] [p <0.001]) was more frequent in patients who were Non-EM, whereas ALBI grade 3 was more common in the EM cohort (EM: n = 22/50 [44.0%] *vs.* Non-EM: n = 22/267 [8.2%] [p <0.001]). ECOG performance status was worse in the EM group, with an ECOG ≥2 attributed to 16.3% of patients with EM *vs.* 7.1% of Non-EM (p = 0.048). In this cohort, 112 patients (35.3%) met IMbrave150 eligibility criteria. IMbrave150-IN status was significantly more common among patients who were Non-EM (n = 103/267 [38.6%]) as compared with patients with EM (n = 9/50 [18.0%], p = 0.006).

Risk factors for EM and causes of death

EM, defined as death within the first 90 days of treatment, was observed in 15.8% of patients (n = 50/317). At a median follow-up of 15.5 months, the mOS and median PFS (mPFS) for the entire cohort were 12.6 months and 5.8 months, respectively (Fig. 2A and B). ORR was 29%, consisting of complete responses in nine patients (2.7%) and partial responses in 83 (26.2%) (Fig. 2C). SD was observed in 97 patients (30.6%) and PD in 82 (25.9%). Disease control could be achieved in 59.6%. mOS in patients who were CPS A and CPS B was 17.1 months and 4.2 months and mPFS 6.8 months and 3.2 months, respectively, which is similar to patient outcomes reported in the Imbrave150 trial[4] and other real-world cohorts.[16,23] Patients with EM had an mOS of 1.7 months (Fig. S1) with an mPFS of 1.2 months (Fig. S2) and an ORR of 2% (Table S5).

The primary objective was to assess the risk factors associated with EM in patients treated with atezo/bev (Table 2). Univariate regression analysis revealed that a poor performance status with an ECOG ≥2 significantly increased the risk of death within the first 90 days (OR 2.55, 95% CI 0.99–6.03, p = 0.039). Advanced cirrhosis, as evidenced by CPS B (OR 4.61, 95% CI 2.43–8.78, p <0.0001), was strongly associated with an increased risk of EM. High risk for early death in patients with reduced liver function was mirrored by significantly increased risk in patients with severe ascites (OR 4.2, 95% CI 1.03–15.51, p = 0.033). Whereas extrahepatic spread (OR 1.06, 95% CI 0.57–1.96, p = 0.851) did not affect the risk of early death, macrovascular invasion was significantly associated with EM (OR 1.86, 95% CI 1.01–3.47, p = 0.047). We further investigated laboratory values associated with EM in patients with HCC who were treated with atezo/bev. Low albumin (OR 0.14, 95% CI 0.07–0.25, p <0.0001) and high bilirubin levels (OR 2.06, 95% CI 1.5–2.94, p <0.0001) were significantly associated with increased risk of death within 90 days upon atezo/bev initiation. Additionally, C-reactive protein (CRP) levels were found to be significantly higher in patients experiencing EM (OR 1.13, 95% CI 1.06–1.21, p = 0.0006). Regarding blood count markers, increased numbers of white blood cells (OR 1.13, 95% CI 1.03–1.25, p = 0.012), lower hemoglobin (OR 0.81, 95% CI 0.7–0.94, p = 0.006) and higher platelets (OR 1.0, 95% CI 1.0–1.0, p = 0.006) were associated with EM. Alpha-fetoprotein (AFP) levels as surrogates of tumor aggressiveness were not linked to increased EM risk (OR 1.0, 95% CI 1.0–1.0, p = 0.133). Scores reflecting liver function were significantly associated with an increased risk of early death. Albumin–bilirubin (ALBI) score grade 2 (OR 4.07, 95% CI 1.36–17.52, p = 0.026) and ALBI grade 3 (OR 26.0, 95% CI 8.1–117.3, p <0.0001) strongly increased EM risk in our patient cohort. CPS A6 (OR 4.24, 95% CI 1.77–10.9, p = 0.0016), CPS B7 (OR 7.07, 95% CI 2.63–19.82, p = 0.0001), CPS B8 (OR 8.31, 95% CI 2.42–27.81, p = 0.0006) and CPS B9 (OR 28.8, 95% CI 7.94–116.9, p <0.001), were substantially associated with early death compared to CPS A5. Higher MELD scores observed in patients with EM were also linked to increased EM risk (OR 1.14, 95% CI 1.07–1.22, p <0.0001). Patients who would have been excluded from the IMbrave150 trial

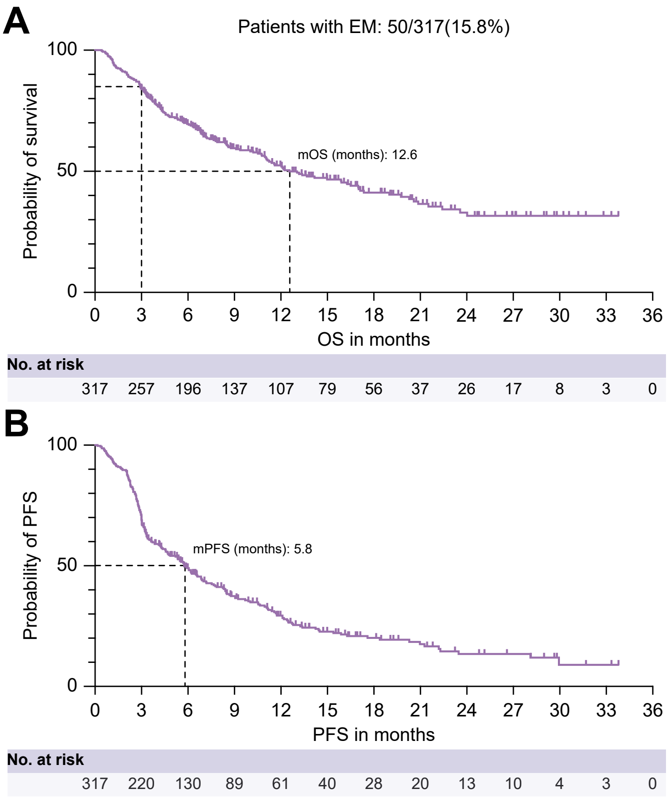

C

Best response (local investigator evaluation)

	Atezo/bev n = 317
**ORR-no. (%)**	92 (29.0)
**DCR-no. (%)**	188 (59.6)
**Best overall response**	
CR-no (%)	9 (2.8)
PR-no (%)	83 (26.2)
SD-no (%)	96 (30.3)
PD-no (%)	81 (25.6)
Missing-no (%)	48 (15.1)

Fig. 2. Efficacy analysis of atezolizumab/bevacizumab in all patients. (A) Median overall survival (mOS) with proportion of patients experiencing early mortality, (B) median progression-free survival, and (C) best response. The Kaplan–Meier method was applied to estimate the median survival. CR, complete response; DCR, disease control rate; EM, early mortality; mOS, median overall survival; ORR, objective response rate; mPFS, median progression-free survival; PD, progressive disease; PR, partial response.

Table 2. Univariable analysis of risk factors for early mortality in patients treated with atezo/bev.

Variable	Univariable odds ratio	95% CI	*p* value
Age	0.99	0.97–1.02	0.578
Sex (male)	1.59	0.74–3.81	0.262
ECOG PS ≥2	2.55	0.99–6.03	**0.039**
Liver cirrhosis	0.96	0.51–1.91	0.911
Non-viral etiology	0.72	0.38–1.43	0.341
CPS B (CPS A as reference)	4.61	2.43–8.78	**<0.0001**
CPS A6 (A5 as reference)	4.24	1.77–10.9	**0.0016**
CPS B7 (A5 as reference)	7.07	2.63–19.82	**0.0001**
CPS B8 (A5 as reference)	8.31	2.42–27.81	**0.0006**
CPS B9 (A5 as reference)	28.8	7.94–116.9	**<0.0001**
Ascites, moderate (no ascites as reference)	1.43	0.67–2.89	0.337
Ascites, severe (no ascites as reference)	4.2	1.03–15.51	**0.033**
Hepatic encephalopathy	4.29	0.82–20.06	0.062
BCLC C (A-B as reference)	1.9	0.86–4.8	0.139
Extrahepatic spread	1.06	0.57–1.96	0.851
Macrovascular invasion	1.86	1.01–3.47	**0.047**
Presence of GE varices	1.35	0.71–2.54	0.356
Esophageal varices grade I	1.6	0.78–3.22	0.194
Esophageal varices grade II	1.01	0.35–2.54	0.977
Esophageal varices grade III	1.01	0.05–6.32	0.99
History of variceal bleeding	2.86	0.86–8.44	0.066
Spleen size	1.09	0.98–1.22	0.108
NSBB	1.16	0.58–2.22	0.659
Anticoagulation	1.48	0.78–2.77	0.218
Antiplatelets	0.68	0.31–1.38	0.304
Albumin (g/dl)	0.14	0.07–0.25	**<0.0001**
Bilirubin (mg/dl)	2.06	1.5–2.94	**<0.0001**
ALBI Grade 2 (Grade 1 as reference)	4.07	1.36–17.52	**0.026**
ALBI Grade 3 (Grade 1 as reference)	26.0	8.1–117.3	**<0.0001**
INR	1.95	0.55–6.12	0.258
Creatinine (mg/dl)	1.26	0.82–1.98	0.243
Presence of proteinuria	0.97	0.38–2.25	0.941
CRP (mg/dl)	1.13	1.06–1.21	**0.0006**
AFP (ng/ml)	1.0	1.0–1.0	0.133
Leucocytes (10^9/L)	1.13	1.03–1.25	0.012
Neutrophils (10^9/L)	1.0	0.94–1.04	0.927
Platelets (10^9/L)	1.0	1.0–1.0	**0.006**
Hemoglobin (g/L)	0.81	0.7–0.94	**0.006**
MELD score	1.14	1.07–1.22	**<0.0001**
Neutrophils to platelets ratio	1.0	0.99–1.0	0.869
IMbrave150-OUT (IMbrave150-IN as reference)	2.86	1.39–6.50	**0.0069**

Risk factors for early mortality in patients treated with atezo/bev. Odd's ratio for early mortality risk was assessed by univariable logistic regression. *P* <0.05 was considered statistically significant. ALBI, Albumin–bilirubin; BCLC, Barcelona Clinic Liver Cancer; CI, confidence interval; CPS, Child–Pugh score; ECOG, Eastern Cooperative Oncology Group Performance Status; GE varices, gastroesophageal varices; IMbrave150-OUT, patient not meeting inclusion criteria for the IMbrave150 trial; MELD, model for end-stage liver disease; NSBB, non-selective beta blockers.
Bold font indicate significant *p* values.

(IMbrave150-OUT), had a significantly higher risk of early death (OR 2.86, 95% CI 1.39–6.50, *p* = 0.0069).

In multivariate regression analysis, reduced liver function parameters (CPS B: OR 3.96, 95% CI 1.95–8.01, *p* = 0.0001) and higher serum CRP (OR 1.09, 95% CI 1.01–1.18, *p* = 0.026) persisted as independent risk factors for EM (Table 3). As a 60-day cut-off for EM was also proposed for other systemic therapies,[12] we additionally evaluated factors associated with increased risk of death within 60 days of atezo/bev initiation (Tables S1 and S2). Poor liver function and higher serum CRP values continued to represent an independent risk factor for early death in both univariate and multivariate regression models. We performed additional analyses for further risk stratification in patients with impaired and preserved liver function. Patients with poor performance status of ECOG ≥2 were enriched among patients with CPS B experiencing EM *vs.* patients with CPS A (Table S5). Among patients with CPS B, multivariate regression analysis revealed ECOG ≥2, high

Table 3. Multivariable analysis of risk factors for early mortality in patients treated with atezo/bev.

Variable	Multivariable odds ratio	95% CI	*p* value
CPS B (CPS A as reference)	3.956	1.952–8.068	**0.0001**
Macrovascular invasion	1.411	0.7025–2.844	0.3314
ECOG PS ≥2	1.452	0.4751–4.039	0.4904
Platelets (10^9/L)	1.001	0.9983–1.004	0.4334
CRP (mg/dl)	1.090	1.010–1.181	**0.0258**

Risk factors for early mortality in patients treated with atezo/bev. Odd's ratio for early mortality risk was assessed by multivariable logistic regression. *p* <0.05 was considered statistically significant. CPS, Child–Pugh score; ECOG PS, Eastern Cooperative Oncology Group Performance Status.
Bold font indicate significant *p* values.

creatinine levels, and CPS B9 as independent predictors of early death (Tables S8 and S9). Among patients with preserved liver function with CPS A, ALBI grade provided a granular stratification of EM risk (Tables S6 and S7). ALBI grade 3 was strongly associated with EM in multivariate analysis (OR 12.54, p = 0.0123), while ALBI 2 was directionally consistent (OR 4.382) and borderline not significant (p = 0.056). Hemoglobin also emerged as an independent predictor of EM in this model (OR 0.7957, p = 0.0488).

The main causes of early death among the 50 patients experiencing EM were HCC progression (n = 15, 29.4%), and hepatic decompensation (n = 14, 27.5%) (Fig. S3). Two patients (3.9%) died from bleeding, four (7.8%) from infection, three from cardiac events (5.9%), and two from other causes (n = 1 dermatologic event, n = 1 duodenal ulcer perforation). In one patient, two death causes were recorded: infection and HCC progression. Death cause remained unclear for 11 patients (21.6%). Two deaths were potentially related to atezolizumab (n = 1 dermatologic event, n = 1 myocarditis), five to bevacizumab (n = 1 variceal bleeding, n = 2 other bleeding, n = 1 cardiac event, n = 1 duodenal ulcer perforation), and one to both drugs (hepatic decompensation). Patients dying from hepatic decompensation exhibited higher frequencies of markers associated with impaired liver function at baseline, including elevated CPS, ALBI grades, a higher prevalence of HE, lower albumin and higher bilirubin levels (Table S3). In contrast, patients who died from tumor progression showed higher AFP values and a greater prevalence of extrahepatic spread. Both patients dying from hepatic decompensation and patients dying as a result of HCC progression showed a worsening of liver function parameters such as bilirubin and albumin. However, this was more pronounced for patients with hepatic decompensation as compared with those with HCC progression (Fig. S8). For other death causes, dynamic deterioration of liver function could not be observed.

PBMC immunophenotyping

Since alterations in serum CRP levels in multivariate and the numbers of leukocytes in univariate analysis were significantly associated with EM, we aimed to comprehensively characterize the frequencies and functional status of immune cells before atezo/bev initiation to identify potential immunological biomarkers of EM risk. We performed in-depth immune phenotyping using FACS-based analysis of baseline PBMC in eight patients with EM and 32 who were Non-EM. Representative gating strategies are provided in Fig. S4. Baseline characteristics of our translational cohort were balanced regarding demographics, HCC etiology, and tumor-associated factors, whereas liver function was poorer in patients with EM compared with those who were Non-EM (Table S4). We observed significantly decreased frequencies of lymphocytes among PBMC in patients with EM compared with those who were Non-EM (Fig. 3A). Within the lymphocytic compartment, there was neither a significant difference in the abundance of T cells or B cells, nor a significant alteration in the ratio of cluster of differentiation (CD)4 and CD8 T cells, respectively, between EM and Non-EM groups (Fig. S5A). Regarding cells assigned to innate immunity, there was no significant difference in the frequencies of monocytes and pan dendritic cells (DCs), whereas the frequency of $CD11c^+CD14^+$ monocyte-derived DC (moDC) was significantly increased in the Non-EM group compared with the EM group (Fig. 3B). By analyzing the differentiation status of T cells in peripheral blood, we could demonstrate that $CD4^+$ effector T cells (Teff) were significantly more prominent in patients who were Non-EM compared with those with EM (Fig. 3C). Additionally, the ratio of $CD4^+$ Teff and regulatory T cells (Tregs) was significantly increased in the Non-EM compared with the EM cohort (Fig. 3D). There was no significant difference in the abundance of naïve T cells (Tn), central-memory T cells (Tcm) or effector-memory T cells (Tem) in $CD4^+$ T cells as well as no difference in T cell phenotypes of $CD8^+$ T cells between EM and Non-EM groups (Fig. 3C and Fig. S5B).

Besides determining the frequencies of diverse cell subpopulations, we also aimed to analyze the functional status of these cells in a comprehensive manner. Therefore, we stained T cells for the major activation markers CD25, CD44, Granzyme B (GrzB) and inducible T cell co-stimulator (ICOS) as well as for the inhibitory markers Cytotoxic T lymphocyte associated protein 4 (CTLA-4), Lymphocyte-activation gene 3 (LAG-3), T cell immunoreceptor with Ig and ITIM domains (TIGIT), T cell immunoglobulin and mucin domain-containing-3 (TIM-3) and Programmed death-ligand 1 (PD-L1). Overall, T cell functionality appeared similar in EM and Non-EM groups, as no significant differences could be detected in the expression of CD25, GrzB, ICOS, CTLA-4, LAG-3, TIGIT and TIM-3, neither in $CD4^+$ nor in $CD8^+$ T cells (Fig. S5C and D). However, we observed significantly increased expression levels of the activation marker CD44 on $CD4^+$ T cells, and the immunosuppressive molecule PD-L1 on $CD8^+$ T cells of patients with EM compared with those with Non-EM (Fig. 3E and F). In contrast, CD44 expression on $CD8^+$ T cells and PD-L1 expression on $CD4^+$ T cells was comparable in EM and Non-EM groups (Fig. 3E and F). Additionally, we could detect increased expression of CD44 on $CD4^+$ Treg in patients with EM compared with patients who were Non-EM (Fig. 3G). Regarding the functionality of antigen-presenting cells, we analyzed B cells, DC, moDCs, and monocytes regarding their expression of the co-stimulatory markers CD80, CD86 and Human Leukocyte Antigen – DR isotype (HLA-DR) as well as the co-inhibitory molecule PD-L1. No significant alterations could be detected in the expression of functional markers in B cells (Fig. S5E), DCs (Fig. S5F), moDCs (Fig. S5G) and monocytes (Fig. S5H). Heat maps providing an overview of the immunophenotypes observed within our analysis are displayed in Fig. S6. To address the contribution of HCC etiology to the immunophenotypical changes associated with EM in our cohort, we re-analyzed all significant changes between EM and Non-EM in patients with non-viral HCC (Fig. S7). Seven patients with EM and 23 with Non-EM remained to be analyzed in this subgroup. Here, lymphocyte frequency was lower for EM (Fig. S7A). Additionally, the frequency of moDC was also increased for patients with non-viral HCC who were Non-EM (Fig. S7B). The expression of CD44 on $CD4^+$ T cells and $CD4^+$ Treg persisted to be significantly higher in patients with EM compared with Non-EM in this subgroup (Fig. S7E and G). PD-L1 expression levels, however, on $CD4^+$ and $CD8^+$ T cells were not associated with EM in patients with non-viral HCC (Fig. S7F).

Furthermore, to exclude that alterations in immune cell composition and functional status were attributed to

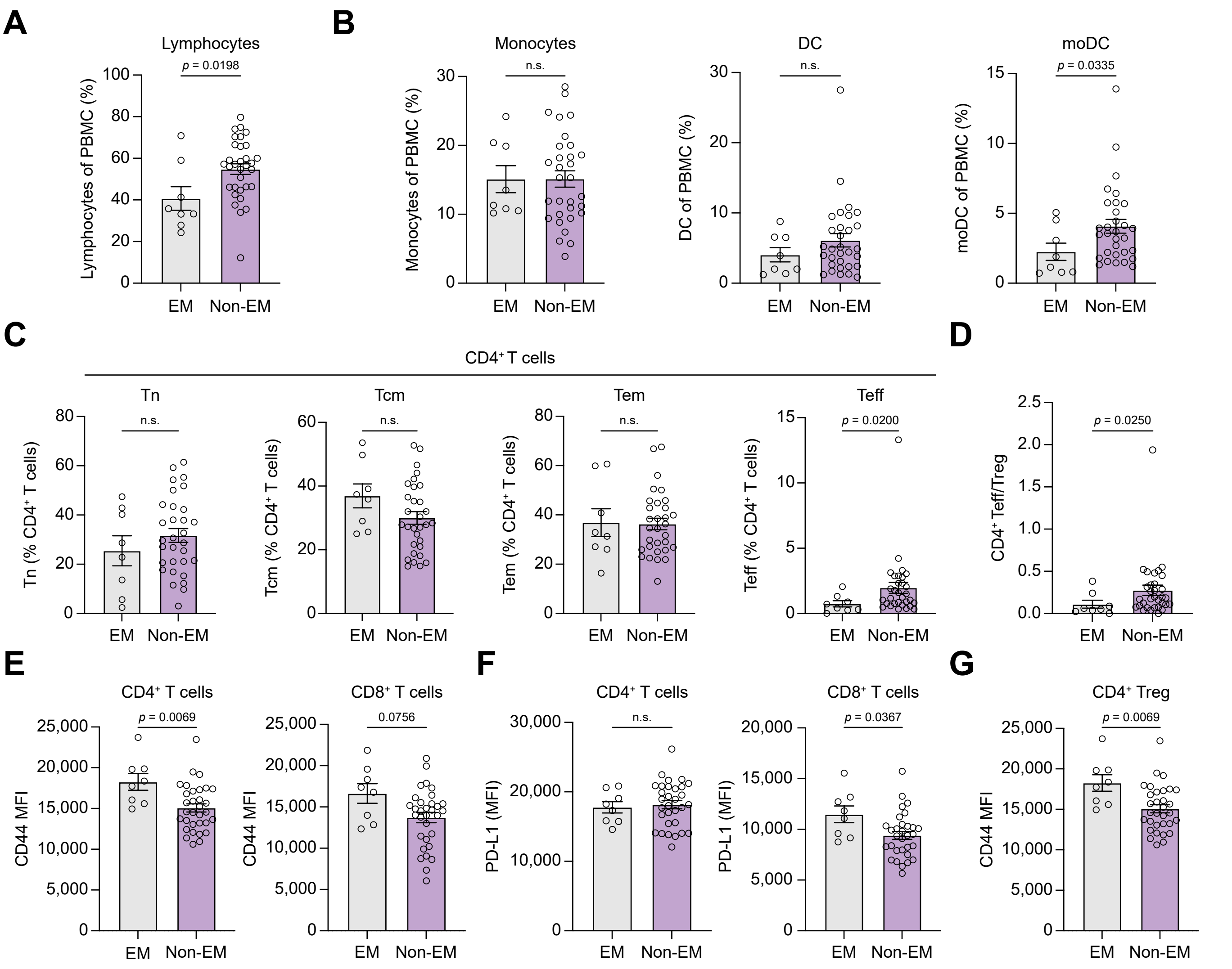

Fig. 3. Immunophenotyping. PBMC of Atezo/bev-treated patients with HCC (EM: n = 8; Non-EM: n = 32) were analyzed via flow cytometry. (A, B) Frequencies of lymphocytes (A) and monocytes, DC and moDC (B) were determined. (C) Distribution of T cell phenotypes (Tn, naïve T cells; Tcm, central-memory T cells; Tem, effector-memory T cells; Teff, effector T cells) in $CD4^+$ T cells was determined. (D) Ratio of $CD4^+$ Teff to Treg was analyzed. (E, F) Expression of CD44 (E) and PD-L1 (F) on $CD4^+$ and $CD8^+$ T cells is displayed as mean fluorescent intensity (MFI). (G) CD44 MFI on $CD4^+$ Treg is shown. Statistical analysis was performed using the Mann–Whitney *U* test. EM, early mortality; DC, dendritic cells; moDC, monocyte-derived DC; Treg, regulatory T cell; MFI, mean fluorescent intensity.

differences in liver function between patients with EM and Non-EM, we re-assessed all significant changes in patients with EM and Non-EM with preserved liver function, as evidenced by CPS A. From the whole biomarker analysis cohort, three patients with EM and 28 patients with Non-EM remained to be analyzed in this subgroup analysis. Here, lymphocyte frequency persisted to be significantly decreased in patients with EM compared with Non-EM (Fig. 4A). Frequencies of moDC and $CD4^+$ Teff, and the $CD4^+$ Teff/Treg ratio differed not statistically significantly between patients with EM and Non-EM when we adjusted for liver function (Fig. 4B–D). Additionally, differences in CD44 expression on $CD4^+$ T cells between patients with EM and Non-EM also lost statistical significance in this subgroup analysis (Fig. 4E). However, increased CD44 expression on Treg remained a significant biomarker of EM, also when adjusted to liver function (Fig. 4F). In contrast, increased PD-L1 expression on $CD8^+$ T cells of patients with EM was not statistically significant in CPS A patients (Fig. 4G).

Discussion

This large, multicenter cohort study evidences an important risk for EM in patients with unresectable HCC treated with atezo/bev in a real-world setting. Significant risk factors for EM involved impaired liver function and alterations in systemic immunity, but not reduced performance status, age, or characteristics of advanced cancer such as extrahepatic spread, macrovascular invasion, and elevated AFP.

This work is the first to explicitly report EM rates for patients with HCC treated with atezo/bev. The 90-day mortality rate was 15.8% (50/317), considerably higher than the ~5% rate seen in the Imbrave150 trial.[4,24] Hepatic decompensation and HCC progression represented the main death causes in our cohort. In contrast, treatment-related toxicity was not identified as major cause of EM. The discrepancy of EM rates reported in our real-world study and the IMbrave150 trial probably reflects the stringent trial eligibility criteria, which do not fully represent the typical patient population in routine

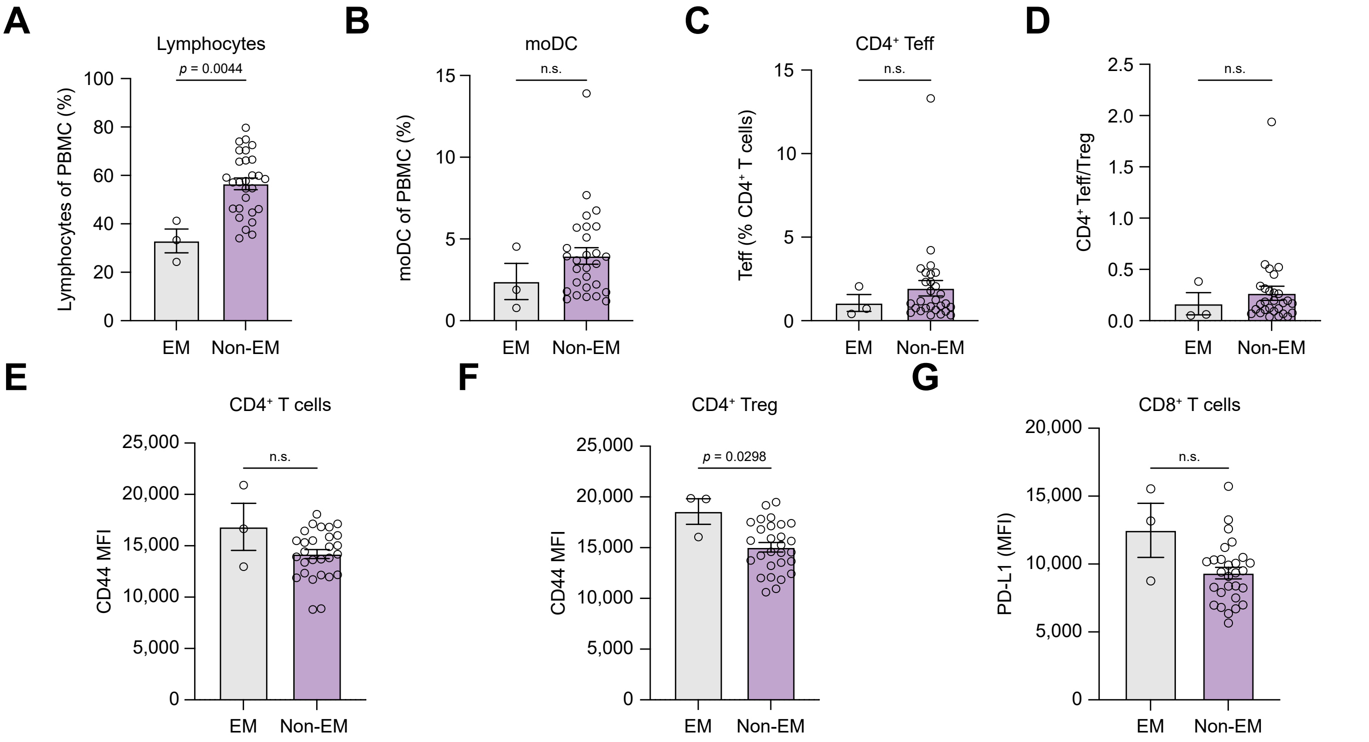

Fig. 4. Immunophenotyping. Subgroup analysis PBMC of Atezo/bev-treated patients with HCC with preserved liver function according to CPS A (EM: n = 3; Non-EM: n = 28) were analyzed via flow cytometry. (A, B) Frequencies of lymphocytes (A) moDC (B) were determined. (C) Frequency of $CD4^+$ effector T cells (Teff) among $CD4^+$ T cells was determined. (D) Ratio of $CD4^+$ Teff to Treg was analyzed. (E, F) Expression of CD44 on $CD4^+$ T cells (E) and Treg (F) is displayed as mean fluorescent intensity (MFI). (G) PD-L1 MFI on $CD8^+$ T cells is shown. Statistical analysis was performed using the Mann–Whitney *U* test. EM, early mortality; MFI, mean fluorescent intensity; moDC, monocyte-derived DC; Treg, regulatory T cell.

clinical practice. In this context, a large proportion of patients in this large, European cohort (64.7%) did not meet the eligibility criteria for the IMbrave150 trial, emphasizing the need for high-quality real-world data to bridge the gap between clinical trials and clinical practice to inform clinical decision-making.

The strongest predictor of 90-day mortality was advanced liver disease in univariate analysis, as reflected by surrogates of impaired liver function such as CPS B (p <0.0001), ALBI grade 2/3 (p = 0.026, p <0.0001), high MELD score (p <0.0001), high bilirubin (p <0.0001), and low albumin (p = 0.003). CPS B remained significant risk factors after adjusting for other variables (p = 0.0001). These factors were also significantly associated with the risk of death within the first 60 days of atezo/bev therapy (CPS B: p <0.001). Although ECOG was associated with EM in univariate analysis, it did not retain significance in the multivariate model. This suggests that the influence of performance status on EM is mediated by other factors included in the multivariate analysis, especially liver function and CRP and does not independently distinguish between EM and Non-EM.

The treatment of patients with HCC and reduced liver function is a daily challenge to hepato-oncologists. About one-third of patients with HCC patients have moderate-to-severe liver disease.[25–28] Impaired liver function often limits the use of local or locoregional therapies,[29–31] and advanced tumor characteristics alongside organ shortage preclude many from liver transplantation, which represents a cure for both cancer and cirrhosis. All currently approved systemic therapies for HCC were exclusively studied in patients with CPS A in the pivotal trials.[3] Faced with this therapeutic dilemma, palliative care is frequently the only option for those with advanced cancer and impaired liver function, yielding a median survival of 4 months.[32,33] The remarkable oncological efficacy of ICI-based therapies such as atezo/bev in HCC has raised hopes as to whether the benefit derived from ICI could extend to patients with impaired liver function. Our study clearly shows that impaired liver function significantly increases the risk of dying within the first 3 months of atezo/bev treatment, with an OR of 3.96 for CPS B (p = 0.0001) in multivariable analysis. Among CPS B patients, those with CPS B9, a poor performance status of ECOG ≥2 and high creatinine levels had a particularly high risk of early death. These variables were not significant predictors of EM in individuals with CPS A, where ALBI grade 3 emerged as a strong predictor of early death. These findings emphasize the need for refined risk stratification approaches within patients with impaired liver function.

Limited oncological outcomes in patients with CPS B treated with atezo/bev were shown in previous retrospective studies[14,16,17,23,34–36] and summarized in a recent meta-analysis.[5] mOS was 6.8 months, which is considerably lower compared with 19.4 months reported in the Imbrave150 trial.[4,24] Patients with CPS B suffered from more treatment-related toxicity with a higher rate of grade ≥3 adverse events of 26.8% *vs.* 11.8% in CPS A. Our findings underscore the necessity of maintaining and stabilizing liver function in

patients with HCC to reduce EM risk upon atezo/bev treatment. Early transition from locoregional to systemic treatment could mitigate the collateral damage from repeated transarterial chemoembolization or radioembolization.[10,37] Successful management of underlying etiology in liver cirrhosis through sustained abstinence in alcohol-related liver diseases and cure or long-term suppression in chronic hepatitis B/C infection can lead to the resolution of hepatic decompensation.[38–41] Atezo/bev therapy can be safely administered to patients with active viral hepatitis, and viral clearance is not necessary before initiating ICI therapy in the advanced setting.[42,43] However, studies have shown that effective antiviral therapy is associated with a reduced risk of decompensation in patients with HCC treated with atezo/bev, suggesting a protective effect.[44]

Inflammatory conditions and immune dysbalance are frequent in cancer and can promote tumor progression, invasion, metastasis, and angiogenesis.[45] In the context of HCC, the complex composition of the tumor immune microenvironment as well as the abundance of certain immune cell subsets in blood and ascites have been linked to patient prognosis and response to immunotherapy.[46–49] Patients experiencing EM had a significantly higher CRP in multivariate analysis (p = 0.026). Inflammatory cytokines such as CRP can suppress antitumor immunity[50] and high CRP is a predictor for worse oncological outcomes in patients with HCC treated with immunotherapy.[19] To address immune alterations mediated by inflammatory conditions, we performed immunophenotyping of baseline patients' PBMCs, which revealed decreased frequencies of lymphocytes and $CD4^+$ Teff, reduced ratio of $CD4^+$ Teff to Treg, and increased expression of the activation marker CD44 on particularly Treg to be significantly associated with EM. Subgroup analysis in patients with preserved liver function revealed that these differences were largely driven by poor liver function. Interestingly, these differences are also associated with decreased antitumor response of ICI therapy. In this context, lower numbers of peripheral blood lymphocytes have been associated with poor outcomes in cancer patients treated with immune checkpoint inhibitors.[51–53] Additionally, regarding Treg, increased CD44 expression has been attributed to enhanced suppressive functionality and persistence.[54,55] Although the frequency of intratumoral and peripheral Treg has already been linked to patient prognosis, the impact of Treg functionality on the prognosis of patients during checkpoint-inhibitor therapy is not well understood to date. However, high CD44 Treg expression representing a highly immunosuppressive Treg phenotype might also represent a detrimental factor for checkpoint-inhibitor response. Additionally, we observed enhanced expression of the immunosuppressive molecule PD-L1 on $CD8^+$ T cells of patients with EM, which has also been linked suppressive function of $CD8^+$ T cells, and to resistance to checkpoint inhibitors.[56,57] Altogether, we conclude that outcomes for patients with HCC with impaired liver function may be constrained both by (1) their increased independent risk of early death due to hepatic decompensation, and (2) an immune profile less capable of mounting a robust anticancer response upon ICI therapy.

This study has limitations. Because of its retrospective nature, some clinical and laboratory parameters were missing in small subsets of patients (Table 1). Second, patients were recruited from tertiary hospitals with high expertise in the management of HCC, which might underestimate the rate of EM. Nevertheless, the extensive sample size of this study allowed for a thorough multivariable regression analysis to identify clinical and biochemical EM risk factors. Third, the subgroup analysis of immunophenotyping in patients with preserved liver function had a limited sample size with three patients experiencing EM. Larger studies are needed to investigate the influence of circulating PBMCs on mortality in patients with preserved liver function. Fourth, classification of death causes was done without autopsy based on the available clinical, laboratory, and imaging data, which might have led to misclassifications.

In summary, this large multicenter study is the first to draw attention to early death as a significant and frequent outcome for patients treated with atezo/bev in the real world. The identified risk factors can help clinicians identify patients at high risk of EM, enabling timely and informed decision-making regarding end-of-life care and palliative treatment. Addressing these issues proactively is a fundamental responsibility of the treating physician.

Conclusions

EM is frequent in patients with HCC treated with atezo/bev. Significant risk factors for dying early involved impaired liver function, elevated biomarkers of inflammation, and alterations of systemic immunity, but not reduced performance status, age, or characteristics of advanced cancer such as extrahepatic spread, macrovascular invasion, or elevated AFP.

Affiliations

[1]*Department of Medicine II, University Hospital, LMU Munich, Munich, Germany;* [2]*Division of Clinical Pharmacology, Department of Medicine II, University Hospital, LMU Munich, Munich, Germany;* [3]*Partner site – German Alliance for Liver Cancer (GALC), Heidelberg, Germany;* [4]*Department of Gastroenterology and Hepatology, University Hospital Essen, University of Duisburg-Essen, Essen, Germany;* [5]*TUM School of Medicine and Health, Department of Clinical Medicine – Clinical Department for Internal Medicine II, University Medical Center, Technical University of Munich, Germany;* [6]*Division of Gastroenterology and Hepatology, Department of Medicine III, Medical University of Vienna, Vienna, Austria;* [7]*Department of Gastroenterology, Hepatology and Infectious Diseases, Otto-von-Guericke University Hospital Magdeburg, Magdeburg, Germany;* [8]*Department of Medicine I, University Medical Center of the Johannes-Gutenberg University Mainz, Mainz, Germany;* [9]*Department of Medicine III, University Hospital, LMU Munich, Munich, Germany;* [10]*Pharmacy, University Hospital, LMU Munich, Munich, Germany;* [11]*Division of Hepatology, Department of Medicine II, University Hospital Würzburg, Würzburg, Germany;* [12]*Department of Radiology, University Hospital, LMU Munich, Munich, Germany;* [13]*Bavarian Cancer Research Center (BZKF), Erlangen, Germany*

Abbreviations

AFP, alpha-fetoprotein; ALBI, albumin–bilirubin; Atezo/bev, atezolizumab plus bevacizumab; BCLC, Barcelona Clinic liver cancer; CD, cluster of differentiation; CPS, Child–Pugh score; CR, complete response; CRP, C-reactive protein; CTLA-4, Cytotoxic T lymphocyte associated protein 4; DC, dendritic cell; EASL, European Association for the Study of the Liver; ECOG, Eastern Cooperative Oncology Group; EM, early mortality; FCS, fetal calf serum; GrzB, granzyme B; HCC, hepatocellular carcinoma; HE, hepatic encephalopathy; HLA-DR, human leukocyte antigen – DR isotype; ICI, immune checkpoint inhibitor; ICOS, inducible T cell co-stimulator; INR, international normalized ratio; LAG-3, Lymphocyte-activation gene 3; MELD, model for end-stage liver disease; moDC, monocyte-derived dendritic cells; mOS, median overall survival; mPFS, median progression-free survival; NSBB, non-selective beta-blocker; OR, odds ratio;

ORR, overall response rate; OS, overall survival; PBMC, peripheral blood mononuclear cell; PD, progressive disease; PD-L1, Programmed death-ligand 1; PFS, progression-free survival; PR, partial response; SD, stable disease; Tcm, central-memory T cell; Teff, effector T cell; Tem, effector-memory T cell; TIGIT, T cell immunoreceptor with Ig and ITIM domains; TIM-3, T cell immunoglobulin and mucin domain-containing-3; Tn, naïve T cell; Treg, regulatory T cell; VEGF, vascular endothelial growth factor.

Financial support

This study was initiated by the IMMUreal study group. The protocol was endorsed and supported by the German Alliance for Liver Cancer (GALC). NBK was supported by the Bavarian Cancer Research Center, FöFoLe of LMU Munich funding program (1122), the ESMO Research Fellowship, and the German Cancer Consortium. IP was supported by the Else Kröner-Fresenius-Stiftung (2025_EKEA.16 and IOLIN), the Bavarian Cancer Research Center, the Medical Faculty of the LMU Munich (intramural funding) and the Novartis Foundation (InCa prize).The funding bodies had no role in the design of the study; the collection, analysis, or interpretation of data; or the writing of the manuscript.

Conflicts of interest

IP received reimbursement of travel expenses from Roche. NBK has received reimbursement of meeting attendance fees and travel expenses from Eisai and lecture honorarium from Falk and Astra Zeneca. FPR has received honoraria for lectures and travel support from the Falk Foundation, Gilead, Ipsen and Novartis. AP has received honoraria for lectures from the Falk Foundation, and travel support from Roche. UE has received honoraria for lectures from AstraZeneca, the Falk Foundation, IPSEN, Novartis and Roche, and travel support from AstraZeneca and Biotest. She has served as advisory board or steering committee member to AstraZeneca, Bayer, Eisai, and MSD. KBo has received honoraria for lectures from IPSEN. KBe received a research grant from Roche Pharma AG. MP served as a speaker and/or consultant and/or advisory board member for Astra Zeneca, Bayer, Bristol-Myers Squibb, EISAI, Ipsen, Lilly, MSD, and Roche, and received travel support from Bayer, Bristol-Myers Squibb, Ipsen, and Roche. BS received grant support from AstraZeneca and EISAI, speaker honoraria from EISAI as well as travel support from AbbVie, AstraZeneca, Ipsen and Gilead. MR has received honoraria for lectures and travel support from Gilead. OÖ received honorarium from Bayer. MV has received honoraria for speaker, consultancy, and advisory role from Amgen, AstraZeneca, Bayer, BMS, Eisai, Ipsen, Lilly, Merck Serono, MSD, Nordic Pharma, Roche, Servier and Sirtex. SJG has received travel expenses from Gilead and Ipsen. LSJ has received honoraria for lectures from AstraZeneca, the Falk Foundation, AbbVie and Boston scientific and travel support from Biotest and AbbVie. She has served as advisory board member to AstraZeneca. FF has received honoraria for lectures from AstraZeneca, MSD, Pfizer, Roche and reimbursement of meeting attendance fees and travel expenses from Merck KGaA and Servier. He has served as advisory board or steering committee member to AstraZeneca, BMS, EISAI and Roche. AG is advisory board or steering committee member to AbbVie, Advanz, Albireo, Alexion, AstraZeneca, Bayer, Boehringer, BMS, EISAI, Falk, Gilead, Heel, Intercept, Ipsen, Merz, MSD, Novartis, Pfizer, Roche, Sanofi-Aventis and speaker for AbbVie, Adavanz, Alexion, BMS, Burgerstein, CSL Behring, Falk, Gilead, Intercept, Merz, MSD, Novartis, Novo Nordisk, Orphalan, Roche; he received research support from Intercept and Falk (NAFLD CSG), Novartis. ENDT has served as a paid consultant for AstraZeneca, Bayer, BMS, Eisai, Eli Lilly & Co, Pfizer, IPSEN, and Roche. He has received reimbursement of meeting attendance fees and travel expenses from Arqule, AstraZeneca, BMS, Bayer, Celsion, and Roche, and lecture honoraria from BMS and Falk. In addition, he has received third-party funding for scientific research from Arqule, AstraZeneca, BMS, Bayer, Eli Lilly, and Roche. AT served as consultant for Astellas Gene Therapies Inc. and Roche Pharma AG. JR received grants and personal fees from Sirtex and Bayer. All the other authors have no conflicts of interest to declare.

Please refer to the accompanying ICMJE disclosure forms for further details.

Authors' contributions

Conceptualization: IP, ENDT, SJG, AG, FPR, NBK. Data curation: all authors. Methodology: NBK, FPR, IP, OÖ, SJG, MA-F. Project administration: NBK, FPR, IP. Resources: NBK, FPR, IP, AP, DA, MA-F, JR, JM, AG, ENDT. Software: NBK, FPR, IP. Supervision: DA, KBe, CS, CML, JR, JM, AG, ENDT. Writing – original draft: IP, NBK, FPR, ENDT, SE. Writing – review and editing: all authors.

Data availability

All data being analyzed as part of this study are included in this manuscript and the supplementary materials. Further inquiries can be sent to the corresponding author.

Acknowledgements

We would like to thank all patients and their families, as well as the staff of participating institutions. We would like to thank Cheryl Gray and Karl Schondorff for excellent support.

Supplementary data

Supplementary data to this article can be found online at https://doi.org/10.1016/j.jhepr.2025.101513.

References

[1] D'Amico G, Bernardi M, Angeli P. Towards a new definition of decompensated cirrhosis. J Hepatol 2022 Jan;76(1):202–207.

[2] Llovet JM, Kelley RK, Villanueva A, et al. Hepatocellular carcinoma. Nat Rev Dis Primers 2021;7:6.

[3] Reiter FP, Ben Khaled N, Ye L, et al. Advances in pharmacotherapy of hepatocellular carcinoma: a state of the art review. Dig Dis 2021;40:565–580.

[4] Finn RS, Qin S, Ikeda M, et al. Atezolizumab plus bevacizumab in unresectable hepatocellular carcinoma. New Engl J Med 2020;382:1894–1905.

[5] Pasta A, Calabrese F, Jaffe A, et al. Safety and efficacy of atezolizumab/bevacizumab in patients with hepatocellular carcinoma and impaired liver function: a systematic review and meta-analysis. Liver Cancer 2023;13:227–237.

[6] Viscardi G, Tralongo AC, Massari F, et al. Comparative assessment of early mortality risk upon immune checkpoint inhibitors alone or in combination with other agents across solid malignancies: a systematic review and meta-analysis. Eur J Cancer 2022;177:175–185.

[7] Raphael J, Richard L, Lam M, et al. Early mortality in patients with cancer treated with immune checkpoint inhibitors in routine practice. J Natl Cancer Inst 2023;115:949–961.

[8] Gibson A, Meyers D, Dean M, et al. P16.03 early mortality associated factors with immune checkpoint inhibition in real-world Canadian NSCLC patients. J Thorac Oncol 2021;16:S348.

[9] Takeuchi E, Kondo K, Okano Y, et al. Early mortality factors in immune checkpoint inhibitor monotherapy for advanced or metastatic non-small cell lung cancer. J Cancer Res Clin Oncol 2023;149:3139–3147.

[10] European Association for the Study of the Liver. EASL clinical practice guidelines: management of hepatocellular carcinoma. J Hepatol 2018;69:182–236.

[11] Kamath PS, Wiesner RH, Malinchoc M, et al. A model to predict survival in patients with end-stage liver disease. Hepatology 2001;33:464–470.

[12] Giessen C, Graeven U, Laubender RP, et al. Prognostic factors for 60-day mortality in first-line treatment of metastatic colorectal cancer (mCRC): individual patient analysis of four randomised, controlled trials by the AIO colorectal cancer study group. Ann Oncol 2013;24:3051–3055.

[13] von Elm E, Altman DG, Egger M, et al. The Strengthening the Reporting of Observational Studies in Epidemiology (STROBE) statement: guidelines for reporting observational studies. J Clin Epidemiol 2008;61:344–349.

[14] de Castro T, Jochheim LS, Bathon M, et al. Atezolizumab and bevacizumab in patients with advanced hepatocellular carcinoma with impaired liver function and prior systemic therapy: a real-world experience. Ther Adv Med Oncol 2022;14:17588359221080298.

[15] Sinner F, Pinter M, Scheiner B, et al. Atezolizumab plus bevacizumab in patients with advanced and progressing hepatocellular carcinoma: retrospective multicenter experience. Cancers (Basel) 2022;14:5966.

[16] D'Alessio A, Fulgenzi CAM, Nishida N, et al. Preliminary evidence of safety and tolerability of atezolizumab plus bevacizumab in patients with hepatocellular carcinoma and Child-Pugh A and B cirrhosis: a real-world study. Hepatology 2022;76:1000–1012.

[17] Himmelsbach V, Pinter M, Scheiner B, et al. Efficacy and safety of atezolizumab and bevacizumab in the real-world treatment of advanced hepatocellular carcinoma: experience from four tertiary centers. Cancers (Basel) 2022;14:1722.

[18] Fulgenzi CAM, Cheon J, D'Alessio A, et al. Reproducible safety and efficacy of atezolizumab plus bevacizumab for HCC in clinical practice: results of the AB-real study. Eur J Cancer 2022;175:204–213.

[19] Scheiner B, Pomej K, Kirstein MM, et al. Prognosis of patients with hepatocellular carcinoma treated with immunotherapy – development and validation of the CRAFITY score. J Hepatol 2022;76:353–363.

[20] Greten TF, Malek NP, Schmidt S, et al. [Diagnosis of and therapy for hepatocellular carcinoma]. Z Gastroenterol 2013;51:1269–1326.

[21] Haanen J, Obeid M, Spain L, et al. Management of toxicities from immunotherapy: ESMO Clinical Practice Guideline for diagnosis, treatment and follow-up. Ann Oncol 2022;33:1217–1238.

[22] Brahmer JR, Lacchetti C, Schneider BJ, et al. Management of immune-related adverse events in patients treated with immune checkpoint inhibitor therapy: American Society of Clinical Oncology Clinical Practice Guideline. J Clin Oncol 2018;36:1714–1768.
[23] Cheon J, Kim H, Kim HS, et al. Atezolizumab plus bevacizumab in patients with Child-Pugh B advanced hepatocellular carcinoma. Ther Adv Med Oncol 2023;15:17588359221148541.
[24] Cheng A-L, Qin S, Ikeda M, et al. Updated efficacy and safety data from IMbrave150: atezolizumab plus bevacizumab vs. sorafenib for unresectable hepatocellular carcinoma. J Hepatol 2022:76862–76873.
[25] Garuti F, Neri A, Avanzato F, et al. The changing scenario of hepatocellular carcinoma in Italy: an update. Liver Int 2021;41:585–597.
[26] Ben Khaled N, Mörtl B, Beier D, et al. Changing treatment landscape associated with improved survival in advanced hepatocellular carcinoma: a nationwide, population-based study. Eur J Cancer 2023;192:113248.
[27] Pugh RNH, Murray-Lyon IM, Dawson JL, et al. Transection of the oesophagus for bleeding oesophageal varices. Br J Surg 2005;60:646–649.
[28] Jeon D, Song G-W, Lee HC, et al. Treatment patterns for hepatocellular carcinoma in patients with Child–Pugh class B and their impact on survival: a Korean nationwide registry study. Liver Int 2022;42:2830–2842.
[29] Peck-Radosavljevic M, Kudo M, Raoul J-L, et al. Outcomes of patients (pts) with hepatocellular carcinoma (HCC) treated with transarterial chemoembolization (TACE): global OPTIMIS final analysis. J Clin Oncol 2018;36 (15 suppl):4018.
[30] Peck-Radosavljevic M, Raoul J-L, Lee HC, et al. OPTIMIS: an international observational study to assess the use of sorafenib after transarterial chemoembolization (TACE) in patients with hepatocellular carcinoma (HCC). J Clin Oncol 2014;32(15 suppl):TPS4155–TPS.
[31] Ben Khaled N, Seidensticker M, Ricke J, et al. Atezolizumab and bevacizumab with transarterial chemoembolization in hepatocellular carcinoma: the DEMAND trial protocol. Future Oncol 2022;18:1423–1435.
[32] Blanc JF, Khemissa F, Bronowicki JP, et al. Phase 2 trial comparing sorafenib, pravastatin, their combination or supportive care in HCC with Child-Pugh B cirrhosis. Hepatol Int 2021;15:93–104.
[33] Reig M, Forner A, Rimola J, et al. BCLC strategy for prognosis prediction and treatment recommendation Barcelona Clinic Liver Cancer (BCLC) staging system. The 2022 update. J Hepatol 2022;76:681–693.
[34] Rimini M, Persano M, Tada T, et al. Survival outcomes from atezolizumab plus bevacizumab versus Lenvatinib in Child Pugh B unresectable hepatocellular carcinoma patients. J Cancer Res Clin Oncol 2023;149. 7565–77.
[35] Sho T, Suda G, Yamamoto Y, et al. Efficacy and effect on liver functional reserve of atezolizumab and bevacizumab for unresectable hepatocellular carcinoma in patients who do not meet eligibility criteria of IMbrave150. Cancers (Basel) 2022;14:3938.
[36] Tanaka T, Hiraoka A, Tada T, et al. Therapeutic efficacy of atezolizumab plus bevacizumab treatment for unresectable hepatocellular carcinoma in patients with Child-Pugh class A or B liver function in real-world clinical practice. Hepatol Res 2022;52:773–783.
[37] Hiraoka A, Kumada T, Kudo M, et al. Hepatic function during repeated TACE procedures and prognosis after introducing sorafenib in patients with unresectable hepatocellular carcinoma: multicenter analysis. Dig Dis 2017;35:602–610.
[38] Hofer BS, Simbrunner B, Hartl L, et al. Hepatic recompensation according to Baveno VII criteria is linked to a significant survival benefit in decompensated alcohol-related cirrhosis. Liver Int 2023;43:2220–2231.
[39] Martini S, Donato MF, Mazzarelli C, et al. The Italian compassionate use of sofosbuvir in HCV patients waitlisted for liver transplantation: a national real-life experience. Liver Int 2018;38:733–741.
[40] Reiberger T, Hofer BS. The Baveno VII concept of cirrhosis recompensation. Dig Liver Dis 2023;55:431–441.
[41] Wang Q, Zhao H, Deng Y, et al. Validation of Baveno VII criteria for recompensation in entecavir-treated patients with hepatitis B-related decompensated cirrhosis. J Hepatol 2022;77:1564–1572.
[42] Zhu X-D, Sun H-C, Shen F, et al. The safety of atezolizumab plus bevacizumab (atezo/bev) treatment in Chinese patients (pts) with hepatocellular carcinoma (HCC) with active hepatitis B virus (HBV) infection: results from the TALENTop study. J Clin Oncol 2024;42(16 suppl):e16168.
[43] Hsu C, Ducreux M, Zhu AX, et al. Hepatic events and viral kinetics in hepatocellular carcinoma patients treated with atezolizumab plus bevacizumab. Liver Cancer 2023;12:44–56.
[44] Celsa C, Cabibbo G, Fulgenzi CAM, et al. Hepatic decompensation is the major driver of mortality in patients with HCC treated with atezolizumab plus bevacizumab: the impact of successful antiviral treatment. Hepatology 2025;81:837–852.
[45] Hanahan D, Weinberg RA. Hallmarks of cancer: the next generation. Cell 2011;144:646–674.
[46] Zhang Q, He Y, Luo N, et al. Landscape and dynamics of single immune cells in hepatocellular carcinoma. Cell 2019;179. 829–45.e20.
[47] Bagaev A, Kotlov N, Nomie K, et al. Conserved pan-cancer microenvironment subtypes predict response to immunotherapy. Cancer Cell 2021;39. 845–65.e7.
[48] Mirshahi F, Aqbi HF, Isbell M, et al. Distinct hepatic immunological patterns are associated with the progression or inhibition of hepatocellular carcinoma. Cell Rep 2022;38:110454.
[49] Donne R, Lujambio A. The liver cancer immune microenvironment: therapeutic implications for hepatocellular carcinoma. Hepatology 2023;77:1773–1796.
[50] Yoshida T, Ichikawa J, Giuroiu I, et al. C reactive protein impairs adaptive immunity in immune cells of patients with melanoma. J Immunother Cancer 2020;8:e000234.
[51] Lee YJ, Park YS, Lee HW, et al. Peripheral lymphocyte count as a surrogate marker of immune checkpoint inhibitor therapy outcomes in patients with non-small-cell lung cancer. Sci Rep 2022;12:626.
[52] Young RB, Panchal H, Ma W, et al. Hospitalized cancer patients with comorbidities and low lymphocyte counts had poor clinical outcomes to immune checkpoint inhibitors. Front Oncol 2022;12:980181.
[53] Ho WJ, Yarchoan M, Hopkins A, et al. Association between pretreatment lymphocyte count and response to PD1 inhibitors in head and neck squamous cell carcinomas. J Immunother Cancer 2018;6:84.
[54] Bollyky PL, Falk BA, Long SA, et al. CD44 costimulation promotes FoxP3+ regulatory T cell persistence and function via production of IL-2, IL-10, and TGF-beta. J Immunol 2009;183:2232–2241.
[55] Liu T, Soong L, Liu G, et al. CD44 expression positively correlates with Foxp3 expression and suppressive function of CD4+ Treg cells. Biol Direct 2009;4:40.
[56] Jacquelot N, Roberti MP, Enot DP, et al. Predictors of responses to immune checkpoint blockade in advanced melanoma. Nat Commun 2017;8:592.
[57] Diskin B, Adam S, Cassini MF, et al. PD-L1 engagement on T cells promotes self-tolerance and suppression of neighboring macrophages and effector T cells in cancer. Nat Immunol 2020;21:442–454.

Keywords: Hepatocellular carcinoma; Immunotherapy; Atezolizumab; Bevacizumab; Early mortality.
Received 12 May 2024; received in revised form 30 June 2025; accepted 1 July 2025; Available online 5 July 2025

Journal of Hepatology, Volume ■

Supplemental information

Early mortality in atezolizumab/bevacizumab for HCC is associated with impaired liver function and alterations of systemic immunity

Ignazio Piseddu, Leonie S. Jochheim, Katrin Boettcher, Bernhard Scheiner, Friedrich Sinner, Simon Johannes Gairing, Matthias Thaler, Stefan Enssle, Monika Karin, Valentina Zarka, Alexander Philipp, Andreas Thalmeier, Jan Gaertig, Lorenz Balcar, Julia Martina Schütte, Julia S. Schneider, Katarina Ondrejkova, Monika Rau, Alexander Weich, David Anz, Karin Berger, Christian Schulz, Christian M. Lange, Osman Öcal, Marianna Alunni-Fabbroni, Jens Ricke, Ursula Ehmer, Marino Venerito, Friedrich Foerster, Matthias Pinter, Andreas Geier, Julia Mayerle, Enrico N. De Toni, Florian P. Reiter, and Najib Ben Khaled

Early mortality in atezolizumab/bevacizumab for HCC is associated with impaired liver function and alterations of systemic immunity

Ignazio Piseddu, Leonie S. Jochheim, Katrin Boettcher, Bernhard Scheiner, Friedrich Sinner, Simon Johannes Gairing, Matthias Thaler, Stefan Enssle, Monika Karin, Valentina Zarka, Alexander Philipp, Andreas Thalmeier, Jan Gaertig, Lorenz Balcar, Julia Martina Schütte, Julia S. Schneider, Katarina Ondrejkova, Monika Rau, Alexander Weich, David Anz, Karin Berger, Christian Schulz, Christian M. Lange, Osman Öcal, Marianna Alunni-Fabbroni, Jens Ricke, Ursula Ehmer, Marino Venerito, Friedrich Foerster, Matthias Pinter, Andreas Geier, Julia Mayerle, Enrico N. De Toni, Florian P. Reiter, Najib Ben Khaled

Table of contents

Supplementary figures……………………………………………………………….2

Supplementary tables………………………………………………………………10

Supplementary figures

Fig. S1. Median overall survival of the early mortality cohort.

Fig. S2. Median progression-free survival of the early mortality cohort.

Fig. S3. Causes of death in the early mortality cohort. Diseases were categorized as liver decompensation, HCC progression, bleeding, infection, cardiac event, thromboembolic event, respiratory event, neurologic event, endocrine toxicity, renal toxicity, other toxicity, or unknown cause. Reasonable causal relationship to atezolizumab, bevacizumab, or both drugs was assessed by the local investigator. In one patient, two death causes were recorded: infection and HCC progression.

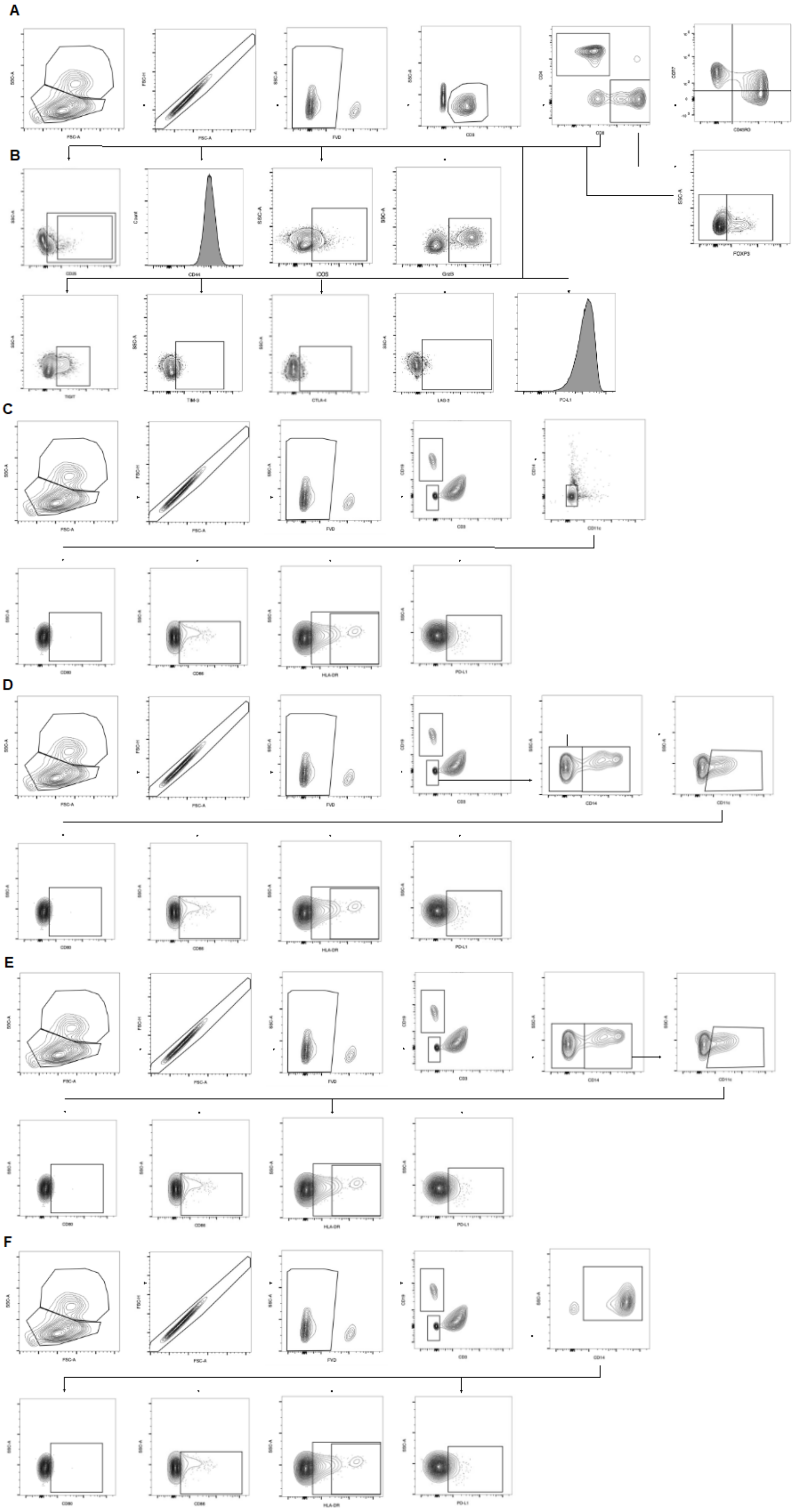

Fig. S4. Gating strategies. Representative gating strategies for (A) T cell phenotype, (B) T cell activation and exhaustion, (C) B cell activity, (D) DC activity, (E) moDC activity and (F) monocyte activity are shown.

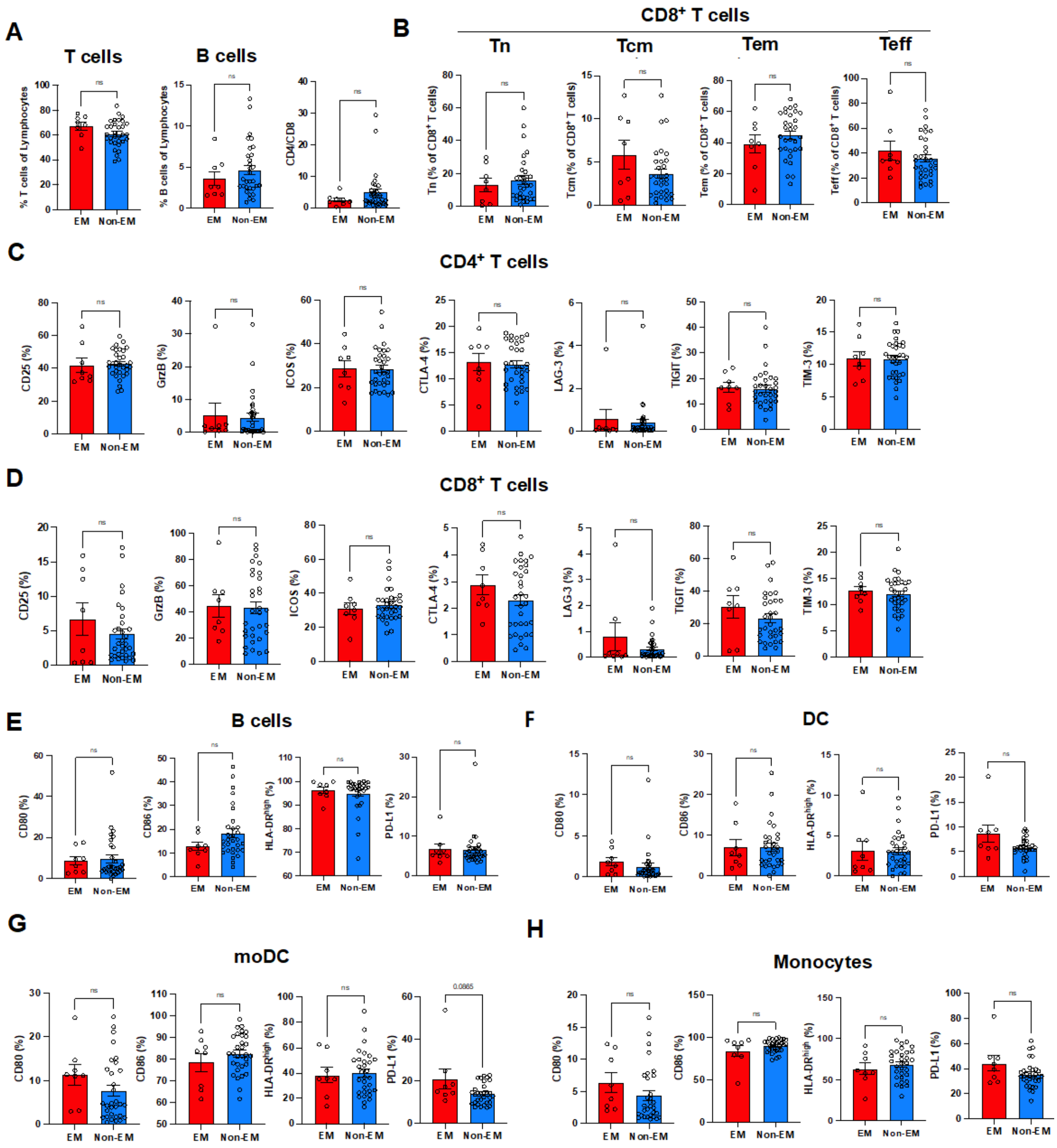

Fig. S5. Immunophenotyping. PBMC of Atezo/bev-treated HCC patients (EM: n = 8; Non-EM: n = 32) were analysed via flow cytometry. (A) Frequencies of CD4+ and CD8+ T cells within T cells as well as CD4 to CD8 ratio are shown. (B) Distribution of T cell phenotypes (Tn = naive T cells, Tcm = central memory T cells, Tem= effector memory T cells, Teff= effector T cells) in CD8+ T cells was determined. (C + D) Expression of the activation markers CD25, Granzyme B (GrzB) and ICOS as well as the exhaustion markers CTLA-4, LAG-3, TIGIT and TIM-3 are displayed in CD4+ (C) and CD8+ T cells (D). (E-H) Expression of the activation markers CD80, CD86 and HLA-DR as well as the inhibitory molecule PD-L1 were determined on (E) B cells, (F) DC, (G) moDC and (H) monocytes. Abbreviations: EM, early mortality; GrzB, Granzyme B; DC, dendritic cells; moDC, monocyte-derived DC.

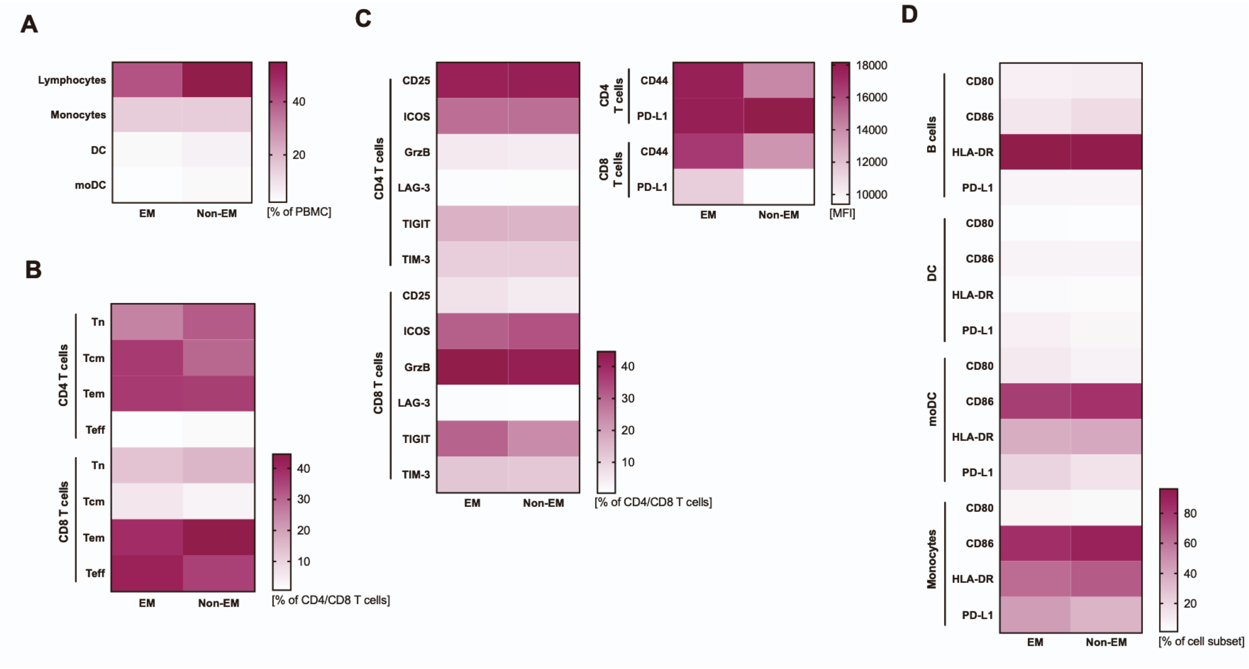

Fig. S6. Overview heat maps of PBMC immunophenotyping. PBMC of Atezo/bev-treated HCC patients (EM: n = 8; Non-EM: n = 32) were analysed via flow cytometry. (A) Frequencies of indicated immune cell subsets among whole PBMC are shown. (B) Distribution of T cell phenotypes (Tn = naive T cells, Tcm = central memory T cells, Tem= effector memory T cells, Teff= effector T cells) in CD4+ and CD8+ T cells is displayed. (C) Expression of the activation markers CD25, Granzyme B (GrzB) and ICOS as well as the exhaustion markers CTLA-4, LAG-3, TIGIT and TIM-3 are displayed in CD4+ and CD8+ T cells (left). Mean fluorescence intensity (MFI) of CD44 and PD-L1 on CD4+ and CD8+ T cells is shown (right). (D) Expression of the activation markers CD80, CD86 and HLA-DR as well as the inhibitory molecule PD-L1 were determined on B cells, DC, moDC and monocytes. Abbreviations: EM, early mortality; GrzB, Granzyme B; DC, dendritic cells; moDC, monocyte-derived DC.

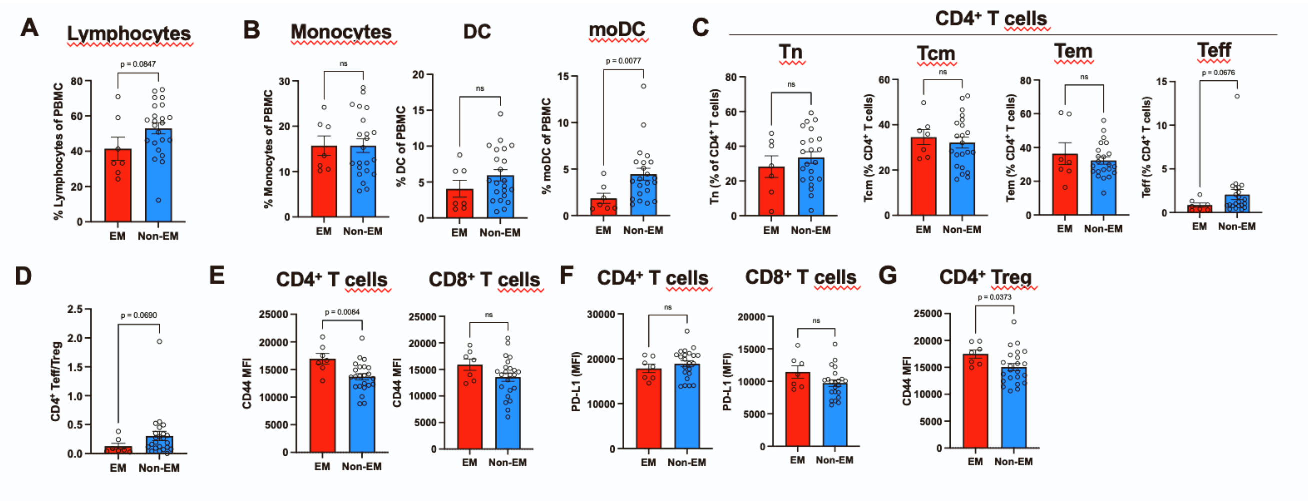

Fig. S7. PBMC of Atezo/bev-treated patients with non-viral HCC (EM: n = 7; Non-EM: n = 23) were analyzed via flow cytometry. (A + B) Frequencies of lymphocytes (A) and monocytes, DC and moDC (B) were determined. (C) Distribution of T cell phenotypes (Tn = naive T cells, Tcm = central memory T cells, Tem= effector memory T cells, Teff= effector T cells) in CD4+ T cells was determined. (D) Ratio of CD4+ Teff to Treg was analyzed. (E + F) Expression of CD44 (E) and PD-L1 (F) on CD4+ and CD8+ T cells is displayed as mean fluorescent intensity (MFI). (G) CD44 MFI on CD4+ Treg is shown. Abbreviations: EM, early mortality; DC, dendritic cells; moDC, monocyte-derived DC; Treg, regulatory T cell; MFI, mean fluorescent intensity

Fig. S8. Changes in liver function from baseline to follow-up visit in patients with HCC treated with atezo/bev and experiencing early mortality due to (A) progressive cancer, (B) hepatic decompensation, or (C) other causes. Baseline consists in the first cycle of atezo/bev. For follow-up values, values from week 6 after treatment start were used, where available. If no week 6 values were available, we included the closest value between week 3 and week 12. Scatter plots depict individual values and mean. Paired t-test was used to compare baseline and follow-up. Abbreviations: ALBI, Albumin-Bilirubin.

Supplementary tables

Table S1. Univariate analysis of risk factors for 60 days early mortality in patients treated with atezo/bev

**Variable**	**Univariate Odd's ratio**	**95% (CI)**	**p-value**
Age	1.01	0.97 to 1.05	0.603
Sex (male)	2.63	0.89 to 11.28	0.121
ECOG PS ≥ 2	3.24	1.1 to 8.4	**0.021**
Liver cirrhosis	0.76	0.35 to 1.78	0.516
Nonviral etiology	0.62	0.27 to 1.48	0.264
CPS B (CPS A as reference)	6.09	2.77 to 13.79	**<0.001**
CPS A6 (A5 as reference)	9.18	2.3 to 61.17	**0.005**
CPS B7 (A5 as reference)	16.95	3.89 to 117.4	**0.001**
CPS B8 (A5 as reference)	27.68	5.44 to 206.1	**0.002**
CPS B9 (A5 as reference)	48.44	9.01 to 378.3	**<0.001**
Ascites, moderate (no ascites as reference)	1.48	0.56 to 3.58	0.399
Ascites, severe (no ascites as reference)	5.45	1.1 to 21.53	**0.02**
Hepatic encephalopathy	1.79	0.09 to 11.02	0.596
BCLC C (A-B as reference)	4.1	1.19 to 25.83	0.058
Extrahepatic spread	1.51	0.7 to 3.34	0.295
Macrovascular invasion	2.3	1.05 to 5.22	**0.04**
Presence of GE varices	1.31	0.6 to 2.87	0.5
Esophageal varices grade II-III (0-I as reference)	0.81	0.23 to 2.21	0.705
History of variceal bleeding	2.73	0.6 to 9.28	0.138
Spleen size	1.05	0.91 to 1.21	0.486
NSBB	0.82	0.31 to 1.9	0.654
Anticoagulation	1.24	0.54 to 2.73	0.595
Antiplatelets	1.75	0.7 to 5.33	0.273
Albumin g/dl	0.1	0.04 to 0.22	**<0.001**
Bilirubin md/gl	2.26	1.58 to 3.36	**<0.001**
ALBI Grade 2 (Grade 1 as reference)	5.12	0.96 to 94.76	0.122
ALBI Grade 3 (Grade 1 as reference)	48	9.16 to 885.6	**<0.001**
INR	2.31	0.5 to 8.05	0.2127
Creatinine mg/dl	1.11	0.54 to 1.73	0.673
Presence of proteinuria	0.76	0.21 to 2.27	0.65
CRP mg/dl	1.16	1.07 to 1.25	**0.002**
AFP ng/ml	1	1.0 to 1.0	0.952

Leucocytes 10^9/L	1.16	1.03 to 1.3	**0.01**
Neutrophils 10^9/L	1.01	0.95 to 1.06	0.533
Platelets 10^9/L	1	1.0 to 1.01	**0.002**
Hemoglobin g/dl	0.83	0.69 to 1.0	**0.046**
MELD score	1.13	1.05 to 1.22	**0.001**
Neutrophils to platelets ratio	1	0.99 to 1.01	0.944

Risk factors for 60 days early mortality in patients treated with atezo/bev. Odd's ratio for early mortality risk was assessed by univariate logistic regression. Abbreviations: ALBI, Albumin-Bilirubin; BCLC, Barcelona Clinic Liver Cancer; CI, confidence interval; CPS, Child-Pugh score; ECOG, Eastern Cooperative Oncology Group Performance Status; GE varices, gastoesophageal varices; MELD, Model of Endstage Liver Disease; NSBB, non-selective beta-blockers.

Table S2. Multivariate analysis of risk factors for 60 days early mortality in patients treated with atezo/bev

Variable	Univariate Odd's ratio	95% (CI)	p-value
CPS B (CPS A as reference)	6.794	2.811 to 17.46	**<0.0001**
CRP mg/dl	1.121	1.031 to 1.225	**0.0065**
Platelets 10^9/L	1.001	0.9981 to 1.004	0.4033

Risk factors for 60 days early mortality in patients treated with atezo/bev. Odd's ratio for early mortality risk was assessed by multivariate logistic regression. Abbreviations: CI, confidence interval; CPS, Child-Pugh score.

Table S3. Baseline characteristics of patients experiencing EM due to progressive disease or hepatic decompensation

Variable	Progressive disease n=15	Hepatic decompensation n=14
**Mean age ± SD (years)**	65.4±13.7	60.4±9.7
**Female (%)**	3 (20.0)	3 (21.4)
**Etiology***		
**HBV**	2 (13.3)	2 (14.3)
**HCV**	5 (33.3)	3 (21.4)
**Alcohol-related**	1 (6.7)	5 (35.1)
**MASLD/MASH**	2 (13.3)	1 (7.1)
**Other**	3 (20.0)	2 (14.3)
**Unknown**	2 (13.3)	2 (14.3)
**Child-Pugh category (%)**		
**A**	8 (53.3)	5 (35.7)
**B**	7 (46.7)	9 (64.3)
**Ascites (%)**		
**None**	10 (66.7)	6 (42.9)
**Present**	5 (33.3)	8 (57.1)
**Hepatic encephalopathy**		
**None**	15 (100)	12 (85.7)
**Present**	0 (0)	2 (14.3)
**ECOG PS ≥ 2 (%)**	2 (13.3)	2 (14.3)
**BCLC stage (%)**		
**B**	3 (20.0)	2 (14.3)
**C**	12 (80.0)	12 (85.7)
**Extrahepatic spread (%)**	10 (66.7)	5 (35.1)
**Macrovascular invasion (%)**	5 (33.3)	8 (57.1)
**ALBI grade**		
**1**	1 (6.7)	2 (14.3)
**2**	7 (46.7)	4 (28.6)
**3**	7 (46.7)	8 (57.1)
**Albumin (mean, mg/dl)**	3.2	3.1
**Bilirubin (mean, mg/dl)**	1.2	2.6
**AFP (mean, ng/ml)**	156271	36001

* = Several etiologies in one patient possible. ALBI, Albumin-Bilirubin; BCLC, Barcelona Clinic Liver Cancer Staging System; ECOG, Eastern Cooperative Oncology Group performance status; SD, standard deviation.

Table S4. Baseline characteristics translational cohort

Variable	EM cohort n=8	Non-EM cohort n=32	P-value
Mean age ± SD (years)	66.8±12.5	66.2±8.0	0.95
Female (%)	2 (25.0)	3 (9.4)	0.26
Etiology (%)			0.65
Viral	1 (12.5)	9 (28.1)	
Non-Viral	7 (87.5)	23 (71.9)	
Cirrhosis (%)	5 (62.5)	23 (71.9)	0.53
Child-Pugh (%)*			
A	3 (37.5)	28 (87.5)	**0.008**
B	5 (62.5)	4 (12.5)	**0.008**
C	0 (0.0)	0 (0.0)	>0.99
Ascites (%)			0.66
yes	3 (37.5)	8 (25.0)	
no	5 (62.5)	24 (75.0)	
Hepatic encephalopathy (%)			0.10
yes	2 (25.0)	1 (3.1)	
no	6 (75.0)	31 (96.9)	
ECOG PS ≥ 2 (%)	0 (0.0)	1 (3.1)	>0.99

BCLC stage (%)			
A	0 (0)	0 (0.0)	>0.99
B	2 (25.0)	7 (21.9)	>0.99
C	6 (75.0)	25 (78.1)	>0.99
D	0 (0.0)	0 (0.0)	>0.99
Extrahepatic spread (%)	3 (37.5)	18 (56.3)	0.44
Macrovascular invasion (%)	3 (37.5)	13 (40.6)	>0.99
Gastroesophageal varices (%)	4 (50.0)	9 (28.1)	0.40
Esophageal varices grade (%)			
none	4 (50.0)	23 (71.9)	0.40
I	4 (50.0)	8 (25.0)	0.21
II	0 (0.0)	0 (0.0)	>0.99
III	0 (0.0)	1 (3.1)	>0.99
Prior variceal bleeding (%)	1 (12.5)	0 (0.0)	0.20
NSBB (%)	2 (25.0)	3 (9.4)	0.26
Antiplatelet drugs (%)	1 (12.5)	8 (25.0)	0.66
Anticoagulation (%)	4 (50.0)	7 (21.9)	0.18

Baseline characteristics for translational cohort: early mortality versus non-early mortality. Continuous variables were reported as mean plus standard deviation and compared via independent samples t-test or Mann-Whitney U test, depending on the presence of a normal distribution. Categorical variables were reported as numbers and percentages. Comparisons of categorical variables were conducted by Fisher's exact test. P <0.05 was considered statistically significant. BCLC, Barcelona Clinic Liver Cancer Staging System; ECOG, Eastern Cooperative Oncology Group performance status; EM, early mortality; HCC, hepatocellular carcinoma; NSBB, non-selective beta blockers, SD, standard deviation.

Table S5. Best response in the early mortality cohort and non-early mortality cohort

**Variable**	**Early mortality cohort**<br>**n=50**	**Non-early mortality cohort**<br>**n=267**
**Best overall response n (%)**	1 (2)	91 (34.1)
**Complete response n (%)**	0 (0)	9 (3.4)
**Partial response n (%)**	1 (2)	82 (30.7)
**Stable disease n (%)**	10 (20)	86 (32.2)
**Disease control rate n (%)**	11 (22)	177 (66.3)
**Progressive disease n (%)**	7 (14)	74 (27.7)
**Missing n (%)**	32 (64)	16 (6)

Table S5. Baseline characteristics of patients experiencing EM with Child Pugh A or Child Pugh B

	Child-Pugh A n=25	Child-Pugh B n=25
**Variable**		
**Mean age ± SD (years)**	66.96±12.57	66.84±10.61
**Female (%)**	4 (16.0)	4 (16.0)
**Etiology***		
**HBV**	3 (12.0)	3 (12.0)
**HCV**	7 (28.0)	3 (12.0)
**Alcohol-related**	5 (20.0)	8 (32.0)
**MASLD/MASH**	5 (20.0)	0 (0)
**Other**	2 (8.0)	1 (4.0)
**Unknown**	3 (12.0)	11 (44.0)
**Ascites (%)**		
**None**	22 (88.0)	11 (44.0)
**Present**	2 (8.0)	14 (56.0)
**Missing**	1 (4.0)	0 (0)
**Hepatic encephalopathy**		
**None**	24 (96.0)	22 (88.0)
**Present**	0 (0)	3 (12.0)
**Missing**	1 (4.0)	0 (0)
**ECOG PS ≥ 2 (%)#**	1 (4.0)	7 (28.0)
**BCLC stage (%)**		
**B**	3 (12.0)	4 (16.0)
**C**	22 (88.0)	21 (84.0)
**Extrahepatic spread (%)#**	9 (36.0)	13 (52.0)
**Macrovascular invasion (%)#**	13 (52.0)	14 (56.0)
**ALBI grade**		
**1**	2 (8.0)	1 (4.0)
**2**	18 (72.0)	5 (20.0)
**3**	3 (12.0)	19 (76.0)
**Missing**	2 (8.0)	0 (0)
**Albumin (mean, mg/dl)**	3.5	2.9
**Bilirubin (mean, mg/dl)**	1.1	2.5
**AFP (mean, ng/ml)**	88948	24535

Continuous variables were reported as mean plus standard deviation and compared via independent samples t-test or Mann-Whitney U test, depending on the presence of a normal distribution. Categorical variables were reported as numbers and percentages. Comparisons of categorical variables were conducted by Fisher's exact test. P <0.05 was considered statistically significant. * = Several etiologies in one patient possible. # = In one patient, no data was available. BCLC, Barcelona Clinic Liver Cancer Staging System; ECOG, Eastern Cooperative Oncology Group performance status; EM, early mortality; HCC, hepatocellular carcinoma; NSBB, non selective beta blockers, SD, standard deviation.

Table S6. Univariate analysis of risk factors for 90 days early mortality in atezo/bev-treated patients with Child-Pugh A

Variable	Univariate Odd's ratio	95% (CI)	p-value
Age	0.9901	0.9548 to 1.029	0.6007
Sex (male)	1.537	0.5522 to 5.457	0.4508
ECOG PS ≥ 2	0.5472	0.02963 to 2.893	0.5677
Nonviral etiology	1.678	0.6937 to 3.914	0.2359
CPS A6 (A5 as reference)	4.235	1.773 to 10.90	**0.0016**
Ascites (no ascites as reference)	0.5515	0.08564 to 2.008	0.4363
BCLC C (B as reference)	2.204	0.7243 to 9.585	0.2144
Extrahepatic spread	0.7677	0.3101 to 1.804	0.5515
Macrovascular invasion	1.990	0.8499 to 4.738	0.1126
Presence of GE varices	1.643	0.6869 to 3.969	0.2616
History of variceal bleeding	2.217	0.3220 to 9.520	0.3318
Spleen size	1.143	0.9873 to 1.323	0.0698
NSBB	1.565	0.6072 to 3.749	0.3285
Anticoagulation	1.330	0.5378 to 3.115	0.5193
Albumin g/dl	0.1741	0.0626 to 0.442	**0.0004**
Bilirubin md/gl	2.205	1.017 to 4.635	**0.0382**
ALBI Grade 2 (Grade 1 as reference)	5.763	1.599 to 36.92	**0.0212**
ALBI Grade 3 (Grade 1 as reference)	15.64	2.251 to 135.5	**0.0057**
INR	4.347	0.2238 to 67.70	0.3081
Creatinine mg/dl	0.9497	0.2561 to 2.638	0.9301
Presence of proteinuria	1.064	0.3208 to 3.081	0.9132
CRP mg/dl	1.072	0.9768 to 1.169	0.1064
AFP ng/ml	1.000	1.000 to 1.000	0.1117
Leucocytes 10^9/L	0.9664	0.8096 to 1.135	0.6909
Neutrophils 10^9/L	0.9636	0.7886 to 1.033	0.5743
Platelets 10^9/L	1.002	0.9981 to 1.005	0.3251
Hemoglobin g/dl	0.7473	0.5993 to 0.9252	**0.0081**
MELD score	1.074	0.9672 to 1.193	0.1800
Neutrophils to platelets ratio	0.9938	0.9567 to 1.004	0.6211

Odd's ratio for early mortality risk was assessed by univariate logistic regression. Abbreviations: ALBI, Albumin-Bilirubin; BCLC, Barcelona Clinic Liver Cancer; CI, confidence interval; CPS, Child-Pugh score; ECOG, Eastern Cooperative Oncology Group Performance Status; GE varices, gastoesophageal varices; MELD, Model of Endstage Liver Disease; NSBB, non-selective beta-blockers.

Table S7. Multivariate analysis of risk factors for 90 days early mortality in atezo/bev-treated patients with Child-Pugh A

Variable	Univariate Odd's ratio	95% (CI)	p-value
ALBI Grade 2 (Grade 1 as reference)	4.382	1.167 to 28.58	0.0566
ALBI Grade 3 (Grade 1 as reference)	12.54	1.746 to 111.0	**0.0123**
Hemoglobin g/dl	0.7957	0.6308 to 0.9973	**0.0488**

Odd's ratio for early mortality risk was assessed by multivariate logistic regression. Abbreviations: ALBI, Albumin-Bilirubin; CI, confidence interval.

Table S8. Univariate analysis of risk factors for 90 days early mortality in atezo/bev-treated patients with Child-Pugh B

Variable	Univariate Odd's ratio	95% (CI)	p-value
Age	1.006	0.9621 to 1.055	0.7859
Sex (male)	0.5397	0.1368 to 1.783	0.3356
ECOG PS ≥ 2	4.324	1.156 to 18.36	**0.0337**
Nonviral etiology	0.8088	0.2559 to 2.718	0.7213
CPS B8 (B7 as reference)	1.175	0.3415 to 3.826	0.7912
CPS B9 (B7 as reference)	4.073	1.120 to 16.23	**0.0366**
Ascites. moderate (no ascites as reference)	0.6719	0.2290 to 1.944	0.4628
Ascites. severe (no ascites as reference)	1.030	0.2204 to 4.476	0.9684
Hepatic encephalopathy	1.432	0.2628 to 7.057	0.6568
BCLC C (B as reference)	1.853	0.5609 to 7.309	0.3356
Extrahepatic spread	1.685	0.6312 to 4.564	0.2981
Macrovascular invasion	1.069	0.4014 to 2.886	0.8937
Presence of GE varices	0.6111	0.2189 to 1.682	0.3404
History of variceal bleeding	3.000	0.4652 to 24.06	0.2472
Spleen size	0.9005	0.7380 to 1.084	0.2792
NSBB	0.4242	0.1416 to 1.175	0.1086
Anticoagulation	1.692	0.6010 to 4.749	0.3148
Albumin g/dl	0.1787	0.0497 to 0.528	**0.0040**
Bilirubin md/gl	1.406	1.011 to 2.138	0.0714
ALBI Grade 2 (Grade 1 as reference)	0.1667	0.00589 to 4.664	0.2305
ALBI Grade 3 (Grade 1 as reference)	1.267	0.04746 to 33.82	0.8710
INR	0.2607	0.01774 to 1.462	0.2203
Creatinine mg/dl	6.739	1.839 to 36.66	**0.0120**
Presence of proteinuria	1.037	0.1984 to 4.415	0.9623
CRP mg/dl	1.209	1.066 to 1.424	**0.0092**
AFP ng/ml	1.000	1.000 to 1.000	0.0522
Leucocytes 10^9/L	1.275	1.090 to 1.545	**0.0061**
Neutrophils 10^9/L	1.323	1.080 to 1.674	**0.0116**
Platelets 10^9/L	1.005	1.002 to 1.010	**0.0054**
Hemoglobin g/dl	0.9939	0.7999 to 1.234	0.9551
MELD score	1.120	1.010 to 1.257	**0.0403**
Neutrophils to platelets ratio	0.9933	0.9695 to 1.016	0.5334

Odd’s ratio for early mortality risk was assessed by univariate logistic regression. Abbreviations: ALBI, Albumin-Bilirubin; BCLC, Barcelona Clinic Liver Cancer; CI, confidence interval; CPS, Child-Pugh score; ECOG, Eastern Cooperative Oncology

Group Performance Status; GE varices, gastoesophageal varices; MELD, Model of Endstage Liver Disease; NSBB, non-selective beta-blockers.

Table S9. Multivariate analysis of risk factors for 90 days early mortality in atezo/bev-treated patients with Child-Pugh B

Variable	Univariate Odd's ratio	95% (CI)	p-value
CPS B9	5.556	1.342 to 25.15	0.0196
ECOG PS ≥ 2	9.382	1.966 to 53.73	0.0070
Creatinine mg/dl	9.301	1.670 to 85.24	0.0292

Odd's ratio for early mortality risk was assessed by multivariate logistic regression. Abbreviations: CI, confidence interval; CPS, Child-Pugh score; ECOG, Eastern Cooperative Oncology Group Performance Status.
